# Trend and projection of skilled birth attendants and institutional delivery coverage for adolescents in 54 low- and middle-income countries, 2000–2030

**DOI:** 10.1186/s12916-022-02255-x

**Published:** 2022-02-04

**Authors:** Md. Mizanur Rahman, Hiroko Taniguchi, Raïssa Shiyghan Nsashiyi, Rashedul Islam, Syed Riaz Mahmud, Shafiur Rahman, Jenny Jung, Shahjahan Khan

**Affiliations:** 1 Hitotsubashi Institute for Advanced Study, University of Hitotsubashi, 2-1 Naka, Kunitachi Tokyo, 186-8601 Japan; 2 Department of Global Health Policy, School of International Health, The University of Tokyo, Tokyo, Japan; 3 Institute for Nature, Health, and Agricultural Research (INHAR), Yaoundé, Cameroon; 4 Global Public Health Research Foundation, Dhaka, Bangladesh; 5 Hamamatsu University School of Medicine, Hamamatsu, Shizuoka Japan; 6 School of Sciences, Centre for Health Research, University of Southern Queensland, Toowoomba, Australia

**Keywords:** Institutional delivery, Skill birth attendants, Delivery care, Inequalities, Prediction, Adolescent, Bayesian model

## Abstract

**Background:**

Limitations to accessing delivery care services increase the risks of adverse outcomes during pregnancy and delivery for all pregnant women, particularly among adolescents in LMICs. In order to inform adolescent-specific delivery care initiatives and coverage, we conducted a comprehensive analysis of trends, projections and inequalities in coverage of delivery care services among adolescents at national, urban-rural and socio-economic levels in LMICs.

**Methods:**

Using 224 nationally representative cross-sectional survey data between 2000 and 2019, we estimated the coverage of institutional delivery (INSD) and skilled birth attendants (SBA). Bayesian hierarchical regression models were used to estimate trends, projections and determinants of INSD and SBA.

**Results:**

Coverage of delivery care services among adolescents increased substantially at the national level, as well as in both urban and rural areas in most countries between 2000 and 2018. Of the 54 LMICs, 24 countries reached 80% coverage of both INSD and SBA in 2018, and predictions for 40 countries are set to exceed 80% by 2030. The trends in coverage of INSD and SBA of adult mothers mostly align with those for adolescent mothers. Our findings show that urban-rural and wealth-based inequalities to delivery care remain persistent by 2030. In 2018, urban settings across 54 countries had higher rates of coverage exceeding 80% compared to rural for both INSD (45 urban, 16 rural) and SBA (50 urban, 19 rural). Several factors such as household head age ≥ 46 years, household head being female, access to mass media, lower parity, higher education, higher ANC visits and higher socio-economic status could increase the coverage of INSD and SBA among adolescents and adult women.

**Conclusions:**

More than three-quarters of the LMICs are predicted to achieve 80% coverage of INSD and SBA among adolescent mothers in 2030, although with sustained inequalities.

**Supplementary Information:**

The online version contains supplementary material available at 10.1186/s12916-022-02255-x.

## Background

Enforcing measures that ensure healthy lives and promote well-being for all people across all ages, including delivery care services for the vulnerable group of pregnant adolescent women (aged 15–19 years), is a core goal among Sustainable Development Goals (SDG), specifically SDG 3, adopted by the United Nations [1]. Delivery care services are more crucial for the survival of adolescents as they often exhibit more severe complications from pregnancy and childbirth, such as maternal anaemia, gestational hypertension, malnutrition, eclampsia, preterm birth and low birth weight [2, 3]. These complications increase the chances of adolescent perinatal mortalities, which currently ranks second among leading causes of death worldwide [2, 3]. In low- and middle-income countries (LMICs) across Africa and Asia, where access to delivery care services are limited and of poorer quality [4], predispositions to perinatal mortality in adolescence are even more heightened [2, 3].

Countermeasure strategies at the global and national levels were put in place before and after the era of Millennium Development Goals (MDGs) such as the Safe Motherhood Initiative, the Basic Package of Health Services (BPHS) and the Essential Package of Health Services (EPHS) to improve essential maternal and child healthcare. However, recent assessments have revealed that LMICs bear a substantial burden of adolescent pregnancies, with an estimated 21 million pregnancies occurring among girls aged 15–19 years, per annum [5, 6]. Other reports have also indicated that adolescent pregnancy in LMICs makes up 95% of the yearly global totals [7, 8]. In the face of these, the public health burden related to adolescent pregnancy has worsened in most LMICs [9] as care services for them remain highly fragmented and poorly coordinated, thus hindering access to delivery care services for pregnant adolescents [10, 11]. As evident from many research studies [12, 13], improving access to a continuum of critical delivery care services, including skill birth attendants (SBA) and institutional delivery (INSD), are important for increasing rates of postnatal visits, and consequently, their positive outcomes of reduced pregnancy and childbirth complications and reduced maternal and neonatal mortality.

Particularly, for pregnant adolescents in LMICs, access to delivery care services is hampered by issues ranging from availability, affordability and acceptability [10, 11], or general reluctance of adolescents to seeking care [4]. In particular, affordability is a significant barrier for adolescents to access delivery care given their likelihood for greater economic dependency on parents or guardians. Complexities in understanding delivery care uptake among adolescents in LMICs necessitate comprehensive assessments of geographical patterns and socio-economic influences, which importantly could also inform a broad range of adolescent-specific delivery care initiatives. To date, previous research has mostly focused on the provision of antenatal care services using primary data and present findings for all pregnant women of reproductive age (15–49 years) [13–17] who generally have better pregnancy and childbirth outcomes compared to adolescents [4]. A gap in the literature exists to assess the use and determinants of delivery care services particularly for adolescent mothers, and further using nationally representative datasets. Multi-country and country-specific assessments of the coverage of delivery care services, particularly focusing on the equity in access for adolescents, would provide important metrics for global-, regional-, and country-level policies and programmes aimed at improving access to delivery care services, and consequently the health and well-being of adolescent mothers and their children. Therefore, the objectives of this study are to estimate the recent trends in coverage of INSD and SBA during delivery among adolescents and to derive projections of these two indicators up to 2030 for 54 LMICs at the national level, as well as by socio-economic status and area of residence. This study further assesses the determinants and magnitude of wealth-based inequalities in coverage to delivery care services and provides comparative findings for women of different age groups.

## Methods

### Data sources

This study used Demographic Health Survey (DHS) (https://dhsprogram.com/data/available-datasets.cfm) and Multiple Indicator and Cluster Survey (MICS) (https://mics.unicef.org/surveys) carried out since 2000 with at least a single data point. The estimates presented in this study were based on 224 survey data of delivery care prevalence between 2000 and 2019 from 54 low- and middle-income countries (LMICs). Details on the 54 countries including brief survey characteristics are presented in Fig. S1 and Additional file 1: Table 1.

**Table 1:** National level coverage of delivery care visits among adolescents aged 15–19 years in LMICs, 2000–2030

Country	INSD (predicted coverage, 95% CrI)			SBA (predicted coverage, 95% CrI)		
	2000	2018	2030	2000	2018	2030
**South Asia**						
Afghanistan	4.7 (1.8–10.0)	62.7 (47.5–75.6)	94.6 (85.1–98.7)	7.7 (3.1–15.1)	63.8 (48.7–76.0)	93.2 (82.3–98.1)
Bangladesh	7.8 (4.6–12.9)	51.4 (37.4–64.8)	84.6 (65.1–95.0)	10.3 (5.8–16.4)	58.2 (44.8–71.3)	87.9 (71.9–96.5)
India	29.8 (16.9–45.8)	84.3 (74.2–91.8)	96.5 (91.6–99.0)	40.4 (24.9–57.7)	85.2 (74.0–92.0)	95.7 (88.3–98.8)
Maldives	62.2 (33.8–86)	98.3 (96.9–99.2)	99.8 (99.4–100)	88.5 (72.7–96.4)	99.8 (99.6–99.9)	100.0 (99.9–100)
Nepal	14.2 (8.5–21.6)	72.0 (59.9–81.5)	93.9 (86.1–97.9)	18.4 (10.9–27.9)	71.4 (58.9–81.4)	92.3 (81.2–97.4)
Pakistan	25.6 (10.0–50.6)	68.3 (55.3–79.9)	87.5 (71.8–96.3)	35.0 (13–62.6)	71.7 (59.3–83.3)	87.2 (70.1–96.2)
**East Asia and the Pacific**						
Cambodia	12.2 (7.6–18.1)	93.3 (88.6–96.4)	99.7 (99.1–99.9)	34.5 (23.9–46.5)	95.0 (91.5–97.3)	99.5 (98.6–99.9)
Indonesia	25.5 (14.5–39)	70.6 (58.0–81.5)	89.2 (75.8–96.4)	52.8 (36.7–67.3)	87.3 (80.2–92.7)	95.6 (89.7–98.8)
Laos	9.1 (3.9–18.1)	61.9 (47.7–74.6)	90.7 (77.6–97.3)	15.7 (9.4–24.2)	58.8 (45.3–71.8)	84.6 (69.0–93.7)
Myanmar	26.4 (5.6–61.6)	47.3 (29.9–65.9)	62.5 (23.9–90.2)	55.9 (20.7–86.8)	69.2 (50.9–83.6)	74.2 (36.2–94.9)
Papua New Guinea	34.1 (3.4–82.4)	67.1 (50.5–81.7)	83.1 (48.4–97.4)	38.6 (7–80.3)	65.9 (46.1–80.9)	80.2 (50.1–95.7)
Philippines	24.0 (13.8–36.9)	81.2 (71.9–88.5)	95.9 (90.5–98.6)	50.3 (34.6–66.6)	86.1 (75.8–93.1)	95.0 (86.6–98.8)
Timor-Leste	8.7 (2.2–23.5)	60.3 (42.4–76.6)	89.5 (65.2–98.7)	17.5 (4.2–41.2)	69.9 (52.7–83.5)	91.1 (69.4–98.9)
Vietnam	77.5 (65.3–86.9)	80.9 (68.7–89.4)	81.6 (58.9–94.8)	70.4 (59.5–79.9)	89.7 (82.5–94.8)	95.1 (87.3–98.6)
**Eastern and Southern Africa**						
Angola	29.4 (15.9–46.8)	43.9 (28.5–59.5)	53.0 (23.4–80.6)	20.8 (2–67.1)	48.1 (31.1–65.7)	69.0 (22.3–96.0)
Burundi	25.7 (13.2–41.1)	90.9 (84.7–94.8)	98.9 (96.6–99.7)	25.2 (16.4–35.7)	92.4 (87.6–95.6)	99.3 (98.2–99.8)
Comoros	48.0 (12.7–84.2)	83.8 (66.9–94.1)	91.4 (61.5–99.6)	62.7 (47.9–75.1)	90.3 (81.5–95.7)	96.4 (89.2–99.3)
Ethiopia	3.9 (2.2–6.5)	42.0 (29.4–55.6)	82.6 (65.1–93.3)	7 (4–11.7)	39.0 (25.3–53.2)	72.1 (47.4–88.7)
Kenya	50.7 (37.4–63.2)	69.5 (53.9–81.9)	77.3 (48.5–94.3)	37.7 (26.3–50.1)	76.0 (61.5–87.2)	90.0 (71.3–97.8)
Lesotho	51.7 (33.9–68.9)	83.1 (70.5–91.4)	92.4 (77.2–98.4)	54.3 (34.7–73.1)	84.7 (73.3–92.5)	93.4 (79.4–98.7)
Madagascar	19.2 (8.6–35.9)	58.0 (30.8–83.0)	79.5 (38.9–97.9)	37.9 (24.5–53.1)	62.4 (38.8–82.8)	77.0 (42.8–95.7)
Malawi	45.0 (33.6–56.7)	95.0 (92.0–97.1)	99.3 (98.4–99.7)	49.9 (37.9–61.5)	91.9 (87.7–95.3)	98.2 (96.1–99.3)
Mozambique	51.1 (34.6–66)	76.4 (64.0–85.8)	86.4 (68.6–95.6)	51.8 (35–70.2)	73.9 (60.8–84.7)	83.3 (61.3–94.9)
Rwanda	33.9 (23.2–46.6)	95.1 (91.4–97.5)	99.5 (98.7–99.9)	43 (30.4–55.8)	94.7 (91.0–97.2)	99.3 (98.0–99.8)
Tanzania	45.2 (26.4–64.4)	71.4 (57.1–83.6)	82.9 (61.3–95.7)	44.2 (26.3–64.8)	72.5 (58.6–84.0)	84.7 (64.2–95.6)
Uganda	44.7 (30.8–58.6)	78.2 (67.1–86.7)	90.3 (78.2–96.7)	46.4 (32.4–61.3)	78.9 (67.1–87.5)	90.7 (78.8–96.9)
Zambia	39.4 (25.3–54.2)	85.4 (78.2–91.3)	96.1 (91.4–98.6)	39.2 (25.3–53.2)	82.4 (72.7–89.1)	94.6 (88.4–98.0)
Zimbabwe	50.4 (31.5–69.3)	80.9 (70.7–88.1)	91.1 (77.4–97.7)	55.5 (36.2–73)	80.1 (69.3–88.3)	89.6 (72.6–97.1)
**West and Central Africa**						
Benin	71.0 (52.7–84.9)	88.8 (83.5–92.9)	93.8 (84.2–98.5)	72.8 (56.2–86.6)	81.6 (73.4–87.8)	86.5 (66.3–96.0)
Burkina Faso	42.2 (27.2–58.1)	85.3 (70.2–94.0)	94.8 (81.2–99.4)	52.9 (36.3–69.6)	83.8 (69.6–93.7)	92.3 (75.2–99.0)
Cameroon	49.9 (32.5–68.1)	65.8 (48.9–79.8)	74.3 (42.0–93.1)	49.2 (37.1–61.9)	69.8 (55.6–81.1)	80.7 (59.2–93.2)
Central African Republic	41.4 (19.6–66.2)	57.0 (28.3–81.1)	65.4 (16.3–96.1)	41.8 (28.9–54.7)	59.3 (39.2–77.7)	69.9 (35.9–91.8)
Chad	10.3 (4.6–18.1)	29.6 (18.4–44.7)	50.4 (22.7–80.8)	14.1 (8.7–21.4)	34.1 (20.9–47.8)	52.7 (27.2–75.4)
Congo	78.2 (62.0–89.3)	92.8 (87.2–96.2)	96.3 (88.5–99.3)	85.7 (74.2–93.6)	93.0 (87.9–96.4)	95.2 (85.9–99.0)
Cote d’Ivoire	41.4 (20.9–63.6)	75.0 (61.3–85.3)	87.8 (68.1–96.8)	43.1 (23.2–64.1)	79.0 (67.4–88.6)	91.2 (78.5–97.9)
Democratic Republic of the Congo	58.2 (38.7–76.0)	81.7 (73.6–87.9)	89.8 (75.7–96.8)	64.4 (50.5–76.1)	83.9 (76.8–89.6)	91.3 (81.3–96.6)
Ghana	33.3 (21.1–46.9)	79.3 (67.2–88.1)	93.1 (80.5–98.2)	32 (19.6–44.8)	80.2 (68.5–89.4)	93.9 (83.5–98.6)
Guinea	26.8 (13.6–43.4)	57.1 (44.6–68.5)	75.7 (55.3–90.1)	32.5 (17.3–51.4)	62.1 (50.2–72.7)	78.5 (58.9–91.2)
Liberia	20.8 (7.8–39.3)	79.7 (67.7–88.7)	95.5 (87.4–99.0)	45 (20–71.7)	70.1 (50.1–87.3)	81.5 (47.4–97.5)
Mali	40.2 (25.9–54.9)	73.4 (64.6–81.2)	87.6 (76.5–94.7)	43.6 (29.5–58.2)	72.5 (62.4–81.1)	86.0 (72.9–93.7)
Niger	14.3 (7.8–23)	52.5 (32.3–71.3)	78.1 (49.8–94.3)	24 (14.4–36.6)	53.5 (34.4–71.9)	72.2 (40.6–91.8)
Nigeria	23 (14.4–33.6)	30.5 (22.6–39.0)	36.4 (20.4–55.1)	25.7 (16.1–36.4)	34.1 (25.0–44.5)	40.9 (23.1–62.8)
Sao Tome and Principe	64 (30.2–86.6)	94.9 (89.7–97.8)	98.5 (93.7–99.9)	78 (66.4–87.2)	94.7 (90.4–97.6)	98.1 (94.7–99.6)
Senegal	55.2 (37.3–71.8)	81.3 (73.9–87.4)	90.4 (79.7–96.4)	57.3 (45.1–69.8)	61.2 (51.5–69.8)	63.2 (45.3–78.1)
Sierra Leone	9.2 (4.5–16.3)	78.2 (68.0–86.4)	97.3 (93.5–99.2)	35.7 (24.7–47.8)	76.9 (66.4–85.2)	91.5 (83.0–96.7)
The Gambia	33.9 (18.5–52.9)	80.8 (71.3–87.8)	94.2 (85.0–98.4)	38.9 (21.5–58.3)	81.3 (71.6–89.0)	93.7 (84.5–98.3)
Togo	74.5 (56.3–88.9)	70.5 (51.8–84.1)	66.0 (28.2–91.6)	56 (33.2–77.5)	64.9 (46.7–79.9)	69.4 (35.4–92.1)
**Latin America and Caribbean**						
Dominican Republic	96.9 (91.5–99.1)	99.2 (98.3–99.7)	99.6 (98.6–100)	97.7 (94.1–99.3)	99.3 (98.6–99.7)	99.7 (98.8–100)
Haiti	23.8 (10.5–42.2)	45.5 (30.9–60.3)	63.9 (34.2–87.6)	25.8 (11.7–45)	47.0 (32.1–62.8)	65.3 (35.9–87.9)
Honduras	59.9 (35.8–80.3)	90.9 (81.4–96.5)	96.5 (86.0–99.6)	64.8 (43.6–82.8)	89.9 (79.8–95.9)	95.4 (84.0–99.4)
**Central and Eastern Europe**						
Albania	86.7 (70.5–95.7)	99.6 (99.3–99.8)	100 (99.9–100)	98 (94.8–99.4)	99.9 (99.8–99.9)	100.0 (100–100)
Armenia	99.8 (99.7–99.9)	99.9 (99.8–99.9)	99.9 (99.8–100)	99.8 (99.6–99.9)	99.9 (99.8–100)	99.9 (99.8–100)
Kyrgyzstan	97.6 (95.2–99.0)	99.8 (99.6–99.9)	100.0 (99.9–100)	99.4 (98.7–99.8)	99.8 (99.6–99.9)	99.9 (99.7–100)
Tajikistan	64.2 (44.2–80.3)	92.1 (87.1–95.8)	97.5 (93.5–99.4)	80.8 (71–88.4)	92.8 (87.9–96.3)	96.5 (91–98.9)

*INSD* institutional delivery, *SBA* skilled birth attendants, *CrI* credible intervals

### Outcome variables

INSD and SBA are the outcome variables in this study. The detailed definitions and calculation procedure of the coverage of INSD and SBA is presented in the additional file: e-method 1 [18]. If the woman had more than one birth, outcomes from the most recent birth were used.

### Predictor variables

The predictor variables are different for the outcomes since all analyses are performed in the context of the assessed indicators. For trend and projection analysis, we used country- and year-specific Sociodemographic Index (SDI) and Human Resource for Health (HRH) to estimate the trends and projections of SBA and INSD for each country. The SDI was the preferred variable to measure the level of socio-economic development which captures income, education, and fertility. SDI and HRH were obtained from IHME’s Global Health Data Exchange (GHDx) (http://ghdx.healthdata.org/) and Global Health Observatory (https://www.who.int/data/gho). For the determinants of health service analysis, the following were included in the study based on the availability, comparability and consistency with previous literature [14, 19–22]: age (< 30 years, 30–45 years, 46–60 years, > 60 years) and sex of household head (male or female), adolescent’s level of education (no education, primary, secondary, higher), parity (1, 2, 3, ≥ 4), access to mass media (none, less than once a week, at least once a week), frequency of ANC visits (none, 1, 2, 3, ≥ 4), household wealth quintile (Q1 (poorest), Q2, Q3, Q4, Q5(richest)) and area of residence (urban or rural). These variables were obtained from the DHS and MICS datasets.

### Statistical analysis

We fitted the INSD and SBA models using the Bayesian approach, sampling from the posterior distribution of the parameters using Gibbs Monte Carlo, a Markov chain Monte Carlo (MCMC) method, as implemented in the algorithm in JAGS open-source software (version 4.2). In the MCMC algorithm, we used 10,000 iterations with three chains, 10 thinning and 500 sample burn-in. Non-informative priors were used. Trace plot and Gelman-Rubin diagnostic statistics were used to check the convergence status. The statistical analysis was done in R 3.2.0. Both covariates and the model’s hierarchical structure influence how data from other countries influence predictions for a given country. We examined the sensitivity of our results by two approaches: [1] the exclusion of country-level covariates (SDI and HRH), and [2] altering priors for the hyperparameters. The detailed information of the model, parameters, prior distribution and sensitivity analysis is presented in the additional file: e-method 2-3 [23–25]. The Slope Index of Inequality (SII) was employed to estimate the magnitude of wealth-based inequalities in coverage of delivery care services for each country over the years. Country-specific percentage change in coverage of INSD and SBA during 2000–2030 was estimated from the predicted values. For the determinant analysis, the Bayesian hierarchical regression model was used with random intercept country. The detailed information is presented in the additional file: e-method 4.

## Results

### Survey characteristics

Data from 224 nationally representative household surveys conducted between 2000 and 2019 were included in this study, representing 54 countries from LMICs. Four countries from Central and Eastern Europe, 8 countries from East Asia and the Pacific, 14 from Eastern and Southern Africa, 3 from Latin America and the Caribbean, 6 countries from South Asia and 19 countries from West and Central Africa regions. The country-specific survey data points with survey types are presented in Fig. S1 and Additional file 1: Table 1.

### National coverage

The country-specific predicted coverage of delivery care services among adolescents at the national level from 2000 to 2030 is presented in Table 1. The details of the country- and year-specific coverage of INSD and SBA among adolescents is presented in Fig. S2-3, and percentage change between 2000 and 2030 by area of residence is presented in the additional file: Table 2. Of the 54 LMICs, 24 countries reached 80% coverage of both INSD and SBA in 2018 and predictions for 40 countries are set to exceed 80% by 2030. Overall, the percentage change in INSD (mean percentage increase 53.3) is lower than SBA (mean percentage increase 69.8) between 2000 and 2030. Increasing coverage of INSD and SBA was recorded in most of countries, with significant increases in some countries including Afghanistan, Bangladesh, Laos, Timor-Leste, and Ethiopia. Several countries including Angola, Cameroon, Central African Republic, Chad, Guinea, Haiti, Kenya, Madagascar, Myanmar, Niger, Nigeria, and Togo are not set to achieve 80% coverage of INSD in 2030. Similar to INSD, SBA coverage predictions for 12 countries including Angola, Central African Republic, Chad, Ethiopia, Guinea, Haiti, Madagascar, Myanmar, Niger, Nigeria, Senegal and Togo remain below 80% in 2030. Among the countries with less than 80% coverage expectations for 2030, some countries show lower coverage among adolescents than among adults. These are Myanmar, Angola, Central African Republic, Nigeria and Togo in INSD, and Angola, Myanmar and Nigeria in SBA (additional file: Table 3-4). Conversely, some countries show higher coverage among adolescents than among adults, such as Chad, Kenya, Madagascar and Niger for INSD and Ethiopia, Madagascar and Chad for SBA (additional file: Table 3-4).

**Table 2:** Determinants of access to facility delivery and skilled birth attendants at births among adolescents, 54 low- and middle-income countries

Characteristics	Odds ratio (95% CrI)	
	INSD	SBA
HH head age (years)		
< 30	1.00	1.00
30–45	1.0 (0.96–1.04)	0.97 (0.93–1.02)
46–60	1.09 (1.05–1.14)	1.12 (1.06–1.17)
> 60	1.16 (1.11–1.21)	1.12 (1.08–1.17)
HH head sex		
Male	1.00	1.00
Female	1.16 (1.10–1.22)	1.13 (1.08–1.17)
Mother education		
No education	1.00	1.00
Primary	1.28 (1.21–1.36)	1.22 (1.16–1.29)
Secondary	1.82 (1.72–1.92)	1.88 (1.78–1.99)
Higher	2.52 (2.36–2.69)	3.89 (3.65–4.13)
Parity		
1	1.00	1.00
2	0.60 (0.57–0.62)	0.62 (0.59–0.65)
3	0.47 (0.45–0.49)	0.57 (0.53–0.61)
≥ 4	0.51 (0.48–0.54)	0.48 (0.45–0.51)
ANC visits		
None	1.00	1.00
1	2.13 (2.03–2.23)	2.53 (2.4–2.66)
2	2.85 (2.74–2.95)	3.10 (2.9–3.31)
3	3.87 (3.71–4.04)	4.60 (4.37–4.84)
≥ 4	5.89 (5.65–6.13)	6.47 (6.16–6.79)
Mass media (watch/listen)		
None	1.00	1.00
Less than once a week	1.11 (1.06–1.18)	1.23 (1.17–1.29)
At least once a week	1.30 (1.25–1.35)	1.33 (1.26–1.39)
Wealth quintile		
Q1 (poorest)	1.00	1.00
Q2	1.33 (1.26–1.40)	1.23 (1.17–1.29)
Q3	1.72 (1.64–1.81)	1.61 (1.52–1.69)
Q4	2.44 (2.32–2.58)	2.36 (2.20–2.52)
Q5 (richest)	3.69 (3.47–3.91)	3.39 (3.11–3.67)
Area of residence		
Urban	1.00	1.00
Rural	0.66 (0.63–0.69)	0.64 (0.60–0.67)
Random intercept var(*u*_0*i*_)= *σ*^2^_*u*0_	1.71 (0.99–2.89)	1.32 (0.77–2.25)

*HH* household, *INSD* institutional delivery, *SBA* skilled birth attendants, *CrI* credible intervals

### Coverage disparities by area of residence

**Fig. 1:**
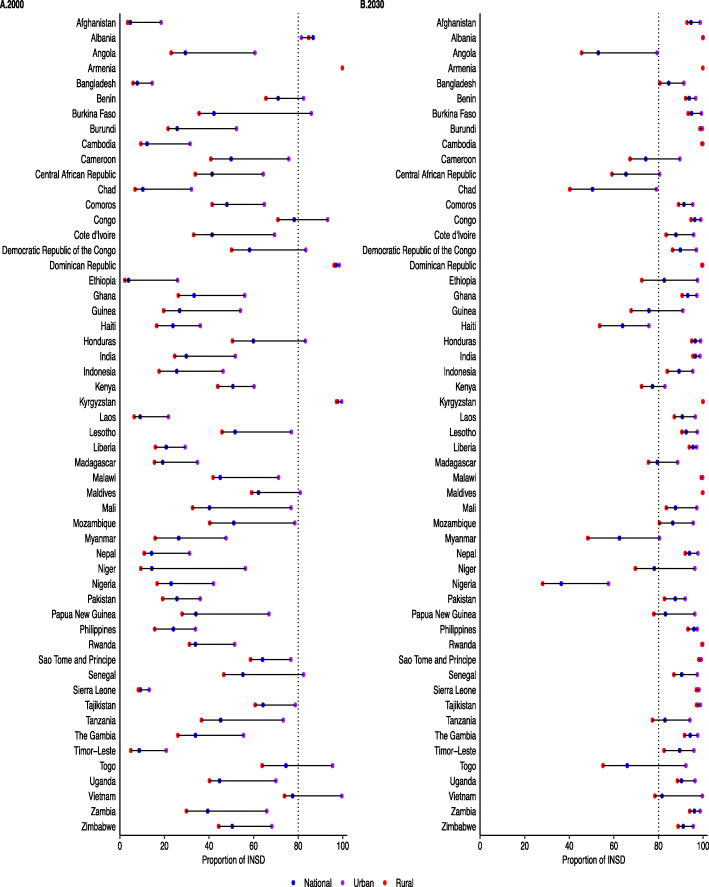
Coverage of institutional delivery among adolescents aged 15–19 according to the area of residence in 54 LMICs, 2000–2030. INSD, institutional delivery

**Fig. 2:**
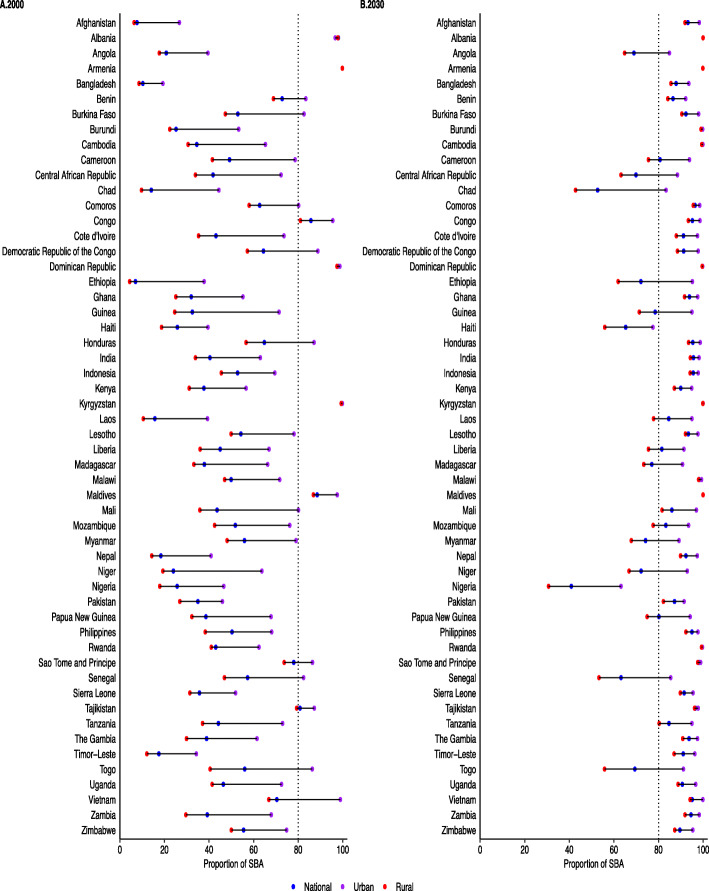
Coverage of skill birth attendants during delivery among adolescents aged 15–19 according to the area of residence in 54 LMICs, 2000–2030. SBA, skilled birth attendants

The urban-rural-specific coverage prediction for INSD and SBA across 54 included countries for 2000 and 2030 are presented in Figs. 1 and 2 as well as in the additional file: Table 5-6. In all countries, coverage of INSD and SBA services among adolescents in urban areas was substantially higher than those in rural areas. Although the coverage of INSD and SBA is increasing rapidly, a wide urban-rural disparity (≥ 30%) was observed in several countries including Angola, Chad, Myanmar, Niger, Nigeria and Togo in 2030 (Figs. 1 and 2). Overall, the increased rates of coverage were relatively bigger in rural areas than in urban areas (additional file: Table 2).

### Socio-economic inequality in coverage of delivery care

**Fig. 3:**
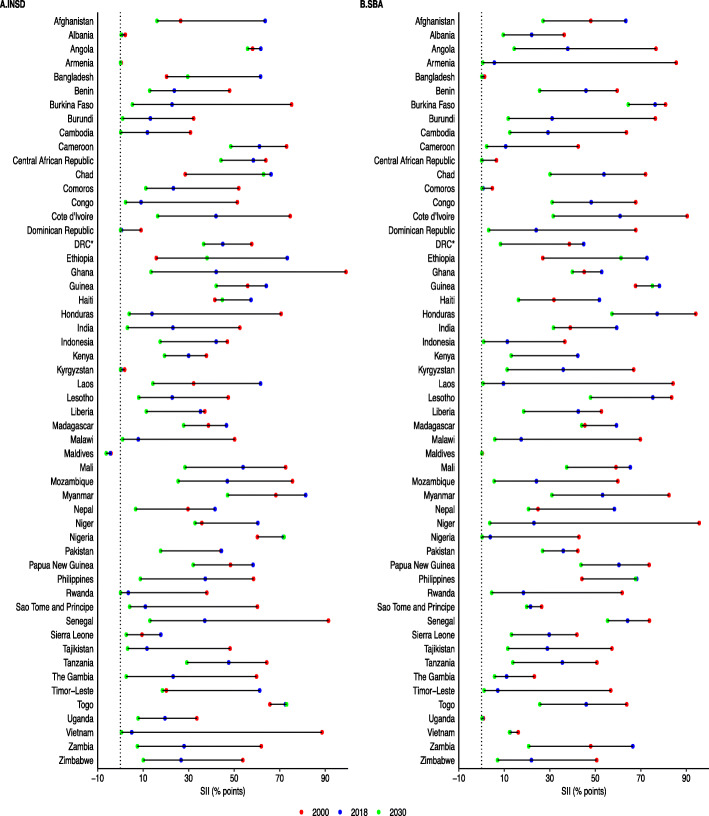
Changes in the magnitude of socio-economic inequality in access to delivery care among adolescents aged 15–19, 2000–2030. SII, slope index of inequality; INSD, institutional delivery; SBA, skilled birth attendant; DRC, Democratic Republic of Congo

The country-specific magnitude of inequalities in coverage of INSD and SBA services among adolescent mothers is presented in Fig. 3. The detailed observable proportion of INSD and SBA for each quintile and SII values for each country are presented in the additional file: Table 7-9. Coverage of delivery care services exhibited pro-rich inequality patterns, i.e. showing higher coverage concentrated among the rich in the population. For both INSD and SBA, more than 50 countries are predicted to have minimal coverage gaps between Q1 and Q5 in 2030 than in 2000. A large reduction of inequalities by around 80% point or more is forecasted for Burkina Faso, Ghana, Senegal and Vietnam in coverage of INSD, and for Armenia, Laos, and Nigeria in coverage of SBA. The 2030 estimates show little to no inequalities in coverage of INSD services observed across nine countries including Albania, Armenia, Burundi, Cambodia, Dominican Republic, Kyrgyzstan, Malawi, Rwanda and Vietnam. For SBA, almost no inequalities were found in ten countries including Armenia, Bangladesh, Central African Republic, Comoros, Indonesia, Laos, Maldives, Nigeria, Timor-Leste and Uganda. The highest pro-rich inequality (around 50% point or more) for INSD is observed in Angola, Cameroon, Chad, Myanmar, Nigeria and Togo. For SBA, the largest inequalities were found in Burkina Faso, Guinea, Honduras, Lesotho and Senegal.

### Determinants of delivery care

Table 2 presents the estimated odds ratio (OR) and 95% credible interval (Crl) for INSD and SBA coverage among adolescents obtained from Bayesian hierarchical regression analysis. Household head age ≥ 46 years, household head being female, higher education level, number of ANC visits, access to mass media and household wealth quintile were associated with increased odds of INSD and SBA. Participants who lived in rural areas had significantly lower odds of INSD and SBA than participants who lived in urban areas. The pattern of determinants was similar in two other age groups of older women, aged 20–35 and 36 or more years (additional file: Table 10-11).

### Sensitivity analysis and model diagnostic

Excluding country-level predictors, the median absolute differences between the two sets of results were very small: 0.35% (2000) and 0.0% (2030) for INSD posterior means; 0.30% (2000) and 0.10% (2030) for country posterior means (additional file: Table 12-13). After altering prior distribution, there were no significant differences in the results (additional file: Table 14-15). In case of model diagnostics, the PSRF values indicated that point estimate and upper limit of PSRF for INSD and SBA model close to 1 (additional file: Table 16-17).

## Discussion

Overall, the study reveals higher levels of SBA compared to INSD coverage across most the settings, which is in line with earlier assessments showing 3% higher coverage in SBA than INSD among women of reproductive age in 80 LMICs [26]. Both INSD and SBA are estimated to have exceeded 80% coverage in nearly half of the countries in 2018, and are predicted to exceed 80% coverage in four-fifth of the countries in 2030. Substantial increases in coverage have been recorded and are predicted in nearly all of the LMICs in Asia and the majority in Africa, where programmes such as the Safe Motherhood Initiative brought about the most improvements in delivery care coverage [26], and where low starting levels provided more latitude for improvements in coverage. Nonetheless, the highest levels of coverage for each of the delivery care services, i.e. 92% or more from 2018 onwards, are observed in all the LMICs in Central and Eastern Europe; likely as a result of better access to and quality of healthcare services, availability and affordability of healthcare coverage [2–4, 9], and also a cultural environment that encourages adolescents’ uptake and thus acceptability of delivery care. Conversely, in countries in Africa and Asia, pregnant adolescents still face considerable culturally entrenched adversities, particularly social stigma and lack of emotional support, that hamper care seeking [4, 9]. Furthermore, aside from the observed increases, the findings of this study underscore the need to minimise inequality arising from rich-poor gap and the urban-rural divide to improve coverage of INSD and SBA. Considerable gaps in delivery care coverage remain in several countries, including Angola, Central African Republic, Chad, Nigeria, Togo in Africa, Myanmar in Asia and Haiti in the Caribbean.

Analyses of urban-rural and wealth-based delivery care coverage show visible inequalities that are predicted to last up to 2030. Our findings across 54 countries found that in 2018, more urban than rural settings had coverage exceeding 80% for both INSD (45 urban, 16 rural) and SBA (50 urban, 19 rural). These findings mostly parallel disparities in delivery care coverage among adolescents in the richest (higher coverage) versus poorest (lower coverage) wealth quintiles. The observed differences are not unexpected as they have similarly been observed in delivery care coverage for women of reproductive age in LMICs [26]. Improving equity in access will require addressing important issues such as the pro-urban and pro-wealth distribution of health services that exists in many LMICs [26], in addition to economic, sociocultural, and environmental factors that limit access to delivery care services for pregnant adolescents, especially the residents in rural areas or in the poorest wealth quintile category.

Policies and programmes in countries with the most significant improvements in INSD and SBA coverage between 2000 to 2030 are Afghanistan, Bangladesh, Laos, Timor-Leste, Ethiopia and Sierra Leone could serve as precedents for improving delivery care coverage for adolescents in countries that are still lagging in progress. Afghanistan, for instance, introduced BPHS in 2002 and the Essential Package of Hospital Services (EPHS) in 2005 for equitable access to healthcare, which brought great increases in numbers of health workers and facilities, followed by increased access to primary healthcare for all, including adolescents [27, 28]. Additionally, with the introduction of ‘maternity waiting homes’, coverage was further expanded particularly in rural areas [29]. In Bangladesh, improvements could be linked to recent initiatives that have focused on the training of additional community-based- and traditional birth attendants amid the high rates of home deliveries (62.2%) [30], as well as mortality-informed (‘Maternal and Perinatal Death Review’) targeting of services quality [27]. Furthermore, a recent systematic review study also suggested that capacity building of healthcare providers on clinical quality, clinical audits and feedback, financial incentives to beneficiaries, pay-for-performance, supportive supervision, community engagement, collaborative efforts and multidimensional interventions approaches help to improve maternal and newborn health services in South Asian countries [31]. Within the last decade, Timor-Leste, Ethiopia and Sierra Leone also implemented innovative strategies mostly targeted toward the mitigation of barriers to coverage due to health personnel shortages [27]. In Cambodia, the substantial narrowing of wealth-based gaps in both INSD and SBA has been attributed to several initiatives, chiefly, the ‘health equity funds’ and the ‘community-based health insurance’ schemes that specifically cover healthcare reimbursements for the poorest in the population [32, 33]. Meanwhile, studies have also shown decreases in delivery care inequalities after the introduction of the National Health Insurance Scheme (NHIS) in Ghana [34, 35].

There are several strengths that characterise this study. This is the first assessment of the trends, projections and inequalities of delivery care service coverage in adolescent mothers in LMICs. The novel findings also include country-level and regional-level analysis, which reveal each country’s in-depth performance in terms of delivery care coverage for pregnant adolescents to serve as a guidance to country-specific adolescent health policies and programmes in the years up to 2030. Secondly, the study utilised the largest number of nationally representative household surveys which provide data on the population, household health, and wealth to examine disaggregated estimates to urban-rural and other different socio-economic groups. Lastly, probability estimates are derived via Bayesian models incorporating the hierarchical framework of the data. On the limitations, given projected outcomes up to the year 2030 are derived based on past trends in delivery care coverage for adolescents, future patterns could alter depending on unpredictable factors, including the potential impact of country-specific delivery care policies, strategies and programme implementations. An important consideration when interpreting these results is that the outcomes, INSD and SBA, are built on self-reported survey data and thus may be subject to recall bias. Additionally, although estimates from DHS and MICS are comparable with the same survey methodologies and samples are nationally representative, we cannot rule out inconsistencies in observed data. In particular, it is not yet clear how and to what degree the COVID-19 pandemic will affect the predictions. Numerous LMICs are facing disruptions to routine antenatal and delivery care services as a result of negative and unintended consequences of COVID-19 strategies. Women, particularly adolescents, are vulnerable to inequities in socio-economic determinants of health which is likely to be heightened by the pandemic. Although our projections for 2030 needs further confirmation from additional research generated from the COVID-19 pandemic, it remains a strong source of evidence to offer valuable insights in understanding the impact of current policies in LMICs.

## Conclusion

A total of 42 out of the 54 LMICs are predicted to achieve 80% coverage of INSD and SBA among adolescent mothers in 2030. However, existing urban-rural and wealth-based inequalities present a major concern in most of the countries. Our study suggested that several factors such as household head age ≥ 46 years, household head being female, lower parity, higher education level, higher number of ANC visits (≥ 4), access to mass media and higher socio-economic status could increase the coverage of INSD and SBA.

## Supplementary Information

**Additional file 1: Figure S1.** Number of data points by country. **Figure S2.** Detail year-specific observed and predicted coverage of INSD by area of residence (national, urban, and rural). **Figure S3.** Detail year-specific observed and predicted coverage of SBA by area of residence (national, urban, and rural). **Table S1.** Country specific data sources. **Table S2.** Percentage change in coverage of INSD and SBA visits between 2000 and 2030. **Table S3.** Coverage of INSD among adult women aged 20-49 years in 54 low- and middle-income countries, 2000-2030. **Table S4.** Coverage of SBA among adult women aged 20-49 years in 54 low- and middle-income countries, 2000-2030. **Table S5.** Coverage of INSD among adolescents according to area of residence in 54 low- and middle-income countries, 2000-2030. **Table S6.** Coverage of SBA among adolescents according to area of residence in 54 low- and middle-income countries, 2000-2030. **Table S7.** Coverage of institutional delivery among adolescent according to wealth quintile in LMICs, 2000-2030. **Table S8.** Coverage of skilled birth attendants among adolescent according to wealth quintile in LMICs, 2000-2030. **Table S9.** Changes in the magnitude of socio-economic inequality in access to delivery care, 2000-2030. **Table S10.** Determinants of access to facility delivery and skilled birth attendants at births among women aged 20-35 years, 54 low-and middle-income countries. **Table S11.** Determinants of access to facility delivery and skilled birth attendants at births among women aged 36 years or more, 54 low-and middle-income countries. **Table S12.** Posterior mean difference by considering with and without country level predictors for INSD. **Table S13.** Posterior mean difference by considering with and without country level predictors for SBA. **Table S14.** Posterior mean difference by altering prior distribution on hyperparameters for INSD. **Table S15.** Posterior mean difference by altering prior distribution on hyperparameters for SBA. **Table S16.** National level estimate of Gelman Rubin Potential scale reduction factors (PSRF) for INSD. **Table S17.** National level estimate of Gelman Rubin Potential scale reduction factors (PSRF) for SBA. **e-method 1.** Definitions and calculation procedure of the coverage of INSD and SBA. **e-method 2.** Bayesian model. **e-method 3.** Sensitivity analysis. **e-method 4.** Bayesian model for determinant analysis.

## Data Availability

The data that support the findings of this study are publicly available from the DHS Program at https://dhsprogram.com/data/available-datasets.cfm and MICS at https://mics.unicef.org/surveys. SDI data were obtained from Global Health Data Exchange (http://ghdx.healthdata.org/search/site/SDI) and HRH from the Global Health Observatory (https://www.who.int/data/gho).

## Abbreviations

ANC: Antenatal care
CrI: Credible interval
DHS: Demographic and Health Survey
HRH: Human Resource for Health
INSD: Institutional delivery
LMICs: Low- and middle-income countries
MCMC: Markov chain Monte Carlo
MICS: Multiple Indicators and Cluster Survey
SBA: Skilled birth attendants
SDI: Sociodemographic index
WHO: World Health Organization

## References

[1] Desa U (2016). Transforming our world: The 2030 agenda for sustainable development.

[2] Gupta N, Kiran U, Bhal K (2008). Teenage pregnancies: obstetric characteristics and outcome. Eur J Obstet Gynecol Reprod Biol.

[3] Jolly MC, Sebire N, Harris J, Robinson S, Regan L (2000). Obstetric risks of pregnancy in women less than 18 years old. Obstetrics & Gynecology.

[4] Field S, Abrahams Z, Honikman S (2020). Adolescent mothers: a qualitative study on barriers and facilitators to mental health in a low-resource setting in Cape Town, South Africa. Afr J Prim Health Care Fam Med.

[5] Darroch J, Woog V, Bankole A, Ashford L (2016). Addiing it up: costs and benefits of meeting the contraceptive needs of adolescents.

[6] World Health Organisation. Adolescent pregnancy 2020 [Available from: https://www.who.int/en/news-room/fact-sheets/detail/adolescent-pregnancy.

[7] Johnson W, Moore SE (2016). Adolescent pregnancy, nutrition, and health outcomes in low- and middle-income countries: what we know and what we don’t know. BJOG..

[8] Kumar M, Huang K-Y, Othieno C, Wamalwa D, Madeghe B, Osok J, Kahonge SN, Nato J, McKay MMK (2018). Adolescent pregnancy and challenges in Kenyan context: perspectives from multiple community stakeholders. Glob Soc Welf.

[9] Pandey A, Kumar GA, Dandona R, Dandona L (2018). Variations in catastrophic health expenditure across the states of India: 2004 to 2014. PLoS One.

[10] Salam RA, Das JK, Lassi ZS, Bhutta ZA (2016). Adolescent health interventions: conclusions, evidence gaps, and research priorities. J Adolesc Health.

[11] Banke-Thomas OE, Banke-Thomas AO, Ameh CA (2017). Factors influencing utilisation of maternal health services by adolescent mothers in low-and middle-income countries: a systematic review. BMC Pregnancy Childbirth.

[12] Keats EC, Ngugi A, Macharia W, Akseer N, Khaemba EN, Bhatti Z, Rizvi A, Tole J, Bhutta ZA (2017). Progress and priorities for reproductive, maternal, newborn, and child health in Kenya: a countdown to 2015 country case study. Lancet Glob Health.

[13] Singh K, Brodish P, Chowdhury ME, Biswas TK, Kim ET, Godwin C, Moran A (2017). Postnatal care for newborns in Bangladesh: the importance of health-related factors and location. J Glob Health.

[14] D’Haenens F, Van Rompaey B, Swinnen E, Dilles T, Beeckman K (2019). The effects of continuity of care on the health of mother and child in the postnatal period: a systematic review. Eur J Public Health.

[15] Kipping RR, Campbell RM, MacArthur GJ, Gunnell DJ, Hickman M (2012). Multiple risk behaviour in adolescence. J Public Health (Oxf).

[16] Organization WH. Broadening the horizon: balancing protection and risk for adolescents: World Health Organization; 2001.

[17] Suhrcke M, de Paz Nieves C (2011). The impact of health and health behaviours on educational outcomes in high-income countries: a review of the evidence.

[18] França GVA, Restrepo-Méndez MC, Maia MFS, Victora CG, Barros AJD (2016). Coverage and equity in reproductive and maternal health interventions in Brazil: impressive progress following the implementation of the Unified Health System. Int J Equity Health.

[19] Arsenault C, Jordan K, Lee D, Dinsa G, Manzi F, Marchant T, Kruk ME (2018). Equity in antenatal care quality: an analysis of 91 national household surveys. Lancet Glob Health.

[20] Laksono AD, Rukmini R, Wulandari RD (2020). Regional disparities in antenatal care utilization in Indonesia. PLoS One..

[21] Tikmani SS, Ali SA, Saleem S, Bann CM, Mwenechanya M, Carlo WA, et al. Trends of antenatal care during pregnancy in low-and middle-income countries: findings from the global network maternal and newborn health registry. In: Seminars in Perinatology: Elsevier; 2019. 10.1053/j.semperi.2019.03.020 PMC7027164 31005357

[22] Victora CG, Requejo JH, Barros AJ, Berman P, Bhutta Z, Boerma T, et al. Countdown to 2015: a decade of tracking progress for maternal, newborn, and child survival. The Lancet. 2016;387(10032):2049–59. 10.1016/S0140-6736(15)00519-X. 10.1016/S0140-6736(15)00519-X PMC7613171 26477328

[23] Danaei G, Finucane MM, Lin JK, Singh GM, Paciorek CJ, Cowan MJ, Farzadfar F, Stevens GA, Lim SS, Riley LM, Ezzati M, Global Burden of Metabolic Risk Factors of Chronic Diseases Collaborating Group (Blood Pressure) (2011). National, regional, and global trends in systolic blood pressure since 1980: systematic analysis of health examination surveys and epidemiological studies with 786 country-years and 5·4 million participants. Lancet..

[24] Ntzoufras I (2011). Bayesian modeling using WinBUGS.

[25] Andrew G (2006). Prior distributions for variance parameters in hierarchical models (comment on article by Browne and Draper). Bayesian Anal.

[26] Graham WJ, Bell JS, Bullough CH (2001). Can skilled attendance at delivery reduce maternal mortality in developing countries? Safe motherhood strategies: a review of the evidence.

[27] Amouzou A, Requejo J, Park L, Peters DH. Achieving maternal and child health gains in Afghanistan: a countdown to 2015 country case study by Akseer et al, 2016. Lancet Glob Health 2016;4:e395-413. J Public Health Emerg 2017;1(1), 10.21037/jphe.2016.12.18. 10.1016/S2214-109X(16)30002-X 27198844

[28] Newbrander W, Ickx P, Feroz F, Stanekzai H (2014). Afghanistan’s basic package of health services: its development and effects on rebuilding the health system. Glob Public Health.

[29] Dickson K, Ekpini R, Higgins-Steele A, Rafique N, Kapeu S (2013). Wenz K, et al.

[30] Saha M, Odjidja EN (2017). Access to a skilled birth attendant in Bangladesh: what we know and what health system framework can teach us. Health Syst Policy Res.

[31] Mian NU, Alvi MA, Malik MZ, Iqbal S, Zakar R, Zakar MZ (2018). Approaches towards improving the quality of maternal and newborn health services in South Asia: challenges and opportunities for healthcare systems. Global Health.

[32] Dingle A, Powell-Jackson T, Goodman C (2013). A decade of improvements in equity of access to reproductive and maternal health services in Cambodia, 2000-2010. Int J Equity Health.

[33] Pierce H (2019). Increasing health facility deliveries in Cambodia and its influence on child health. Int J Equity Health.

[34] Novignon J, Ofori B, Tabiri KG, Pulok MH (2019). Socioeconomic inequalities in maternal health care utilization in Ghana. Int J Equity Health.

[35] Dzakpasu S, Soremekun S, Manu A, Ten Asbroek G, Tawiah C, Hurt L (2012). Impact of free delivery care on health facility delivery and insurance coverage in Ghana’s Brong Ahafo Region. PLoS One..

