# Supplementary material for: Trend and projection of skilled birth attendants and institutional delivery coverage for adolescents in 54 low- and middle-income countries, 2000–2030
Source: BMC Med. 2022 Feb 4;20:46. doi: 10.1186/s12916-022-02255-x (PMC8813474; doi:10.1186/s12916-022-02255-x)
Supplement: Supplementary file 1 — Additional file 1: Figure S1. Number of data points by country. Figure S2. Detail year-specific observed and predicted coverage of INSD by area of residence (national, urban, and rural). Figure S3. Detail year-specific observed and predicted coverage of SBA by area of residence (national, urban, and rural). Table S1. Country specific data sources. Table S2. Percentage change in coverage of INSD and SBA visits between 2000 and 2030. Table S3. Coverage of INSD among adult women aged 20-49 years in 54 low- and middle-income countries, 2000-2030. Table S4. Coverage of SBA among adult women aged 20-49 years in 54 low- and middle-income countries, 2000-2030. Table S5. Coverage of INSD among adolescents according to area of residence in 54 low- and middle-income countries, 2000-2030. Table S6. Coverage of SBA among adolescents according to area of residence in 54 low- and middle-income countries, 2000-2030. Table S7. Coverage of institutional delivery among adolescent according to wealth quintile in LMICs, 2000-2030. Table S8. Coverage of skilled birth attendants among adolescent according to wealth quintile in LMICs, 2000-2030. Table S9. Changes in the magnitude of socio-economic inequality in access to delivery care, 2000-2030. Table S10. Determinants of access to facility delivery and skilled birth attendants at births among women aged 20-35 years, 54 low-and middle-income countries. Table S11. Determinants of access to facility delivery and skilled birth attendants at births among women aged 36 years or more, 54 low-and middle-income countries. Table S12. Posterior mean difference by considering with and without country level predictors for INSD. Table S13. Posterior mean difference by considering with and without country level predictors for SBA. Table S14. Posterior mean difference by altering prior distribution on hyperparameters for INSD. Table S15. Posterior mean difference by altering prior distribution on hyperparameters for SBA. Table S16. National level estima [file 12916_2022_2255_MOESM1_ESM.docx]

**Additional file**

**Trend and projection of skilled birth attendants and institutional delivery coverage for adolescents and women in 54 low-and middle-income countries, 2000-2030**


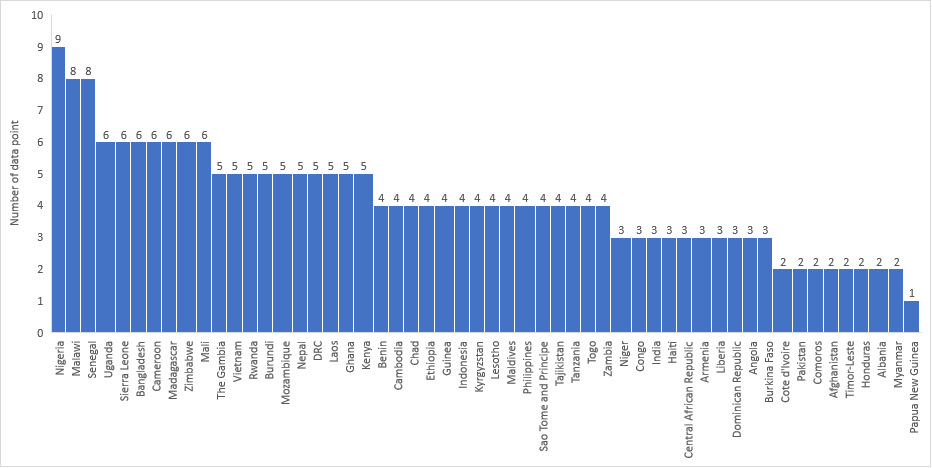


Figure 1: Number of data points by country

Table 1: Country specific data sources ^a^

| **Country** | **Survey name and year** |
| --- | --- |
| Afghanistan | DHS 2015; MICS 2010-11 |
| Albania | DHS 2009, 2018 |
| Angola | DHS 2007, 2011 |
| Armenia | DHS 2005, 2010, 2016 |
| Bangladesh | DHS 2004, 2007, 2011, 2014; MICS 2006, 2013 |
| Benin | DHS 2006, 2012, 2018; MICS 2014 |
| Burundi | DHS 2010, 2012, 2017; MICS 2000, 2005 |
| Burkina Faso | DHS 2003, 2010; MICS 2006 |
| Cambodia | DHS 2000, 2005, 2010, 2014 |
| Cameroon | DHS 2004, 2011; MICS 2000, 2006, 2014 |
| Central African Republic | MICS 2000, 2006, 2010 |
| Chad | DHS 2004, 2015; MICS 2000, 2010 |
| Comoros | DHS 2012; MICS 2000 |
| Congo | DHS 2005, 2012; MICS 2015 |
| Congo Democratic Republic | DHS 2007, 2014; MICS 2001, 2010, 2018 |
| Dominican Republic | DHS 2002, 2007, 2013 |
| Ethiopia | DHS 2000, 2005, 2011, 2016 |
| Cote d'Ivoire | MICS 2006, 2016 |
| Ghana | DHS 2003, 2008, 2014; MICS 2006, 2011 |
| Guinea | DHS 2005, 2012, 2018; MICS 2016 |
| Haiti | DHS 2006, 2012, 2016 |
| Honduras | DHS 2006, 2012 |
| India | DHS 2006, 2016; MICS 2000 |
| Indonesia | DHS 2003, 2007, 2012, 2017 |
| Kenya | DHS 2003, 2009, 2014; MICS 2000 |
| Kyrgyzstan | DHS 2012; MICS 2006, 2014, 2018 |
| Laos | MICS 2000, 2006, 2012, 2017 |
| Lesotho | DHS 2004, 2009, 2014; MICS 2014 |
| Liberia | DHS 2007, 2013, 2016 |
| Madagascar | DHS 2004, 2009; 2011, 2013, 2016; MICS 2000 |
| Malawi | DHS 2000, 2004, 2010, 2016; MICS 2006, 2014 |
| Maldives | DHS 2009, 2017 |
| Mali | DHS 2001, 2006, 2013, 2018; MICS 2010, 2015 |
| Mozambique | DHS 2003, 2011, 2015, 2018; MICS 2008 |
| Myanmar | DHS 2016; MICS 2010 |
| Nepal | DHS 2001, 2006, 2011, 2016; MICS 2014 |
| Niger | DHS 2006, 2012; MICS 2000 |
| Nigeria | DHS 2003, 2008, 2010, 2013, 2015, 2018; MICS 2007, 2011, 2017 |
| Pakistan | DHS 2013, 2018 |
| Papua New Guinea | DHS 2018 |
| Philippines | DHS 2003, 2008, 2013, 2017 |
| Rwanda | DHS 2000, 2005, 2008, 2010, 2015 |
| Sao Tome and Principe | DHS 2009; MICS 2000, 2006, 2014 |
| Senegal | DHS 2005, 2011, 2013, 2014, 2015, 2016, 2017; MICS 2000 |
| Sierra Leone | DHS 2008, 2013; MICS 2000, 2005, 2010, 2017 |
| Tajikistan | DHS 2012, 2017; MICS 2000, 2005 |
| Tanzania | DHS 2005, 2010, 2016, 2017 |
| The Gambia | DHS 2013; MICS 2000, 2006, 2010, 2018 |
| Timor-Leste | DHS 2010, 2016 |
| Togo | DHS 2014, 2017; MICS 2006, 2010 |
| Uganda | DHS 2001, 2006, 2009, 2011, 2015, 2016 |
| Vietnam | DHS 2002, MICS 2000, 2006, 2011, 2014 |
| Zambia | DHS 2002, 2007, 2014, 2018 |
| Zimbabwe | DHS 2006, 2011, 2015; MICS 2009, 2014, 2019 |

Note: a All information taken from survey reports. DHS does not formally include women in institutions, or otherwise living outside of households. The surveys may include internally displaced persons or homeless women if they stayed in a household the night before the interview, however, DHS has no information as to whether they are internally displaced or homeless. For the purposes of MICS, internally displaced people living in United Nations/government notified camps, military installations, and non-residential units such as business establishments were not considered in the scope of the survey.

DHS, Demographic and Health Survey; MICS, Multiple Indicator and Cluster Survey

**e-method 1: Definitions and calculation procedure of the coverage of INSD and SBA**

Consistent with previous studies, coverage of institutional delivery (INSD) was defined as the proportion of adolescent with a live birth who had their delivery in a health facility; and skilled birth attendant (SBA) was defined by the proportion of adolescent with a live birth who had their delivery assisted by a skilled health professional including doctor, nurse, midwife and other country-specific health cadres. The estimated coverage of institutional delivery (INSD) as the proportion of adolescent women. The coverage of INSD and SBA was calculated using the following formula:

$$Coverage of INSD=\frac{Adolescent who did INSD}{Total number of adolescent}$$

$$Coverage of SBA=\frac{Adolescent who did SBA}{Total number of adolescent}$$

For the other age group, same approaches were applied to estimate coverage. Since the proportion was calculated from individual level data, we accounted for probability sample design.

**e-method 2: Bayesian model**

Bayesian approach was used and favoured with the aim to project probabilities, which would not be possible using a frequentist approach. The essential difference between the two approaches is how probability is used. If we were to use a frequentist approach, the 95% CIs cannot calculate the probability of observing a future value, and hence we would be limited to using probability to *only* model certain processes (using process of “sampling”). For the purpose of our study, we were required to use probability more widely to model both sampling and other kinds of uncertainty which could only be conducted using Bayesian approach.

As a result of the advantage to produce probabilistic-oriented inferences, Bayesian methods are increasingly being applied to scientific fields, particularly in ecology where many cases outperform deterministic approaches. Since ecological modelling is characterized by high uncertainty due to the complex and often unknown cause-effect relationships among variables, a probabilistic approach is necessary to yield distributions of possible outcomes - in essence, transforming uncertainty into probability thresholds. This unique advantage of Bayesian approach over frequentist approach was key to conducting our study. Another advantage of using Bayesian methods in our study was the ability to combine prior knowledge about parameters with evidence from data. This method is favored for analysis of hierarchical models such as our study, which enables: flexibility in specifying hierarchical structures of parameters using priors; ability to handle small samples and model misspecification (overparameterization of the likelihood can be resolved with well-chosen priors); explicit handling of uncertainty; and intuitive and easy interpretation of results (credible interval versus confidence interval).

The following Bayesian hierarchical regression model was used to estimate the trends in, and projections of INSD and SBA indicators up to 2030 at country level:

$$y_{ijkl} \sim Normal(\hat{y}_{jkl},\tau^{2})$$

$$\hat{y}_{jkl}= \beta_{0, jk}+ \beta_{1, jk}{year}_{jk}+ \beta_{2,jk}{SDI}_{jk}+ \beta_{3, jkl}{HRH}_{jk}$$

$$\beta_{jk}\sim N(\beta_{j},\sigma_{J}^{2})$$

$$\beta_{j}\sim N(\beta,\sigma^{2})$$

$$\beta\sim Normal(0, 10000)$$

$$\sigma_{j},\sigma\sim Gamma(0.001, 0.001)$$

$$\tau\sim Uniform(0,1000)$$

where y is the logit-transformed probability of INSD and SBA prevalence from year $l$ in country k and region j. sociodemographic index (SDI) and human resources for health (HRH) were the year-specific ($l)$ predictor variables from country k in region j. $\beta_{jk}=[\beta_{0,jk}, \beta_{1, jk}{, \beta}_{2,jk,}{, \beta}_{3,jk}]$ is the country specific linear regression model parameter vector which includes intercept ($\beta_{0,jk}$) and the slopes for year ($\beta_{1, jk}$), SDI ($\beta_{2,jk,}$), and HRH ($\beta_{3,jk}$).$\tau^{2}$ is the model error variance, $\beta_{j}$=[$\beta_{0,j},\beta_{1,j},\beta_{2,j},\beta_{3,j}]$ is the vector of model parameter mean for country region j. $\sigma_{J}^{2}=[\sigma_{0,J}^{2}{, \sigma}_{1,J}^{2},\sigma_{2,J}^{2},\sigma_{3,J}^{2}]$ is the vector of variance of model parameters among countries belongings to region j, while $\beta=[\beta_{0}, \beta_{1}{, \beta}_{2,}{, \beta}_{3}]$ and $\sigma^{2}=[\sigma_{0}^{2}{,\sigma}_{1}^{2}, \sigma_{2}^{2}, \sigma_{3}^{2}]$ are the means and variance among regions, respectively. Non-informative prior distributions were assigned to $\beta,\tau,\sigma_{j}, and \sigma$ which represent the hyperparameters follow a normal distribution with mean 0 and variance 10,000; a uniform distribution with lower (0) and upper (1000) limits; and a gamma distribution with shape parameter k(0.001) and scale parameter ($\theta)$(0.0001), respectively. The hyperparameters of $\tau,\sigma_{j}, and \sigma$ $\tau, \sigma_{i}, and \sigma$are considered non-informative as there is no information about their distribution.

**e-method 3: Sensitivity analysis**

Both covariates and the model’s hierarchical structure influence how data from other countries influence predictions for a given country. We examined the sensitivity of our results by two approaches: (1) the exclusion of country-level covariates (SDI and HRH), and (2) altering priors for the hyperparameters.

*Sensitivity analysis: assessing the role of country-level covariates:*

Excluding country level predictors, the median absolute differences between the two sets of results and DIC values were compared.

*Altering priors for the hyperparameters:*

Previous studies reported that to borrow strength and facilitate parameters smoothening from each group, the hyperparameters were shared by all intercept coefficients. The key benefit of assigning the hyperparameter is the fact that the resulting model gains the advantages of a complete-pooled model and a no-pooled model. The half-Cauchy is quite heavy tailed and considered as fairly weakly informative. Gelman (2006) advocates for half-t priors (including the half-Cauchy) over the inverse gamma. The hyperparameter priors (hyper-priors) were assigned flat non-informative prior distributions for main analysis and weekly informative hyper-priors for sensitivity analysis. In our proposed model, $\beta_{ij}$ are conditionally normally distributed on $\beta_{k}, \sigma_{k}^{2}$ while $\beta_{k}$ is conditionally normally distributed on $\beta and \sigma^{2}.$ In the main analysis, $\beta=[\beta_{0}, \beta_{1}, \beta_{2}]$ is the normal distribution was specified in terms of mean zero and the standard deviation of 100, ${[\beta}_{0}, \beta_{1}, \beta_{2}]$~N(0.0.0001). Since the hyperparameter have some influences on all intercept coefficients, the half-Cauchy distribution (weekly informative prior) applied to perform sensitivity analysis instead of gamma distribution, hyperparameters (𝜏, 𝜎_k_, 𝑎𝑛𝑑 𝜎)~ half-Cauchy (0,25). After altering prior distribution, the median absolute differences between the two sets of results and DIC values were also compared.

**e-method 4: Bayesian model for determinant analysis**

For determinant analysis of institutional delivery and skill birth attendants, Bayesian multilevel analysis of random intercept model was used. The multilevel model account for dependency across hierarchy of data (i.e. individuals nested within country). In classical regression models assumed all individuals are independent that any predictors variables which affects the utilization of delivery care services has the same effect across all countries. In case of multilevel models are used to assess whether the of effect of predictors varies from country to country. The standard assumption is that Y_ij_ has a Bernoulli distribution. Similar to logistic regression model, the P_ij_ is modeled using the link fuction logit. The two-level model are as follows:

$$y_{ij} \sim Bern\left( p_{ij} \right)$$

$$logit(p_{ij})=\log\left[ \frac{p_{ij}}{1-p_{ij}} \right]=\beta_{0j}+\sum_{h=1}^{k} \beta_{hj}X_{hij}$$

$$\beta_{0j}=\beta_{0}+u_{0j}$$

where $p_{ij}$ is the probability of the binary outcome (institutional delivery or skilled birth attendant) for women i in country j. Where the intercept term $\beta_{0j}$ is assumed to vary randomly. The final model

$$logit\left( p_{ij} \right)=\beta_{0}+\sum_{h=1}^{k} \beta_{hj}X_{hij}+U_{0j}$$

Where $\beta_{0}+\sum_{h=1}^{k} \beta_{hj}X_{hij}$ is the fixed part of the model and $U_{0j}$is the random part of the model. The random intercept variance, var($U_{0j})=\sigma_{0}^{2}$. Normal prior for regression cofficients, $\beta\sim N(0, 10000)$ and inverse-gamma prior for the variance of random intercepts,$\sigma\sim inverse-gamma(0.01, 0.01)$.

Figure 2: Detail year-specific observed and predicted coverage of INSD by area of residence (national, urban, and rural)

Rural

Urban

National

Rural

Urban

National

Rural

Urban

National

Rural

Urban

National

Rural

Urban

National

Rural

Urban

National

Figure 3: Detail year-specific observed and predicted coverage of SBA by area of residence (national, urban, and rural)

Rural

Urban

National

Rural

Urban

National

Rural

Urban

National

Rural

Urban

National

Rural

Urban

National

Rural

Urban

National

Table 2: Percentage change in coverage of INSD and SBA visits between 2000 and 2030

| **Country** | Percentage change in INSD | | |  | Percentage change in SBA | | |
| --- | --- | --- | --- | --- | --- | --- | --- |
|  | **National** | **Urban** | **Rural** |  | **National** | **Urban** | **Rural** |
| **South Asia** |  |  |  |  |  |  |  |
| Afghanistan | 92.42 | 92.52 | 93.82 |  | 84.49 | 83.89 | 85.86 |
| Bangladesh | 91.19 | 91.38 | 92.96 |  | 89.4 | 89.49 | 91.58 |
| India | 78.51 | 82 | 87.67 |  | 69.81 | 73.97 | 81.49 |
| Maldives | 3.81 | 3.49 | 4.02 |  | 27 | 26.3 | 27.9 |
| Nepal | 90.15 | 93.15 | 96.34 |  | 87.96 | 91.75 | 95.53 |
| Pakistan | 76 | 76.9 | 80.65 |  | 68.95 | 70.52 | 75.72 |
| **East Asia and the Pacific** |  |  |  |  |  |  |  |
| Cambodia | 89.17 | 89.97 | 92.37 |  | 66.03 | 66.8 | 72.81 |
| Indonesia | 70.87 | 66.74 | 67.49 |  | 47.23 | 42.2 | 44.33 |
| Laos | 92.78 | 92.98 | 94.57 |  | 82.78 | 82.99 | 86.5 |
| Myanmar | 70.85 | 70.38 | 71.99 |  | 61.88 | 61.22 | 64.15 |
| Papua New Guinea | 65.6 | 68.66 | 73.39 |  | 61.61 | 63.82 | 68.61 |
| Philippines | 71.2 | 70.92 | 75.03 |  | 46.34 | 47.02 | 53.82 |
| Timor-Leste | 90.81 | 91.52 | 93.5 |  | 83.73 | 85.42 | 88.95 |
| Vietnam | 34.89 | 23.88 | 19.51 |  | 31.48 | 21.57 | 18.15 |
| **Eastern and Southern Africa** |  |  |  |  |  |  |  |
| Angola | 76.35 | 76.73 | 79.34 |  | 72.84 | 72.39 | 74.87 |
| Burundi | 80.51 | 82.2 | 86.09 |  | 74.9 | 76.39 | 80.87 |
| Comoros | 47.19 | 47.4 | 51.61 |  | 35.11 | 34.6 | 39.11 |
| Ethiopia | 94.34 | 95.04 | 96.23 |  | 88.58 | 89.44 | 91.42 |
| Kenya | 9.61 | 10.13 | 12.8 |  | 54.12 | 56.86 | 64.65 |
| Lesotho | 53.35 | 56.48 | 63.66 |  | 49.48 | 52.47 | 60.13 |
| Madagascar | 69.39 | 67.95 | 69.86 |  | 27.99 | 25.74 | 27.3 |
| Malawi | 50.61 | 54.86 | 63.48 |  | 50.15 | 53.41 | 61.46 |
| Mozambique | 43.56 | 47.6 | 55.56 |  | 41.18 | 45.38 | 53.44 |
| Rwanda | 71.24 | 78.44 | 86.32 |  | 62.49 | 70.68 | 80.65 |
| Tanzania | 50.59 | 53.61 | 60.21 |  | 51.23 | 54.23 | 60.91 |
| Uganda | 59.47 | 64.7 | 72.97 |  | 57.7 | 62.33 | 70.42 |
| Zambia | 60.46 | 65.28 | 73.59 |  | 59.68 | 63.66 | 71.88 |
| Zimbabwe | 52.33 | 53.02 | 58.96 |  | 50.95 | 51.16 | 56.85 |
| **West and Central Africa** |  |  |  |  |  |  |  |
| Benin | 17.19 | 17.42 | 20.68 |  | 8.32 | 8.15 | 9.34 |
| Burkina Faso | 67.18 | 68.67 | 73.82 |  | 45 | 44.89 | 49.73 |
| Cameroon | 20.03 | 19.92 | 22.58 |  | 21.6 | 20.29 | 22.3 |
| Central African Republic | 42.49 | 42.88 | 47.17 |  | 38.86 | 39.05 | 43.21 |
| Chad | 79.72 | 80.08 | 81.84 |  | 43.06 | 42.05 | 43.1 |
| Congo | 21.22 | 21.73 | 25.87 |  | 15.27 | 14.77 | 17.36 |
| Cote d'Ivoire | 50.67 | 51.01 | 55.18 |  | 50.98 | 50.71 | 54.88 |
| DRC | 33.85 | 33.04 | 36.11 |  | 28.84 | 28.34 | 31.5 |
| Ghana | 63.84 | 62.96 | 67.13 |  | 64.13 | 62.28 | 66.24 |
| Guinea | 69.35 | 70 | 73.76 |  | 61.97 | 62.61 | 66.93 |
| Liberia | 76.86 | 77.25 | 80.59 |  | 56.68 | 56.97 | 61.48 |
| Mali | 52.68 | 54.17 | 58.59 |  | 49.3 | 50.12 | 54.33 |
| Niger | 76.15 | 74.6 | 75.34 |  | 58.22 | 57.12 | 58.74 |
| Nigeria | 31.25 | 27.79 | 27.43 |  | 33.97 | 29.82 | 29.73 |
| Sao Tome and Principe | 35.36 | 39.63 | 48.21 |  | 20.36 | 26.07 | 38.53 |
| Senegal | 38.41 | 38.52 | 42.68 |  | 20.35 | 19.88 | 21.81 |
| Sierra Leone | 92.47 | 92.57 | 93.77 |  | 59.89 | 59.89 | 64.82 |
| The Gambia | 66.07 | 66.38 | 69.87 |  | 62.87 | 63.57 | 67.74 |
| Togo | 10.62 | 11.14 | 13.64 |  | 13.65 | 14.24 | 17.26 |
| **Latin America and Caribbean** |  |  |  |  |  |  |  |
| Dominican Republic | 4.6 | 4.4 | 4.8 |  | 1.91 | 1.81 | 1.81 |
| Haiti | 67.01 | 68.43 | 72.74 |  | 66.48 | 67.52 | 72.06 |
| Honduras | 39.75 | 43.11 | 51.3 |  | 35.78 | 38.42 | 46.2 |
| **Central and Eastern Europe** |  |  |  |  |  |  |  |
| Albania | 4.91 | 5.22 | 6.43 |  | 1.4 | 1.4 | 1.5 |
| Armenia | 1.3 | 1.5 | 1.9 |  | 0.9 | 1 | 1.2 |
| Kyrgyzstan | 3.2 | 4.2 | 6.31 |  | 1.2 | 1.8 | 3.1 |
| Tajikistan | 37.42 | 46.44 | 59.89 |  | 13.79 | 18.3 | 27.36 |

Table 3: Coverage of INSD among adult women aged 20-49 years in 54 low- and middle-income countries, 2000-2030

| Country | Predicted coverage (95 % CrI) | | | | | |
| --- | --- | --- | --- | --- | --- | --- |
|  | 20-35 years | | | 36-49 years | | |
|  | 2000 | 2018 | 2030 | 2000 | 2018 | 2030 |
| Afghanistan | 7 (1.4-19.3) | 65.2 (51.4-77.9) | 93.6 (78.3-99.3) | 5.7 (1.1-15.6) | 60.1 (45.2-73.8) | 92.2 (73.4-99) |
| Bangladesh | 7.3 (4.9-10.6) | 50.6 (40.7-61.1) | 84.7 (71.5-93.5) | 5.7 (3.6-8.1) | 43.9 (34.1-53.6) | 81 (64.9-91.4) |
| India | 17.6 (10-28) | 84.4 (77.5-89.7) | 97.8 (94.8-99.2) | 11.9 (6.2-19.9) | 77.3 (66.7-85.3) | 96.5 (91.9-98.8) |
| Maldives | 94 (87.6-97.8) | 96.7 (95-97.8) | 97.4 (92.8-99.2) | 93.1 (86-97.6) | 96.2 (94-97.8) | 97 (91.9-99.2) |
| Nepal | 6.6 (4.6-9.1) | 70.8 (63-77.4) | 96.3 (93.2-98.3) | 3.4 (2.4-5) | 55.1 (45.2-64.7) | 92.9 (86.6-96.7) |
| Pakistan | 20.7 (5.3-48) | 68.7 (60.6-75.9) | 89.6 (73-97.4) | 16.8 (3.9-40.9) | 62.3 (51.9-72.4) | 86.8 (67.7-96.7) |
| Cambodia | 10 (7.3-13.5) | 93.5 (90.3-95.7) | 99.7 (99.4-99.9) | 7.6 (5.4-10.5) | 91.3 (87-94.5) | 99.6 (99.1-99.8) |
| Indonesia | 31.1 (23-40.6) | 78.5 (71.6-84.3) | 93.5 (87.7-97) | 30.3 (21.8-40.5) | 77.8 (69.2-84.5) | 93.2 (86.6-97) |
| Laos | 6.6 (3.6-11.1) | 64.8 (56-73.5) | 94 (87.5-97.8) | 5 (2.6-9.3) | 57.9 (46.7-68.4) | 92.1 (84.1-96.9) |
| Myanmar | 21.3 (2.1-64.2) | 50.6 (37-63.1) | 71.9 (30.9-95.7) | 19.3 (1.7-63.7) | 46.4 (31.1-61.2) | 68.9 (26.2-95) |
| Papua New Guinea | 24.6 (2.5-70.8) | 57.4 (47.6-67.1) | 78.5 (46.8-95.3) | 19 (1.4-63.2) | 47 (32.2-62) | 71.4 (36.3-93.2) |
| Philippines | 27.8 (19.5-36.7) | 81.6 (75.8-86.4) | 95.6 (91.9-98) | 23.6 (15.7-33.4) | 77.9 (70-84.5) | 94.5 (89.5-97.6) |
| Timor-Leste | 7.6 (2.2-17.2) | 57.7 (46.5-69.4) | 89.6 (73.9-97.9) | 5.6 (1.6-13.3) | 49.8 (35.3-64.5) | 86.2 (65.3-97.1) |
| Vietnam | 74.6 (66.3-82.2) | 94.3 (91.3-96.4) | 98 (95.5-99.3) | 79.2 (71.4-86.2) | 95.5 (92.8-97.3) | 98.4 (96.4-99.5) |
| Angola | 18.5 (0.9-68.1) | 55.6 (42.6-68.4) | 79.5 (31.2-98.6) | 15.7 (0.6-64.3) | 49.6 (36.3-62.7) | 76 (26-98.1) |
| Burundi | 17.6 (10.5-26.3) | 89 (84.5-92.7) | 98.9 (97.5-99.6) | 13.7 (7.8-21.1) | 85.7 (79.8-90.5) | 98.5 (96.6-99.5) |
| Comoros | 49.6 (15.1-83.6) | 87.6 (75.6-95) | 94.3 (74.1-99.7) | 45.1 (12-81.3) | 85.2 (71.1-94) | 93.2 (71-99.6) |
| Ethiopia | 3.7 (2.6-5.1) | 34.3 (25.7-43.5) | 74.6 (58.6-86.4) | 2.5 (1.7-3.5) | 25.8 (18.2-35.2) | 66.3 (47.3-81.7) |
| Kenya | 55 (46.5-62.2) | 58.8 (46.7-70.8) | 61.2 (37-83.7) | 47 (37.6-56.2) | 51 (38.1-64.3) | 53.9 (28.6-79.2) |
| Lesotho | 41.3 (29.6-54.2) | 83.8 (75.7-90.4) | 94.9 (87.6-98.5) | 33.8 (22.6-47.5) | 78.8 (68.8-87.3) | 93 (83.8-98) |
| Madagascar | 24.1 (13.9-37.2) | 55.9 (31.8-78.1) | 75.2 (35.4-96.4) | 22 (11.8-34.9) | 53 (27.3-76.2) | 73 (29.6-96.2) |
| Malawi | 44.6 (37.8-52.4) | 92.9 (90.5-95) | 98.8 (97.8-99.4) | 35.9 (28.7-44.3) | 90.1 (86.3-93.1) | 98.3 (96.9-99.2) |
| Mozambique | 44.8 (34-56.1) | 73.3 (63.5-81.1) | 85.5 (70.7-94) | 35.6 (24.4-47.4) | 65 (53.1-75.3) | 80.1 (60.7-92) |
| Rwanda | 21.5 (16.3-27.5) | 94.8 (92.4-96.6) | 99.7 (99.3-99.9) | 13.6 (9.7-18.6) | 91.2 (87.1-94.4) | 99.4 (98.7-99.8) |
| Tanzania | 38.6 (26.3-51.3) | 68.9 (58.9-77.3) | 83.2 (67-93.3) | 31 (19.9-44.6) | 61.1 (48.5-72.3) | 77.9 (57.5-91) |
| Uganda | 32.9 (24.7-41.1) | 78.6 (70.5-84.6) | 93.2 (86.3-97.1) | 24.3 (16.8-32.7) | 70.5 (59.1-79.7) | 89.9 (80-95.6) |
| Zambia | 33.3 (24.6-42.1) | 83.5 (78.5-87.8) | 95.9 (92.7-98) | 24.8 (17.4-33.3) | 76.9 (69.3-83.4) | 93.9 (89-97) |
| Zimbabwe | 44.3 (30-58.5) | 83.3 (77.5-88.3) | 94.3 (87.7-98.1) | 38 (24.8-52.7) | 79.2 (71.4-85.8) | 92.6 (83.9-97.7) |
| Benin | 76.8 (65-85.7) | 88.5 (85.2-91.5) | 93 (85.5-97.5) | 72.5 (59.5-83.3) | 86 (81.6-89.8) | 91.4 (82.2-96.9) |
| Burkina Faso | 30.7 (21-40.8) | 89.2 (80-95.1) | 98 (93.4-99.7) | 25.5 (16.9-35.2) | 86.4 (75.3-94) | 97.4 (91.7-99.6) |
| Cameroon | 57.9 (43.2-69.4) | 66.9 (55.8-76.9) | 72.3 (50.1-89) | 52.8 (38-65.8) | 62.2 (50.1-73.7) | 68.2 (44.6-87.3) |
| Central African Republic | 41.7 (23.5-62.1) | 62.9 (39.3-82.9) | 73 (27.9-96.5) | 36.4 (18.8-56.6) | 57.6 (33.7-79.3) | 68.9 (24.1-95.8) |
| Chad | 9.9 (5.8-15.3) | 28.9 (19.8-39.1) | 49.7 (25.9-73.3) | 8.1 (4.5-13.1) | 24.7 (16-35.5) | 44.6 (20.4-70.3) |
| Congo | 76.7 (64.4-85.7) | 94.5 (91.6-96.6) | 98 (94.8-99.4) | 72.2 (58-83.3) | 93.1 (89.5-95.6) | 97.4 (93.4-99.2) |
| Cote d'Ivoire | 43.5 (28.8-59.3) | 76.3 (67.2-84.2) | 88.8 (76-96) | 38.9 (23.9-55.1) | 72.6 (61.7-81.4) | 86.8 (71.2-95.4) |
| Democratic Republic of the Congo | 61.2 (46.7-74) | 83.3 (78.4-87.6) | 91.4 (83.3-96.4) | 57.5 (42.5-70.9) | 81 (74.3-86) | 90 (80.1-95.9) |
| Ghana | 35 (26.4-44.8) | 81.7 (73.8-87.9) | 94.5 (87.9-98.1) | 30.7 (22-41.1) | 78.5 (69.2-85.9) | 93.4 (85.1-97.5) |
| Guinea | 23.7 (14.9-34) | 58.2 (50.3-65.4) | 79 (65.2-88.5) | 19.6 (11.8-29.6) | 52.1 (43-61.5) | 74.7 (58.3-86.7) |
| Liberia | 21.7 (11.3-35.3) | 79 (71.5-85.3) | 95.4 (89.7-98.5) | 18.3 (9-31.6) | 75 (65.2-83.1) | 94.3 (86.9-98.1) |
| Mali | 39 (30-48.9) | 70.1 (63.8-75.9) | 85.1 (75.7-91.4) | 34 (25-44) | 65.4 (57.1-72.8) | 82.1 (71.1-90) |
| Niger | 17.3 (12.8-23.4) | 45.9 (31.9-60.3) | 68.1 (42.9-87.1) | 16.5 (11.5-23.2) | 44.6 (29.5-60.6) | 66.9 (40.3-86.9) |
| Nigeria | 33 (25.2-41.9) | 40.2 (33.4-47) | 45.7 (31.2-60) | 33.6 (25.2-42.8) | 40.8 (33.3-49) | 46.3 (31.2-60.9) |
| Sao Tome and Principe | 59.4 (33.8-80.6) | 94.2 (90.1-97.1) | 98.4 (94.7-99.8) | 50.6 (24.1-75.1) | 91.6 (85.2-96) | 97.7 (92.2-99.7) |
| Senegal | 55.7 (44-67.5) | 81.3 (77-84.9) | 90.6 (83.5-95.1) | 50.9 (37.9-64.9) | 78.1 (72.6-82.9) | 88.8 (80.6-94.3) |
| Sierra Leone | 7.3 (4.4-10.8) | 80.8 (74.7-86.2) | 98.3 (96.5-99.3) | 6.1 (3.5-9.7) | 77.3 (69.7-83.9) | 97.9 (95.6-99.2) |
| The Gambia | 31.8 (21-44.3) | 80.6 (74.6-85.9) | 94.6 (89.4-97.8) | 28.2 (17.4-41) | 77.7 (69.8-84.3) | 93.6 (87.1-97.3) |
| Togo | 67.8 (51.5-80.9) | 73.9 (62.2-83.8) | 76.3 (48.9-93.2) | 61.4 (44.2-76.3) | 68.1 (53.8-80.2) | 71.1 (42-91.4) |
| Dominican Republic | 95.5 (90.8-98.3) | 99.6 (99.2-99.8) | 99.9 (99.7-100) | 95.1 (89.8-98) | 99.5 (99.1-99.8) | 99.9 (99.7-100) |
| Haiti | 20.9 (11.7-32.9) | 45.6 (35.7-56) | 66.2 (43.9-84.2) | 16 (8.6-27.2) | 37.6 (27.1-48.9) | 58.7 (33.5-79.6) |
| Honduras | 55.3 (36.7-71.7) | 91.2 (84-95.7) | 97.2 (90.6-99.5) | 46.8 (28.3-65.7) | 87.9 (77.8-94.2) | 96.1 (86.7-99.4) |
| Albania | 94.5 (89.5-97.6) | 99 (98.5-99.3) | 99.7 (99.3-99.9) | 93.2 (87.7-97.1) | 98.7 (98.2-99.2) | 99.6 (99-99.9) |
| Armenia | 98.5 (97.4-99.2) | 99.9 (99.9-100) | 100 (100-100) | 98.1 (96.8-99) | 99.9 (99.9-100) | 100 (100-100) |
| Kyrgyzstan | 95.7 (92.7-97.7) | 99.7 (99.5-99.7) | 99.9 (99.9-100) | 93.6 (89.1-96.6) | 99.5 (99.2-99.6) | 99.9 (99.8-100) |
| Tajikistan | 51.9 (38-65.1) | 88.9 (84.5-92.3) | 96.9 (93.4-98.8) | 37.9 (24-52.5) | 81.7 (74.4-87.7) | 94.5 (88.8-97.9) |

Table 4: Coverage of SBA among adult women aged 20-49 years in 54 low- and middle-income countries, 2000-2030

| country |  |  | Predicted coverage (95 % CrI) |  |  |  |  |  |
| --- | --- | --- | --- | --- | --- | --- | --- | --- |
|  |  | 20-35 years | | |  | 36-49 years | | |
|  |  | **2000** | **2018** | **2030** |  | **2000** | **2018** | **2030** |
| Afghanistan |  | 14.5 (3.8-34.6) | 64.3 (50.7-75.8) | 90 (67.2-98.3) |  | 12.5 (3.1-30.8) | 60.2 (46-72.8) | 88.4 (63-98) |
| Bangladesh |  | 9.3 (6.2-13.4) | 57.5 (46.7-67.6) | 88.5 (76.4-95.4) |  | 7.2 (4.8-10.8) | 50.9 (39.2-61.9) | 85.5 (70.4-94.1) |
| India |  | 25.3 (14.2-39.9) | 85.1 (77.6-90.6) | 97.2 (92.8-99.2) |  | 17.7 (9-30.4) | 78.1 (66.9-86.8) | 95.6 (88.4-98.8) |
| Maldives |  | 73.7 (54-88.6) | 99.8 (99.7-99.9) | 100 (100-100) |  | 72.1 (51.3-88.4) | 99.8 (99.7-99.9) | 100 (100-100) |
| Nepal |  | 7.9 (5.4-11.1) | 70.4 (62.6-78.1) | 95.8 (91.6-98.2) |  | 4.1 (2.6-6.1) | 54.1 (42.8-64.2) | 91.8 (83.9-96.3) |
| Pakistan |  | 26.5 (8-53.6) | 71.7 (63.2-78.9) | 89.9 (74.8-97.3) |  | 21 (5.7-47.4) | 64.5 (53.3-74.8) | 86.5 (66.9-96.5) |
| Cambodia |  | 33 (25.6-41.1) | 94.4 (91.5-96.5) | 99.4 (98.7-99.8) |  | 27 (19.5-35.1) | 92.7 (88.4-95.6) | 99.3 (98.3-99.7) |
| Indonesia |  | 56.7 (45.9-66.9) | 92.2 (89.2-94.6) | 98.1 (96-99.2) |  | 54.5 (42.9-65.3) | 91.5 (87.7-94.4) | 97.9 (95.3-99.1) |
| Laos |  | 14.9 (10.7-20.4) | 61.5 (52.4-71.1) | 87.6 (77.7-94.3) |  | 11.3 (7.6-15.6) | 53.7 (42-64) | 83.7 (69.8-92.5) |
| Myanmar |  | 33.7 (5-78.9) | 72.2 (60.9-81.5) | 86.9 (56.8-98.3) |  | 30.4 (4-74.9) | 68.1 (53.1-80.7) | 84.8 (53.5-98) |
| Papua New Guinea |  | 28.4 (3.7-71.3) | 58.9 (48.2-68.7) | 78.5 (51.3-94.5) |  | 22.6 (2.5-66.7) | 49.8 (35.1-64.6) | 72 (42.4-92.3) |
| Philippines |  | 50.6 (38.7-61.9) | 86.7 (80.9-91) | 95.5 (90.6-98.3) |  | 43.5 (30.9-56.9) | 82.9 (76.1-88.8) | 94.2 (87.8-97.8) |
| Timor-Leste |  | 13.3 (4.1-28.5) | 66.1 (54.3-77.4) | 91.2 (73.8-98.5) |  | 9.7 (2.8-21.9) | 57.3 (42.2-72) | 87.8 (65.6-97.7) |
| Vietnam |  | 77.1 (71.2-82.2) | 95 (92.6-96.8) | 98.3 (96.4-99.3) |  | 80.7 (74.3-86.2) | 95.9 (93.8-97.5) | 98.6 (97.2-99.5) |
| Angola |  | 22.2 (2.3-63.7) | 59 (45.9-70.8) | 80.4 (40.6-97.5) |  | 19.5 (2-60.7) | 54.2 (40.6-66.3) | 77.6 (35.8-97.1) |
| Burundi |  | 23.3 (17.1-29.8) | 89 (84.3-92.5) | 98.7 (97.3-99.4) |  | 18.8 (13.7-24.7) | 86 (80.2-90.4) | 98.3 (96.5-99.2) |
| Comoros |  | 63.7 (53.9-72.7) | 91.8 (85.9-95.6) | 97.4 (93.2-99.3) |  | 59 (47-69.8) | 90.1 (83.2-94.9) | 96.9 (91.8-99.1) |
| Ethiopia |  | 6.4 (4.6-8.7) | 30.5 (22.6-39.2) | 60.6 (42.4-77) |  | 4.4 (3-6.3) | 23 (16.2-31.1) | 51.3 (33-69.6) |
| Kenya |  | 37.1 (29.2-45.6) | 70.6 (59.1-80.3) | 86 (68-95.6) |  | 28.6 (20.9-37.4) | 62.1 (48.2-74.6) | 80.9 (58.4-93.9) |
| Lesotho |  | 45.2 (32.2-59) | 84.8 (77.1-90.6) | 95.1 (87.4-98.5) |  | 37.2 (24.5-51.2) | 79.9 (69.4-88.2) | 93.3 (83.1-98.1) |
| Madagascar |  | 47.6 (38.5-56.9) | 54.8 (36.3-71.5) | 64.1 (31.4-87.7) |  | 45 (34-56.8) | 52.3 (33.5-70.3) | 61.9 (29.5-86.1) |
| Malawi |  | 45.7 (37.6-54.3) | 91.1 (87.8-93.7) | 98.1 (96.5-99.1) |  | 37.5 (29.5-46.4) | 87.9 (83.5-91.6) | 97.3 (95-98.7) |
| Mozambique |  | 44.9 (33.4-56.4) | 70.1 (59.7-78.6) | 82.2 (65.5-92.8) |  | 35.2 (24.4-46.8) | 60.9 (48.3-72.6) | 75.6 (54.2-90.1) |
| Rwanda |  | 29.2 (22.3-36.3) | 94.5 (91.9-96.4) | 99.6 (98.9-99.8) |  | 19.2 (13.7-25.5) | 90.8 (86.1-94.3) | 99.2 (98.1-99.8) |
| Tanzania |  | 38.4 (25.1-52.3) | 69.4 (58.7-78.6) | 83.9 (68.3-94) |  | 30.8 (18.8-45.1) | 61.7 (48.1-73.8) | 78.8 (58.3-92.1) |
| Uganda |  | 35 (27.3-44.1) | 78.4 (70.7-84.7) | 92.9 (86-96.9) |  | 26.5 (18.9-35.8) | 70.8 (60.6-79.6) | 89.6 (80.3-95.6) |
| Zambia |  | 34.2 (25.4-42.8) | 80.1 (74.4-85.3) | 94.1 (89.4-97.2) |  | 25.7 (17.6-34.6) | 72.7 (63.9-80.3) | 91.4 (84.5-95.9) |
| Zimbabwe |  | 46.3 (31.9-61.1) | 84.1 (77.4-89.1) | 94.8 (88-98.4) |  | 40.3 (26.2-55.6) | 80.4 (71.8-86.7) | 93.4 (84.6-97.8) |
| Benin |  | 78.9 (67.3-87.6) | 82.3 (76.8-86.8) | 85.9 (70.3-94.7) |  | 75.7 (61.5-85.5) | 79.4 (72.6-84.8) | 83.5 (66.3-93.8) |
| Burkina Faso |  | 51.8 (38.8-65.1) | 85.3 (74.2-93.3) | 94 (81.9-98.9) |  | 46.6 (33.8-60) | 82.5 (69-92) | 92.7 (79.3-98.7) |
| Cameroon |  | 61.3 (53.4-69.4) | 70.5 (59.5-79) | 76.9 (58.6-88.7) |  | 57.5 (48.3-66.6) | 67.1 (55.2-76.7) | 74 (54.5-87.6) |
| Central African Republic |  | 44.8 (36-54.9) | 62.9 (45.3-77.8) | 73.5 (44.4-91.4) |  | 39.3 (29.7-49.9) | 57.5 (39-73.2) | 69.2 (38.6-89.3) |
| Chad |  | 21.5 (15.7-27.9) | 29.8 (21.3-39.5) | 37.1 (19.6-56.6) |  | 19.8 (14.1-25.8) | 27.7 (18.7-37.4) | 34.8 (17.3-53.9) |
| Congo |  | 83.7 (74.4-90.6) | 95.6 (93.4-97.3) | 98.2 (95.6-99.5) |  | 80.9 (69.8-89) | 94.7 (91.9-96.8) | 97.9 (94.7-99.4) |
| Cote d'Ivoire |  | 45.2 (29.9-61.4) | 80 (71.3-86.7) | 91.7 (81.2-97.3) |  | 40.7 (25.4-58.2) | 76.7 (66.5-85) | 90.2 (77.3-96.9) |
| Democratic Republic of the Congo |  | 66 (56.1-74.3) | 84.7 (80.1-88.6) | 92.1 (85.3-96.2) |  | 62.2 (51.9-71.7) | 82.4 (76.4-87.5) | 90.8 (82.8-95.8) |
| Ghana |  | 35.8 (26.4-46.1) | 82.4 (74.5-88.5) | 94.9 (88.1-98.2) |  | 31.7 (22.8-42.2) | 79.6 (69.4-87.2) | 93.9 (85.7-97.9) |
| Guinea |  | 29.8 (20.2-41.3) | 61.6 (54.2-68.5) | 79.7 (67-89.3) |  | 24.9 (15.6-37.2) | 55.4 (45.6-64.5) | 75.3 (60.1-86.9) |
| Liberia |  | 38.3 (19.4-59.8) | 75.3 (60.5-86.1) | 89 (67-98.1) |  | 33.4 (15.9-55.7) | 70.9 (53.1-84.4) | 86.7 (60.2-97.6) |
| Mali |  | 42.3 (32.8-52) | 70.3 (63.8-76) | 84.8 (75.3-91.8) |  | 37.4 (27.6-48) | 65.7 (57.4-73.4) | 81.9 (70.4-90.2) |
| Niger |  | 26.8 (20.1-34.7) | 47.4 (32.7-62.7) | 62.5 (37.1-84.2) |  | 24.8 (16.8-34) | 44.8 (29.2-61.6) | 60.1 (33.1-83) |
| Nigeria |  | 36 (27.4-45.4) | 44.6 (37.4-51.7) | 51.3 (34.8-66.4) |  | 36.4 (27.7-46.7) | 45.2 (36.5-53.4) | 51.8 (35.1-67.3) |
| Sao Tome and Principe |  | 72.6 (64.3-80) | 94.2 (91-96.4) | 98.2 (96.1-99.4) |  | 59.5 (46.8-69.9) | 89.9 (83.9-94.1) | 96.8 (92.6-98.9) |
| Senegal |  | 55.2 (46-63.1) | 63.6 (57.4-69) | 68.9 (56.2-78.7) |  | 50.9 (41.3-59.8) | 59.5 (52.7-66.2) | 65.1 (51.9-76.8) |
| Sierra Leone |  | 36.9 (29.2-44.9) | 77.4 (71.6-83) | 92 (86.5-95.8) |  | 31.7 (23.9-40.6) | 73 (64.8-80.3) | 90.1 (83-95) |
| The Gambia |  | 34.5 (22.5-48.6) | 81.3 (75.3-86.4) | 94.7 (89.7-97.9) |  | 30.2 (18.2-44.1) | 78 (70.3-84.8) | 93.6 (87.2-97.6) |
| Togo |  | 56.6 (39.7-71.6) | 62.3 (48.8-74.8) | 66 (38.1-88.1) |  | 48.9 (32.4-65.9) | 54.7 (39.4-69.9) | 59.1 (30.4-84.8) |
| Dominican Republic |  | 97.9 (95.8-99.2) | 99.3 (98.7-99.7) | 99.7 (98.9-99.9) |  | 97.9 (95.2-99.2) | 99.3 (98.6-99.7) | 99.7 (98.8-99.9) |
| Haiti |  | 22.9 (12.8-36.7) | 48.4 (37-59.4) | 70.5 (46.6-88.2) |  | 17.8 (9.1-29.9) | 40.5 (29.9-52.6) | 63.7 (38.9-85) |
| Honduras |  | 59.3 (42.2-75.5) | 90 (81.7-95) | 96.3 (87.3-99.3) |  | 51 (32.2-68.9) | 86.4 (74.1-93.3) | 94.8 (82.7-99.1) |
| Albania |  | 98.6 (97.1-99.5) | 99.9 (99.8-99.9) | 100 (99.9-100) |  | 98.5 (96.9-99.4) | 99.8 (99.8-99.9) | 100 (99.9-100) |
| Armenia |  | 99 (98.4-99.5) | 100 (99.9-100) | 100 (100-100) |  | 98.8 (98.1-99.4) | 99.9 (99.9-100) | 100 (100-100) |
| Kyrgyzstan |  | 98.1 (96.7-99.1) | 99.7 (99.6-99.8) | 99.9 (99.8-100) |  | 96.8 (94.5-98.4) | 99.5 (99.2-99.6) | 99.9 (99.7-99.9) |
| Tajikistan |  | 76.8 (70.6-82.5) | 88.9 (84.7-92.2) | 94 (88.3-97.2) |  | 65.3 (56-73.7) | 81.9 (74.9-87.6) | 89.9 (81-95.3) |

Table 5: Coverage of INSD among adolescents according to area of residence in 54 low- and middle-income countries, 2000-2030

| country | Urban (predicted coverage (95% CrI) | | |  | Rural (predicted coverage (95% CrI) | | |
| --- | --- | --- | --- | --- | --- | --- | --- |
|  | **uy2000** | **uy2018** | **uy2030** |  | **ry2000** | **ry2018** | **ry2030** |
| **South Asia** |  |  |  |  |  |  |  |
| Afghanistan | 18.5 (8.5-33.3) | 88.6 (79.3-94.3) | 98.7 (95.8-99.8) |  | 3.5 (1.5-7.2) | 55.8 (39.4-69.8) | 92.8 (81-98.4) |
| Bangladesh | 14.5 (8.5-23.7) | 67.7 (52.4-79.9) | 91.4 (77.8-97.7) |  | 5.9 (3.4-9.8) | 44.3 (30.5-58.6) | 80.6 (58.3-93.7) |
| India | 51.8 (36.4-67.6) | 93.2 (87.7-96.9) | 98.6 (96.1-99.7) |  | 24.6 (14.5-36.3) | 80.6 (68.5-89.8) | 95.5 (88-98.8) |
| Maldives | 80.9 (56.9-94) | 99.4 (98.8-99.7) | 99.9 (99.8-100) |  | 59.1 (30.2-83.8) | 98.1 (96.4-99.1) | 99.8 (99.3-100) |
| Nepal | 31.3 (19.9-45) | 87.6 (80.9-92.5) | 97.7 (94.6-99.3) |  | 10.9 (6.3-17.1) | 65.6 (52.7-77) | 92 (82.2-97.2) |
| Pakistan | 36.1 (16.2-60) | 78.4 (67.2-86.9) | 92 (80.1-97.9) |  | 19.2 (7.2-38.2) | 59.7 (45.4-73.8) | 82.7 (61.5-94.8) |
| **East Asia and the Pacific** |  |  |  |  |  |  |  |
| Cambodia | 31.5 (20.6-43.5) | 97.9 (96-99) | 99.9 (99.7-100) |  | 9.4 (6-14.4) | 91.3 (84.8-95.5) | 99.5 (98.7-99.9) |
| Indonesia | 46.3 (31.3-62.6) | 85.9 (78.1-91.9) | 95.4 (89.1-98.6) |  | 17.6 (9.7-28.3) | 59.9 (45.7-73.1) | 83.9 (66.2-94.1) |
| Laos | 21.8 (10-40.6) | 81.9 (71.8-89.7) | 96.5 (90.8-99.1) |  | 6.4 (2.7-13.1) | 52.7 (36.4-67.6) | 87.1 (67.7-96.4) |
| Myanmar | 47.6 (17.5-80.5) | 71.5 (55-85.9) | 80.5 (45.1-97.2) |  | 15.8 (3.2-41.5) | 31.9 (17-49) | 48.3 (13.4-84.5) |
| Papua New Guinea | 66.9 (14.7-97) | 92.1 (85.1-96.4) | 96.3 (86.1-99.6) |  | 27.9 (2-76.7) | 58.5 (39.7-75.9) | 77.9 (41-96.4) |
| Philippines | 33.9 (21.1-48.7) | 87.6 (80.6-92.6) | 97.4 (93.8-99.2) |  | 15.6 (8.6-24.8) | 71.7 (59.1-82.4) | 93.2 (84.4-97.8) |
| Timor-Leste | 20.8 (5.1-47) | 81.1 (67.6-90.2) | 95.9 (84.2-99.5) |  | 4.9 (1-15) | 44.7 (26.5-64.2) | 82.5 (49.1-97.3) |
| Vietnam | 99.6 (99.4-99.8) | 99.7 (99.4-99.9) | 99.7 (99.1-99.9) |  | 73.8 (60.3-84.2) | 77.5 (64.4-88.2) | 78.4 (52.6-94.2) |
| **Eastern and Southern Africa** |  |  |  |  |  |  |  |
| Angola | 60.6 (44-75.9) | 74.4 (59.3-86.5) | 79.4 (54-94.7) |  | 23 (13.1-36.5) | 36.1 (23-52.3) | 45.5 (18.8-76.3) |
| Burundi | 52.3 (33.1-71.4) | 97 (94.5-98.5) | 99.7 (99-99.9) |  | 21.6 (10.8-35.4) | 88.8 (81.5-93.7) | 98.6 (95.7-99.7) |
| Comoros | 64.8 (24.2-91.8) | 91.7 (79.9-97.7) | 95.4 (78.3-99.8) |  | 41.4 (8.9-80.2) | 79.2 (60-92.3) | 89 (54.9-99.6) |
| Ethiopia | 25.9 (16.2-37.5) | 85.9 (76.8-92.2) | 97.5 (93.6-99.2) |  | 2.2 (1.2-3.4) | 28.5 (17.9-41.2) | 72.5 (49.7-88.5) |
| Kenya | 60.2 (46.5-72.2) | 76.9 (62.9-87.7) | 82.9 (58.3-96.1) |  | 43.9 (31.5-56.2) | 63.5 (45.4-78.7) | 72.4 (38.5-92.7) |
| Lesotho | 76.9 (62.8-87.9) | 93.9 (88.5-97.3) | 97.4 (91.6-99.5) |  | 45.8 (30.2-63.4) | 79.3 (64.6-89.6) | 90.5 (72.4-98.1) |
| Madagascar | 34.8 (18.3-54.7) | 74.9 (48-92.1) | 88.6 (55.7-99.1) |  | 15.5 (6.5-28.3) | 52 (24.3-79.4) | 75.5 (30.2-97.4) |
| Malawi | 71.2 (60.2-80.1) | 98.3 (97.3-99) | 99.8 (99.5-99.9) |  | 41.8 (30.6-53.2) | 94.3 (91-96.6) | 99.2 (98.2-99.7) |
| Mozambique | 78.5 (65.5-88.1) | 92 (85.7-95.9) | 95.6 (88.3-98.9) |  | 40.3 (26.1-55.9) | 67.6 (52.3-80.3) | 80.4 (57.8-93.7) |
| Rwanda | 51.5 (38.2-64.1) | 97.6 (95.7-98.8) | 99.8 (99.4-99.9) |  | 31.2 (20.2-42.2) | 94.4 (90.7-97.2) | 99.5 (98.4-99.9) |
| Tanzania | 73.3 (55.7-86.7) | 89.5 (81.6-94.6) | 94.1 (84-98.6) |  | 36.6 (20-55) | 63.5 (45.8-79.1) | 77.3 (50.2-94) |
| Uganda | 70 (56.7-80.9) | 91.2 (85.7-95.1) | 96.4 (91.6-98.8) |  | 40.2 (27.5-53.7) | 74.8 (62-85.6) | 88.5 (74.7-96.3) |
| Zambia | 65.9 (51.1-78.5) | 94.6 (91-97) | 98.7 (96.9-99.6) |  | 29.8 (17.5-43.3) | 79.1 (68.5-86.8) | 94 (86.8-97.8) |
| Zimbabwe | 68.2 (49.4-83.1) | 90.1 (84.2-94.4) | 95.6 (88.5-98.9) |  | 44.3 (26.2-62) | 76.7 (63.9-86.2) | 88.8 (71.6-97) |
| **West and Central Africa** |  |  |  |  |  |  |  |
| Benin | 82.4 (66.6-91.7) | 93.9 (90.4-96.6) | 96.7 (90.9-99.2) |  | 65.5 (46-81.5) | 86 (80-90.9) | 92.2 (80.7-98.1) |
| Burkina Faso | 85.9 (75.3-92.8) | 98 (95.4-99.3) | 99.3 (97.4-99.9) |  | 35.5 (22.1-51.3) | 81.4 (63.6-92.6) | 93.3 (76.1-99.2) |
| Cameroon | 75.8 (58.4-87.6) | 85.9 (73.9-93.2) | 89.6 (69.8-97.7) |  | 40.8 (24.2-59.2) | 57.1 (39.4-72.5) | 67.2 (33.5-89.8) |
| Central African Republic | 64.3 (39.3-84.6) | 76.9 (51.9-92.6) | 80.5 (33.1-98.6) |  | 33.8 (14.7-58.3) | 49.2 (20.7-74.7) | 59.1 (11.5-94.3) |
| Chad | 32.1 (16.6-51.4) | 63.1 (46.6-77.8) | 79.1 (53.3-94.2) |  | 6.8 (3.2-12.7) | 21.2 (11.9-32.8) | 40.2 (14.3-70.9) |
| Congo | 93.2 (86.4-97.3) | 98 (96.1-99.1) | 99 (96.8-99.8) |  | 70.9 (52.4-84.6) | 89.6 (81.8-94.6) | 94.6 (84.1-98.9) |
| Cote d'Ivoire | 69.4 (47.3-86.3) | 90.8 (83-95.6) | 95.8 (87.6-99.2) |  | 33.1 (15.7-55.3) | 67.6 (52.1-80.3) | 83.4 (59.3-95.8) |
| Democratic Republic of the Congo | 83.4 (68.8-92.2) | 94.3 (90.8-96.8) | 97 (92.4-99.2) |  | 50.1 (31-69.4) | 76.1 (64.7-84.7) | 86.3 (69-95.8) |
| Ghana | 56 (40-70.5) | 90.8 (84-95.2) | 97.2 (91.9-99.3) |  | 26.2 (15.9-39.2) | 73.1 (59.1-84) | 90.6 (75.4-97.6) |
| Guinea | 54.1 (34.1-71.6) | 81.4 (73.1-88.6) | 90.9 (80.6-96.8) |  | 19.6 (9.7-33.5) | 47 (35.1-59.2) | 67.7 (45.4-85.6) |
| Liberia | 29.3 (12.6-52.2) | 86.1 (76.1-92.6) | 97.1 (91.5-99.4) |  | 15.9 (5.7-31.9) | 73.6 (59.6-84.4) | 93.8 (84-98.6) |
| Mali | 76.8 (65.5-85.8) | 93.3 (89.7-95.8) | 97.2 (93.7-98.9) |  | 32.6 (20.1-46.6) | 66.5 (55.1-75.8) | 83.5 (68.6-92.8) |
| Niger | 56.3 (40.3-70.7) | 89.3 (79.8-95.4) | 96.3 (89-99.3) |  | 9.4 (5.1-15.5) | 41.2 (22.5-61.5) | 69.6 (37-92.1) |
| Nigeria | 42 (29.4-55.7) | 51.6 (40.7-61.8) | 57.6 (38.5-75.5) |  | 16.7 (10.1-25.1) | 22.7 (15.8-31) | 28 (14.4-44.8) |
| Sao Tome and Principe | 76.7 (49.3-93.3) | 97.3 (94.5-99) | 99.2 (97-99.9) |  | 58.6 (27.6-83.8) | 93.5 (86.4-97.6) | 98.1 (92.4-99.9) |
| Senegal | 82.4 (70.2-91.1) | 94.5 (91.9-96.3) | 97.4 (94.3-99) |  | 46.6 (30.2-63.8) | 75.3 (66.8-82.8) | 86.9 (74-94.9) |
| Sierra Leone | 13.2 (6.6-22.9) | 84.2 (75-90.1) | 98.2 (95.3-99.5) |  | 8.3 (4.2-14.9) | 76.2 (64.3-84.7) | 97 (92.6-99.1) |
| The Gambia | 55.5 (35.8-73.4) | 91.3 (86-95.1) | 97.6 (93.9-99.3) |  | 26 (13.1-43.5) | 74.1 (62.3-84) | 91.7 (79.4-97.8) |
| Togo | 95.4 (89.4-98.3) | 94.4 (89.1-97.5) | 92.3 (74.1-98.6) |  | 63.8 (41.4-81.8) | 58.9 (39.3-77.2) | 55.1 (18.4-87.9) |
| **Latin America and Caribbean** |  |  |  |  |  |  |  |
| Dominican Republic | 98.4 (95.6-99.6) | 99.6 (99-99.9) | 99.8 (99.2-100) |  | 96.1 (89.7-98.9) | 98.9 (97.7-99.6) | 99.5 (98-100) |
| Haiti | 36.1 (17.7-57.9) | 60.4 (43.9-74.7) | 75.7 (45.9-92.3) |  | 16.5 (6.9-31.6) | 34.6 (21.2-49.5) | 53.6 (22.8-82) |
| Honduras | 83.2 (66.1-93.1) | 97.1 (93.5-99.1) | 98.9 (96-99.9) |  | 50.5 (29.1-72.3) | 87 (74.7-95.1) | 94.9 (81.4-99.5) |
| **Central and Eastern Europe** |  |  |  |  |  |  |  |
| Albania | 81.4 (60-93.9) | 99.4 (98.8-99.7) | 99.9 (99.8-100) |  | 84.7 (67.2-94.9) | 99.5 (99.2-99.8) | 100 (99.9-100) |
| Armenia | 99.8 (99.6-99.9) | 99.9 (99.7-99.9) | 99.9 (99.7-100) |  | 99.8 (99.6-99.9) | 99.9 (99.8-99.9) | 99.9 (99.7-100) |
| Kyrgyzstan | 99.5 (98.9-99.8) | 99.9 (99.9-100) | 100 (100-100) |  | 97.2 (94.2-98.9) | 99.7 (99.6-99.8) | 99.9 (99.9-100) |
| Tajikistan | 78.7 (64.6-89.2) | 96.1 (93.3-98) | 98.8 (96.7-99.7) |  | 60.7 (41.2-77.9) | 90.9 (85-95.1) | 97.1 (92.6-99.2) |

INSD, institutional delivery; SBA, skilled birth attendants; CrI, credible intervals

Table 6: Coverage of SBA among adolescents according to area of residence in 54 low- and middle-income countries, 2000-2030

| **Country** | Urban (predicted coverage (95% CrI) | | |  | Rural (predicted coverage (95% CrI) | | |
| --- | --- | --- | --- | --- | --- | --- | --- |
|  | **2000** | **2018** | **2030** |  | **2000** | **2018** | **2030** |
| **South Asia** |  |  |  |  |  |  |  |
| Afghanistan | 26.7 (13.5-43.8) | 88.5 (79.6-94.3) | 98.3 (94.8-99.6) |  | 6.4 (2.5-12.7) | 59.1 (44.3-72.5) | 91.9 (78.5-97.9) |
| Bangladesh | 19.3 (11.2-30.1) | 74.2 (62.3-84.8) | 93.6 (84.5-98.4) |  | 8.6 (4.7-14.1) | 53.2 (39.6-66.4) | 85.6 (67.6-95.6) |
| India | 63 (47.4-76.7) | 93.6 (87.9-97) | 98.2 (94.9-99.6) |  | 33.9 (19.8-48.6) | 81.3 (68.8-90.7) | 94.3 (84.8-98.6) |
| Maldives | 97.5 (92.7-99.4) | 100 (99.9-100) | 100 (100-100) |  | 86.8 (68.9-96.3) | 99.8 (99.5-99.9) | 100 (99.9-100) |
| Nepal | 40.9 (27.5-55.2) | 88.5 (81.5-93.2) | 97.4 (93.3-99.1) |  | 14.3 (8.4-22.5) | 64.9 (50.7-76.3) | 89.9 (77-96.5) |
| Pakistan | 46 (23.2-70.3) | 80.5 (69.9-89.2) | 91.5 (76.8-98) |  | 26.9 (10.6-49.8) | 63.3 (48.2-75.9) | 82.2 (58.9-94.8) |
| **East Asia and the Pacific** |  |  |  |  |  |  |  |
| Cambodia | 65.3 (51.8-77.1) | 98.6 (97.4-99.3) | 99.9 (99.6-100) |  | 30.6 (20.2-41.7) | 94.1 (89.6-96.8) | 99.4 (98.4-99.8) |
| Indonesia | 69.5 (55.3-81.6) | 93.4 (88.8-96.5) | 97.8 (94.7-99.4) |  | 45.5 (30.6-60.6) | 83.6 (74.1-90.6) | 94.2 (85.6-98.3) |
| Laos | 39.3 (26.9-53.6) | 83.2 (74.1-89.9) | 95 (88.8-98.2) |  | 10.5 (6.1-16.3) | 47.6 (33.7-61.3) | 77.8 (57.1-90.6) |
| Myanmar | 79 (49.1-94.5) | 87.8 (77.1-94.1) | 89.2 (63.4-98.5) |  | 48.1 (16.3-79.6) | 61.4 (43.3-76.8) | 67.8 (29.9-93.2) |
| Papua New Guinea | 67.8 (23-95.7) | 89 (78.7-95.3) | 94.2 (81.1-99) |  | 32.3 (5.3-77.5) | 58.2 (38.9-76) | 74.9 (43.4-93.9) |
| Philippines | 68.1 (50.4-80.5) | 93.1 (88.3-96.1) | 97.7 (94.4-99.3) |  | 38.3 (23.9-55) | 79.2 (68.1-87.9) | 92.3 (81.8-97.8) |
| Timor-Leste | 34.3 (10.4-66) | 85.7 (73.3-93.4) | 96.3 (85.8-99.6) |  | 12.1 (2.4-32.2) | 59.4 (40.6-77.2) | 87 (59.5-98.3) |
| Vietnam | 98.9 (98.2-99.4) | 99.7 (99.4-99.9) | 99.9 (99.6-100) |  | 66.8 (55-77.8) | 88.1 (78.6-93.5) | 94.2 (84.8-98.3) |
| **Eastern and Southern Africa** |  |  |  |  |  |  |  |
| Angola | 39.5 (5.9-86.6) | 73.2 (54.1-87.7) | 84.9 (44.9-98.6) |  | 17.7 (1.6-60.6) | 42.4 (25.6-59.6) | 64.8 (18.6-94.5) |
| Burundi | 53.3 (40-67.4) | 97.6 (95.8-98.8) | 99.8 (99.5-99.9) |  | 22.4 (14.4-32.4) | 91.2 (86.2-94.9) | 99.1 (97.9-99.7) |
| Comoros | 80.2 (68.6-89) | 95.7 (90.7-98.4) | 98.5 (94.9-99.8) |  | 58 (42.9-72.2) | 88.4 (77.7-95) | 95.6 (86.9-99.2) |
| Ethiopia | 37.8 (25.4-51.8) | 83.5 (73.6-90.6) | 95.2 (88.8-98.5) |  | 4.4 (2.5-7.1) | 28.4 (17.4-41.5) | 61.9 (36.1-82.3) |
| Kenya | 56.6 (42.9-70.1) | 87.1 (75.2-94.1) | 94.9 (81.4-99.2) |  | 31.1 (20.6-43.3) | 70.3 (52.3-83.9) | 87.1 (61.3-97.4) |
| Lesotho | 78.1 (63.5-88.7) | 94.4 (88.5-97.5) | 97.7 (92-99.6) |  | 49.9 (31.6-69.2) | 82.1 (68.8-91.5) | 92.1 (78-98.5) |
| Madagascar | 66.3 (53-78.5) | 83.8 (66.9-94.2) | 90.8 (69.6-98.6) |  | 33.2 (21.2-47.8) | 57.6 (33.7-79.5) | 73.4 (37.4-94.6) |
| Malawi | 71.7 (60.2-80.1) | 96.7 (94.5-98.1) | 99.3 (98.4-99.7) |  | 47 (35.6-58.6) | 91 (85.7-94.7) | 98 (95.4-99.3) |
| Mozambique | 76.2 (62.2-87.3) | 89.4 (81.4-94.9) | 93.5 (81.5-98.5) |  | 42.5 (26.4-60.8) | 66 (48.2-79.2) | 77.6 (50.3-93.4) |
| Rwanda | 62.4 (49.4-74.8) | 97.6 (95.7-98.7) | 99.7 (99.1-99.9) |  | 41 (28.8-54.2) | 94.3 (90.1-97) | 99.3 (97.9-99.8) |
| Tanzania | 73 (54.5-86.8) | 90.2 (83.2-95) | 95 (85.9-98.7) |  | 37.1 (21.7-56.5) | 66 (49.3-80.4) | 80.3 (54.8-94.9) |
| Uganda | 72.5 (59.6-83) | 92 (86.2-95.7) | 96.7 (91.9-99) |  | 41.4 (27.2-55.9) | 75.4 (63.1-85.4) | 88.8 (75.4-96.2) |
| Zambia | 67.9 (53.8-80.1) | 94 (90-96.8) | 98.3 (96.2-99.4) |  | 29.6 (18.1-43.3) | 75.3 (64.2-84.8) | 91.9 (83.6-97) |
| Zimbabwe | 74.8 (57.5-87.3) | 90.7 (84.4-94.8) | 95.4 (86.7-98.8) |  | 50 (31.1-69.5) | 76.2 (63.8-85.7) | 87.3 (67.8-96.5) |
| **West and Central Africa** |  |  |  |  |  |  |  |
| Benin | 83.4 (70.2-92.8) | 89.3 (83.4-93.7) | 92.2 (79.3-98) |  | 68.9 (51.1-84.1) | 78.4 (69.8-85.6) | 84.2 (63.7-95.1) |
| Burkina Faso | 82.6 (70.9-91) | 95.6 (90.3-98.5) | 98 (92.6-99.8) |  | 47.3 (31.6-63.6) | 80.5 (64-92) | 90.5 (70-98.8) |
| Cameroon | 78.6 (68-86.7) | 89.7 (82.5-95) | 93.9 (85.5-98.4) |  | 41.5 (29.8-54.2) | 62.8 (47.2-76) | 75.5 (52-90.9) |
| Central African Republic | 72.3 (58.9-83.2) | 83.8 (69.2-93.3) | 88.5 (66.6-97.9) |  | 33.9 (22.4-46.3) | 51.3 (30.3-71.2) | 63.2 (28.3-89.2) |
| Chad | 44.4 (30.4-58.2) | 71.2 (55.4-82.6) | 83.4 (63.9-94.2) |  | 9.7 (5.8-14.8) | 25.4 (15.2-38.2) | 42.8 (20.3-68.5) |
| Congo | 95.5 (91-98.2) | 98 (96.3-99) | 98.6 (95.5-99.7) |  | 81 (65.8-91.4) | 90.4 (83.3-94.9) | 93.4 (80.9-98.6) |
| Cote d'Ivoire | 73.6 (52.9-88) | 93.5 (87.9-97.1) | 97.5 (92.3-99.5) |  | 35.3 (18.2-55.1) | 72.9 (56.7-85.2) | 88 (68.7-97.3) |
| Democratic Republic of the Congo | 88.8 (82.6-93.5) | 95.8 (93.2-97.6) | 97.8 (94.9-99.3) |  | 57.2 (42.9-70.4) | 79.3 (71.2-86.9) | 88.5 (76-95.7) |
| Ghana | 55.2 (39.4-70.5) | 91.4 (85-95.6) | 97.6 (93.2-99.5) |  | 25.1 (15-36.8) | 74.2 (60-85.5) | 91.7 (76.9-98.2) |
| Guinea | 71.4 (53.4-84.5) | 89.8 (84-93.9) | 95 (88.5-98.3) |  | 24.6 (12-40.7) | 52.5 (40.5-64.1) | 71.4 (48.9-86.9) |
| Liberia | 66.9 (40-87.5) | 85.8 (70.4-94.5) | 91.4 (69.4-99) |  | 36 (14-63.2) | 61.5 (39.9-80.8) | 75.5 (38.2-96.2) |
| Mali | 80.1 (68.9-88.4) | 93.3 (89.9-96) | 97 (93.8-98.8) |  | 35.9 (23.6-50.4) | 65.5 (54.2-76.1) | 81.6 (66.5-91.9) |
| Niger | 63.7 (47.5-77.4) | 86.2 (74-93.9) | 92.9 (78.4-98.6) |  | 19.3 (11-29.5) | 46.8 (26.8-67.8) | 66.8 (31.4-90.1) |
| Nigeria | 46.6 (33.9-60.7) | 56.8 (44.9-67.9) | 63.2 (43.6-80.7) |  | 17.9 (10.5-27.1) | 24.6 (17-33.5) | 30.7 (15.3-51) |
| Sao Tome and Principe | 86.4 (77-92.7) | 97 (94.4-98.6) | 98.9 (97-99.7) |  | 73.7 (59.1-84.4) | 93.4 (87.5-96.9) | 97.6 (93.3-99.4) |
| Senegal | 82.4 (74.2-89) | 84.7 (79-89.3) | 85.5 (74.8-93.2) |  | 46.9 (34.4-59.3) | 50.9 (40.6-61) | 53.3 (34.7-71.5) |
| Sierra Leone | 51.9 (39.2-63.9) | 86.7 (80.3-92) | 95.5 (90.8-98.3) |  | 31.4 (21-42.7) | 73.3 (61.5-82.5) | 89.8 (79-96.2) |
| The Gambia | 61.5 (42.5-78.5) | 91.8 (86.5-95.3) | 97.5 (93.5-99.3) |  | 29.9 (15.5-47.9) | 74.3 (62.3-84.4) | 90.9 (78.5-97.3) |
| Togo | 86.3 (73.9-94.8) | 90.4 (82.5-95.4) | 91.2 (74.3-98.5) |  | 40.5 (20.8-63.1) | 49.5 (30-69.1) | 55.8 (20.5-86.7) |
| **Latin America and Caribbean** |  |  |  |  |  |  |  |
| Dominican Republic | 98.7 (96.7-99.6) | 99.6 (99.1-99.9) | 99.8 (99.3-100) |  | 97.4 (93.6-99.2) | 99.2 (98.4-99.7) | 99.6 (98.7-100) |
| Haiti | 39.5 (19.7-63.4) | 62.8 (47.9-76.3) | 77.5 (48.6-92.9) |  | 18.7 (7.8-34.9) | 36.9 (22.9-52.8) | 55.9 (25.5-82.6) |
| Honduras | 87.1 (73.4-95) | 97.1 (93.5-99) | 98.7 (94.9-99.9) |  | 56.6 (35.1-76.6) | 86 (71.4-94.1) | 93.5 (76.5-99.2) |
| **Central and Eastern Europe** |  |  |  |  |  |  |  |
| Albania | 96.6 (91.4-99) | 99.8 (99.6-99.9) | 100 (99.9-100) |  | 97.8 (94.3-99.3) | 99.9 (99.8-99.9) | 100 (100-100) |
| Armenia | 99.8 (99.6-99.9) | 99.9 (99.8-99.9) | 99.9 (99.8-100) |  | 99.8 (99.6-99.9) | 99.9 (99.8-99.9) | 99.9 (99.8-100) |
| Kyrgyzstan | 99.8 (99.6-99.9) | 99.9 (99.9-100) | 100 (99.9-100) |  | 99.3 (98.6-99.7) | 99.7 (99.5-99.8) | 99.9 (99.7-100) |
| Tajikistan | 87.2 (80-92.6) | 95.4 (92.5-97.5) | 97.8 (94.8-99.3) |  | 79.3 (68-87.6) | 92.2 (86.9-95.5) | 96.2 (91.3-98.7) |

Table 7: Coverage of institutional delivery among adolescent according to wealth quintile in LMICs, 2000-2030

| Country | **Predicted coverage (95% credible intervals)** | | | | | | | |
| --- | --- | --- | --- | --- | --- | --- | --- | --- |
|  | 2000 | |  | 2018 | |  | 2030 | |
|  | Q1 | Q5 |  | Q1 | Q5 |  | Q1 | Q5 |
| **South Asia** |  |  |  |  |  |  |  |  |
| Afghanistan | 2.1 (0.2-9.3) | 25.9 (3.6-68.5) |  | 39.5 (19.4-63.1) | 92.1 (80.2-97.8) |  | 85.5 (43.9-99.1) | 98.9 (93.7-100) |
| Bangladesh | 2.5 (1.2-4.9) | 19.7 (9.7-35.8) |  | 29.4 (16.6-44.6) | 78.9 (63.6-89.8) |  | 71.6 (38.2-92.4) | 95.4 (85.1-99.2) |
| India | 9.5 (3-22.7) | 53 (23.5-80.8) |  | 78.6 (63.2-90.6) | 97.6 (93.8-99.3) |  | 97.1 (89.7-99.6) | 99.7 (98.9-100) |
| Maldives | 98.8 (95.8-99.8) | 94.6 (81.2-99.3) |  | 98.9 (97.7-99.6) | 94.4 (81.9-99.1) |  | 98.4 (92.9-99.9) | 92.1 (65.4-99.6) |
| Nepal | 4 (1.9-7.3) | 30.2 (16.8-47.7) |  | 59 (41.8-73.1) | 93.5 (88-97.1) |  | 93.7 (82.6-98.3) | 99.3 (98.2-99.8) |
| Pakistan | 11.3 (3.5-26.7) | 49.4 (22.1-77.6) |  | 53.5 (35.8-70) | 90.3 (81.3-95.9) |  | 82.7 (56.8-95.7) | 97.4 (91.7-99.6) |
| **East Asia and the Pacific** |  |  |  |  |  |  |  |  |
| Cambodia | 1.5 (0.8-2.8) | 29.5 (15.6-47.3) |  | 89.6 (80.5-95.3) | 99.6 (99-99.8) |  | 99.8 (99.4-100) | 100 (100-100) |
| Indonesia | 13.1 (6.1-23.4) | 50.7 (30.6-71.4) |  | 55.1 (37-72.4) | 89.3 (79.9-95.5) |  | 82.4 (57.2-95.3) | 96.9 (90.8-99.4) |
| Laos | 2.3 (0.7-5.7) | 30.8 (12.5-59.5) |  | 43 (26.1-60.7) | 93.3 (85.6-97.5) |  | 87.3 (64.6-97.3) | 99.2 (97.1-99.9) |
| Myanmar | 8 (0.2-39.8) | 70.3 (10.1-98.6) |  | 27.3 (11.3-48.3) | 96.3 (89.5-99.2) |  | 58.6 (9.4-96.3) | 98.3 (90.2-100) |
| Papua New Guinea | 17.4 (0.4-74) | 58.9 (5.8-97.9) |  | 43 (22.7-65.3) | 91.2 (77.1-97.6) |  | 69.5 (20.6-96.5) | 96 (79.8-99.8) |
| Philippines | 10.1 (4.1-19.9) | 57.4 (37.7-75.7) |  | 65.3 (48-79.5) | 95.9 (91.6-98.3) |  | 92 (78.3-98.1) | 99.3 (97.7-99.9) |
| Timor-Leste | 2.1 (0.3-7.4) | 19.8 (3.1-53.1) |  | 36.2 (17.9-59.1) | 86.6 (70.9-95.4) |  | 82.6 (43.3-98.3) | 98 (90.3-99.9) |
| Vietnam | 26.8 (13.7-42.7) | 99.4 (98.7-99.8) |  | 95.4 (90.6-98.2) | 100 (100-100) |  | 99.6 (98.8-99.9) | 100 (100-100) |
| **Eastern and Southern Africa** |  |  |  |  |  |  |  |  |
| Angola | 33.1 (10-63) | 79.9 (40.4-97.5) |  | 33.2 (16.8-53.4) | 82.9 (62.6-94.6) |  | 36.6 (7.6-78.6) | 82 (46.4-97.3) |
| Burundi | 9 (3.9-18.7) | 34.1 (14.3-59.6) |  | 86.4 (76.1-93) | 97 (93.5-98.9) |  | 99 (96.6-99.8) | 99.8 (99.3-100) |
| Comoros | 32.6 (3.9-78.2) | 74.5 (30.3-97.9) |  | 77.4 (49.8-92.9) | 96.5 (88.6-99.5) |  | 88.6 (37.2-99.7) | 97.8 (85.5-100) |
| Ethiopia | 0.8 (0.4-1.4) | 15.8 (7.6-28.3) |  | 18 (9.2-29.2) | 82.4 (68.3-92) |  | 65.3 (37.1-86.7) | 97.5 (92.8-99.4) |
| Kenya | 34.3 (20.5-49.3) | 63.7 (44.1-79.9) |  | 58.8 (38.2-76) | 82.5 (65-93.2) |  | 74.5 (39.4-93.7) | 90 (66.7-98.4) |
| Lesotho | 28.4 (12.3-49.1) | 65.3 (41.1-84) |  | 75.4 (56.9-88.7) | 93.7 (85.8-98.1) |  | 91.4 (71.4-98.9) | 98 (91.9-99.8) |
| Madagascar | 10.5 (3.4-23.1) | 43.4 (17.9-70.4) |  | 41.5 (13.3-77.1) | 80 (49-96.4) |  | 67.2 (14.3-98.4) | 90.2 (52.9-99.8) |
| Malawi | 30.2 (18.9-43.7) | 72.2 (57.2-83.8) |  | 92 (86.6-95.8) | 98.6 (97.4-99.3) |  | 99 (97.7-99.7) | 99.8 (99.6-100) |
| Mozambique | 24.6 (11.6-43.7) | 86.4 (73.2-94.6) |  | 58.3 (38.7-75.4) | 96.6 (92-98.7) |  | 77.6 (46.9-94.9) | 98.4 (94-99.8) |
| Rwanda | 14.5 (8-24) | 44.9 (28-63.5) |  | 96.3 (92.9-98.4) | 99.2 (98.4-99.7) |  | 99.9 (99.6-100) | 100 (99.9-100) |
| Tanzania | 24.5 (10.2-43) | 78.3 (58.3-91.9) |  | 53.9 (32.9-73.7) | 93.1 (85.1-97.4) |  | 72.4 (37.4-93.5) | 96.5 (87.5-99.5) |
| Uganda | 29.9 (16.8-45.4) | 59.4 (38.7-77.7) |  | 73.7 (58.3-86.2) | 90.6 (81.8-95.9) |  | 90.2 (74.2-97.7) | 96.9 (90.9-99.4) |
| Zambia | 20.5 (9.9-35) | 70.8 (52.6-86.4) |  | 73.7 (59.8-85.4) | 96.5 (93.3-98.5) |  | 93.1 (83.2-97.9) | 99.3 (98-99.8) |
| Zimbabwe | 29.5 (13-51.8) | 74.5 (51.3-89.5) |  | 73.3 (58-85.9) | 95.3 (90.1-98.1) |  | 90.1 (71.8-98.3) | 98.5 (94.3-99.8) |
| **West and Central Africa** |  |  |  |  |  |  |  |  |
| Benin | 58.2 (36.3-78.8) | 97.8 (94.5-99.3) |  | 79.5 (69.3-87.8) | 99.2 (98.5-99.7) |  | 88.9 (71.1-97.3) | 99.6 (98.7-99.9) |
| Burkina Faso | 16.1 (7.2-29.6) | 79.8 (60.2-92.3) |  | 80 (56.5-94.2) | 98.8 (95.8-99.8) |  | 95.4 (77.9-99.8) | 99.8 (98.6-100) |
| Cameroon | 29.8 (13.1-52.4) | 89 (74.8-96.4) |  | 44.2 (25.5-65.2) | 94 (85.5-98) |  | 55.6 (18.9-88.3) | 95.3 (81.8-99.5) |
| Central African Republic | 24.1 (7-50.7) | 76.9 (49.8-94) |  | 37.6 (11.5-70.5) | 85.6 (57.2-97.4) |  | 49.3 (4.1-94.3) | 85.8 (36.8-99.6) |
| Chad | 2.2 (0.8-4.9) | 28.3 (11.3-52.4) |  | 12.3 (5.3-23.4) | 69.8 (47.6-86.3) |  | 34.1 (8.1-68.5) | 87.1 (61.8-98) |
| Congo | 54.7 (32.1-75.8) | 96.6 (91.9-99) |  | 92 (84.4-96.6) | 99.7 (99.2-99.9) |  | 97.9 (92.7-99.7) | 99.9 (99.6-100) |
| Cote d’Ivoire | 18.7 (6.2-40) | 80.2 (55.4-94.2) |  | 62.6 (44-78.8) | 97.1 (93.2-99.1) |  | 85.5 (56.7-97.4) | 99.1 (96.7-99.9) |
| Democratic Republic of the Congo | 51.5 (27.9-74.7) | 99.4 (98.5-99.8) |  | 62.3 (45.4-76.2) | 99.6 (99.2-99.8) |  | 69.3 (36.5-90.6) | 99.7 (98.9-99.9) |
| Ghana | 15.3 (7.1-26.6) | 98.6 (97.1-99.4) |  | 65.1 (46.1-80.6) | 99.9 (99.7-100) |  | 88.7 (67.7-97.7) | 100 (99.9-100) |
| Guinea | 10.1 (4-20.5) | 56.6 (32.9-80.4) |  | 34.2 (21.4-49.3) | 86.3 (75.3-93.5) |  | 59.8 (32.3-83.2) | 94.3 (84.2-98.5) |
| Liberia | 12.6 (3.5-29.4) | 42.4 (15.4-72.9) |  | 61 (41-79.3) | 89.2 (77.8-95.9) |  | 88.2 (65.2-98.3) | 97.4 (90-99.7) |
| Mali | 18.6 (9.2-31.9) | 78.6 (63.8-89.5) |  | 50.7 (36.1-64.9) | 94.5 (90.3-97.2) |  | 74.7 (52.8-89.5) | 98 (94.9-99.4) |
| Niger | 4.4 (1.9-8.8) | 37.6 (19.8-59.4) |  | 32.3 (14.3-53.7) | 84.9 (66.7-95.1) |  | 67.4 (28-92.8) | 95.6 (83.6-99.5) |
| Nigeria | 8 (4.1-13.8) | 58.4 (40.5-74.8) |  | 14.2 (8.4-21.3) | 73 (60.2-83.5) |  | 21.9 (8.4-40.6) | 80.5 (62-92.6) |
| Sao Tome and Principe | 49.2 (13.4-83.5) | 98.9 (95.2-99.9) |  | 90.7 (78.6-97) | 99.9 (99.8-100) |  | 96.6 (83-99.9) | 100 (99.8-100) |
| Senegal | 23.3 (10.6-42.1) | 96.8 (93-98.9) |  | 68.7 (56.3-78.9) | 99.6 (99.3-99.8) |  | 88.9 (74.2-96.5) | 99.9 (99.7-100) |
| Sierra Leone | 6.1 (2.6-12.7) | 13.9 (5.4-28.9) |  | 71.5 (54.5-84) | 85.9 (74-93.7) |  | 96.3 (88.7-99.2) | 98.4 (94.9-99.7) |
| The Gambia | 9 (3.3-19.1) | 62.1 (37-82.7) |  | 79.2 (66.3-88.7) | 98.6 (97-99.4) |  | 97.7 (93-99.6) | 99.9 (99.5-100) |
| Togo | 46.5 (20.8-72.8) | 99.6 (98.9-99.9) |  | 41.1 (19.3-65.1) | 99.5 (98.8-99.9) |  | 40.1 (6.8-81.3) | 99.2 (96.4-99.9) |
| **Latin America and Caribbean** |  |  |  |  |  |  |  |  |
| Dominican Republic | 91.2 (75.9-98) | 98.8 (96-99.8) |  | 99.5 (98.7-99.9) | 99.9 (99.8-100) |  | 99.9 (99.5-100) | 100 (99.9-100) |
| Haiti | 9.6 (3-20.6) | 43.4 (18-71.2) |  | 26.9 (13.9-43.6) | 73 (53.7-87.4) |  | 50.1 (16.5-84) | 86.3 (61.3-97.5) |
| Honduras | 33.3 (12-62.1) | 90.1 (74.1-97.7) |  | 87.7 (71-96.3) | 99.3 (97.9-99.9) |  | 96.6 (82.7-99.8) | 99.8 (99.1-100) |
| **Central and Eastern Europe** |  |  |  |  |  |  |  |  |
| Albania | 98 (93.8-99.6) | 99.8 (99.2-100) |  | 99.4 (98.9-99.7) | 99.9 (99.8-100) |  | 99.7 (98.8-100) | 100 (99.9-100) |
| Armenia | 99.6 (99.2-99.9) | 99.9 (99.7-100) |  | 99.8 (99.5-99.9) | 99.9 (99.8-100) |  | 99.8 (99.2-100) | 99.9 (99.8-100) |
| Kyrgyzstan | 98.1 (95.8-99.4) | 99.7 (99.2-99.9) |  | 99.6 (99.4-99.8) | 99.9 (99.9-100) |  | 99.9 (99.6-100) | 100 (99.9-100) |
| Tajikistan | 47.5 (24.8-70.8) | 88.7 (73.9-96.3) |  | 88.8 (79.3-95.1) | 98.7 (97.2-99.5) |  | 97 (91-99.4) | 99.7 (98.9-99.9) |

CrI, credible intervals

Table 8: Coverage of skilled birth attendants among adolescent according to wealth quintile in LMICs, 2000-2030

| Country | **Predicted coverage (95% credible intervals)** | | | | | | | |
| --- | --- | --- | --- | --- | --- | --- | --- | --- |
|  | 2000 | |  | 2018 | |  | 2030 | |
|  | Q1 | Q5 |  | Q1 | Q5 |  | Q1 | Q5 |
| **South Asia** |  |  |  |  |  |  |  |  |
| Afghanistan | 7 (0.8-22) | 48.7 (11.8-86.1) |  | 38.5 (21.1-59.1) | 90.3 (78.5-97) |  | 75.4 (28.4-97.4) | 97.4 (86.7-99.9) |
| Bangladesh | 3.2 (1.4-6.2) | 24.1 (11.7-41.5) |  | 38.3 (23.7-54.6) | 84.8 (72.1-93.3) |  | 80.8 (55-95.2) | 97.3 (91.7-99.6) |
| India | 17.7 (5.7-38.1) | 66 (34.9-89.2) |  | 77.6 (60.7-89.7) | 97.1 (92.6-99.1) |  | 95 (83-99.3) | 99.5 (97.9-99.9) |
| Maldives | 92.3 (76.9-98.4) | 98.4 (94.4-99.8) |  | 99.9 (99.7-100) | 100 (99.9-100) |  | 100 (100-100) | 100 (100-100) |
| Nepal | 4.8 (2.3-8.6) | 38.6 (22-56.8) |  | 57.7 (42.6-72.5) | 94.4 (89.3-97.4) |  | 92.6 (82.1-97.9) | 99.4 (98.3-99.9) |
| Pakistan | 16.6 (6.3-35.8) | 60.9 (34.3-84.6) |  | 57.2 (39.9-73.2) | 91.8 (83.5-96.7) |  | 82.3 (56.8-95.6) | 97.4 (91.7-99.5) |
| **East Asia and the Pacific** |  |  |  |  |  |  |  |  |
| Cambodia | 13.1 (6.9-21.1) | 83.8 (71.4-92.5) |  | 91.8 (85.3-96.2) | 99.8 (99.5-99.9) |  | 99.5 (98.4-99.9) | 100 (100-100) |
| Indonesia | 29.3 (15.7-45.6) | 77.2 (59-89.8) |  | 82.7 (71.9-91.2) | 97.6 (95.1-99) |  | 95.9 (89.3-99.1) | 99.5 (98.6-99.9) |
| Laos | 3.5 (1.7-6.3) | 44.3 (26-63.2) |  | 40 (24.9-57.3) | 93.4 (87.4-97.1) |  | 81.9 (60.9-94.4) | 99 (97.2-99.8) |
| Myanmar | 18.1 (0.9-63.1) | 95 (72.4-99.9) |  | 50.2 (27.3-74.2) | 99.6 (98.9-99.9) |  | 74 (22.8-98.1) | 99.8 (98.9-100) |
| Papua New Guinea | 14.9 (0.9-57.7) | 64.6 (14.4-97.1) |  | 39.2 (20.6-59.7) | 92.1 (81.6-97.8) |  | 66.3 (23.1-94.3) | 96.7 (86-99.7) |
| Philippines | 14.6 (6.5-26.7) | 87.3 (75.7-94.8) |  | 80 (66.8-89.4) | 99.4 (98.8-99.8) |  | 96.8 (90.8-99.3) | 99.9 (99.8-100) |
| Timor-Leste | 5.3 (0.8-17.1) | 30.8 (6.7-68.2) |  | 47 (24.2-69.3) | 87.8 (71.9-96) |  | 84.5 (45.2-98.7) | 97.5 (88.1-99.9) |
| Vietnam | 29.6 (18.2-42.4) | 99.7 (99.3-99.8) |  | 94.9 (89.6-97.8) | 100 (100-100) |  | 99.5 (98.5-99.9) | 100 (100-100) |
| **Eastern and Southern Africa** |  |  |  |  |  |  |  |  |
| Angola | 10.9 (0.5-48.8) | 43 (3.6-94.3) |  | 36.7 (20.3-56.4) | 84.1 (64.6-95) |  | 68.4 (17.4-97.2) | 93.9 (70.9-99.7) |
| Burundi | 12.2 (6.7-20.5) | 41.6 (24.3-59.5) |  | 88.4 (80.2-94.1) | 97.5 (94.9-98.9) |  | 99.1 (97.7-99.8) | 99.8 (99.5-100) |
| Comoros | 38.9 (22-57.8) | 81 (61.2-93) |  | 93.3 (85.3-97.8) | 99 (97.1-99.7) |  | 99 (96.2-99.9) | 99.9 (99.4-100) |
| Ethiopia | 1.6 (0.8-2.8) | 26.5 (13.5-42.1) |  | 13.4 (6.7-23.3) | 76.2 (58.2-88.7) |  | 41.9 (17.1-70.8) | 92.9 (81.5-98.4) |
| Kenya | 17.7 (9.6-29.2) | 56.2 (37.4-72.9) |  | 62.9 (40-80.9) | 90.7 (79.3-97) |  | 86.4 (56.6-98) | 97.2 (88.8-99.7) |
| Lesotho | 29.7 (13.8-48.6) | 67.6 (43.9-87) |  | 77.5 (60.9-90) | 94.6 (88-98.2) |  | 92.9 (77.3-99.1) | 98.5 (94.1-99.9) |
| Madagascar | 24.8 (13-39.5) | 91.1 (82.9-96.2) |  | 29.1 (10.7-54.7) | 91.7 (79-97.9) |  | 40.1 (7.9-82) | 93 (71.1-99.5) |
| Malawi | 35.6 (23.9-49.5) | 70.9 (56.6-82.5) |  | 88.5 (82.1-93.9) | 97.2 (94.6-98.7) |  | 97.6 (94.5-99.3) | 99.4 (98.5-99.8) |
| Mozambique | 29.4 (14.2-48.4) | 84.1 (69-93.4) |  | 55.5 (37.2-72.6) | 94.2 (87.6-97.9) |  | 71.9 (40.7-92.3) | 96.8 (89-99.5) |
| Rwanda | 23.4 (13.3-37) | 56.8 (38.7-74.3) |  | 95.9 (92.3-98.1) | 99 (97.9-99.6) |  | 99.8 (99.3-100) | 100 (99.8-100) |
| Tanzania | 23.3 (9.1-42) | 76.2 (53.9-91.3) |  | 55.9 (36.1-73.4) | 93.3 (85.3-97.6) |  | 76.1 (44.2-94.5) | 97 (89.2-99.6) |
| Uganda | 31.8 (17.5-49) | 62.4 (41.9-79.8) |  | 71.9 (55.9-84.6) | 90.2 (80.6-95.7) |  | 88.5 (71.1-97) | 96.4 (89.3-99.2) |
| Zambia | 18.8 (9.3-31.5) | 71.6 (52.9-86.5) |  | 67.4 (53-80) | 95.9 (92.4-98.3) |  | 90 (77.9-96.8) | 99 (97.5-99.7) |
| Zimbabwe | 32.2 (14.9-54.8) | 79.3 (58.8-91.5) |  | 72.2 (57.2-83.8) | 95.7 (91.4-98.2) |  | 89.2 (69.5-97.8) | 98.6 (94.8-99.8) |
| **West and Central Africa** |  |  |  |  |  |  |  |  |
| Benin | 61.7 (38.4-80.1) | 96 (90.4-98.8) |  | 68 (54.6-78.7) | 97.1 (94.4-98.7) |  | 76 (44.5-93.8) | 97.8 (92-99.6) |
| Burkina Faso | 27.4 (14.3-46) | 79.5 (62.3-91.4) |  | 72.7 (48.4-91.1) | 96.4 (89.2-99.2) |  | 88.5 (58.9-99.1) | 98.6 (92.8-99.9) |
| Cameroon | 26.4 (15.9-39.1) | 92.8 (86.6-96.8) |  | 54.7 (36.9-72.1) | 97.8 (95.1-99.1) |  | 73.6 (44.7-91.8) | 98.9 (96.8-99.8) |
| Central African Republic | 22.8 (12.6-36.2) | 83.5 (69.1-92.9) |  | 43.3 (19.8-67.8) | 92.4 (80.6-97.9) |  | 59.5 (19.2-90.5) | 95.1 (81.3-99.5) |
| Chad | 4.3 (2.2-7.6) | 43.8 (25.8-62.2) |  | 13.6 (6.9-24.2) | 72.1 (54.2-87.6) |  | 28.1 (9.6-55.9) | 84.7 (63.4-96.5) |
| Congo | 84.1 (67.2-93.6) | 96.9 (92-99.1) |  | 87.7 (75.7-94.4) | 97.7 (95-99.2) |  | 87.8 (58.7-98.1) | 97.6 (90-99.7) |
| Cote d'Ivoire | 22.1 (8-42.4) | 84 (62.6-95.4) |  | 66.9 (46.9-82.1) | 97.7 (94.4-99.3) |  | 87.6 (63-98.1) | 99.3 (97.1-99.9) |
| Democratic Republic of the Congo | 36.6 (21.8-53.3) | 98.8 (97.7-99.5) |  | 74.4 (61.6-84.6) | 99.8 (99.6-99.9) |  | 90.3 (77.1-97.2) | 99.9 (99.8-100) |
| Ghana | 14.9 (6.7-25.5) | 98.5 (97-99.4) |  | 65.3 (46.5-80.8) | 99.9 (99.7-100) |  | 89.2 (68.5-98) | 100 (99.9-100) |
| Guinea | 11.3 (4.4-22.4) | 80.4 (62.3-92.7) |  | 34.6 (22.3-48.4) | 94.8 (90.3-97.6) |  | 59.2 (33.4-81.4) | 97.9 (94.3-99.5) |
| Liberia | 22.7 (6.3-47.5) | 69.8 (35.7-91.9) |  | 54.7 (26.2-79.4) | 91 (75.8-97.8) |  | 75.3 (28.7-97.6) | 95.7 (78.6-99.7) |
| Mali | 22.7 (11.8-37.3) | 81.9 (68.2-91.4) |  | 50.9 (36.6-64.7) | 94.3 (89.5-97.3) |  | 73.3 (51.1-89) | 97.7 (94.3-99.4) |
| Niger | 12.5 (5.6-23.1) | 52.3 (30.5-71.3) |  | 33.7 (16.4-57.2) | 78.8 (57.7-91.8) |  | 54.7 (20.5-87.2) | 88.5 (63.3-98.1) |
| Nigeria | 8.4 (4.2-14.5) | 64 (46.2-78.4) |  | 16.2 (10.1-24.4) | 79 (67.4-87.7) |  | 26.2 (11.2-47.8) | 86.2 (69.3-94.9) |
| Sao Tome and Principe | 77.5 (61.7-88.8) | 99.9 (99.7-100) |  | 81.6 (67.7-91.5) | 99.9 (99.8-100) |  | 83.1 (57.6-96.2) | 99.9 (99.7-100) |
| Senegal | 32.8 (21-47) | 91.9 (86-95.8) |  | 43.3 (32-54.4) | 94.7 (91.5-97.1) |  | 51.5 (31.1-70.8) | 96 (91.4-98.4) |
| Sierra Leone | 24.3 (14.7-37.6) | 58.1 (41.2-72.6) |  | 65.2 (49.2-78.1) | 89 (80.5-94.6) |  | 85.8 (69.2-95.2) | 96.3 (90.1-99) |
| The Gambia | 11.2 (4-22.5) | 70.3 (46.3-87.4) |  | 78.9 (65.5-88.3) | 98.7 (97.3-99.5) |  | 97.3 (91.6-99.5) | 99.9 (99.5-100) |
| Togo | 20.2 (6.7-42.6) | 95.1 (87.5-98.7) |  | 36.7 (18.7-58.7) | 98 (95.2-99.4) |  | 52.6 (15-88.4) | 98.6 (94.5-99.9) |
| **Latin America and Caribbean** |  |  |  |  |  |  |  |  |
| Dominican Republic | 95.5 (86.9-99.1) | 99.4 (98-99.9) |  | 99.4 (98.4-99.8) | 99.9 (99.7-100) |  | 99.8 (99-100) | 100 (99.8-100) |
| Haiti | 10.6 (3.4-24.7) | 46.8 (20.8-74.9) |  | 28 (14.9-43.9) | 74.7 (55.5-88.4) |  | 53.6 (20.4-84.1) | 88.5 (68.2-97.9) |
| Honduras | 37.1 (14.5-63.2) | 92.2 (78.5-98) |  | 84.8 (66.1-95.1) | 99.2 (97.6-99.8) |  | 94.9 (76.1-99.7) | 99.7 (98.5-100) |
|  |  |  |  |  |  |  |  |  |
| **Central and Eastern Europe** |  |  |  |  |  |  |  |  |
| Albania | 98.7 (96.3-99.7) | 99.7 (99.1-100) |  | 99.7 (99.4-99.9) | 99.9 (99.9-100) |  | 99.9 (99.7-100) | 100 (99.9-100) |
| Armenia | 99.7 (99.3-99.9) | 99.9 (99.7-100) |  | 99.8 (99.6-99.9) | 99.9 (99.8-100) |  | 99.9 (99.5-100) | 100 (99.8-100) |
| Kyrgyzstan | 99.2 (98.2-99.7) | 99.9 (99.6-100) |  | 99.6 (99.3-99.8) | 99.9 (99.9-100) |  | 99.8 (99.4-100) | 100 (99.9-100) |
| Tajikistan | 73.5 (58.1-85.5) | 92.2 (85.3-96.3) |  | 88.1 (78.9-94) | 97 (93.7-98.7) |  | 93.9 (83.9-98.4) | 98.5 (95.9-99.6) |

Table 9: Changes in the magnitude of socio-economic inequality in access to delivery care, 2000-2030

| **Country** | INSD (SII) | | |  | SBA (SII) | | |
| --- | --- | --- | --- | --- | --- | --- | --- |
|  | **2000** | **2018** | **2030** |  | **2000** | **2018** | **2030** |
| **South Asia** |  |  |  |  |  |  |  |
| Afghanistan | 26.5(-4.7-57.6) | 63.6(55-72.3) | 16.1(11-21.1) |  | 48(3.5-92.5) | 63.4(49.9-76.8) | 27(24.3-29.6) |
| Bangladesh | 20.3(3.3-37.2) | 61.6(50.6-72.6) | 29.6(23.7-35.5) |  | 1.3(.3-2.2) | .30 (.01-.5) | .1(.04-.3) |
| India | 52.5(24.3-80.6) | 23.1(12.6-33.6) | 3.1(1.1-5.1) |  | 39(20.5-57.5) | 59.4(57.9-60.8) | 31.6(27.6-35.6) |
| Maldives | -4.1(-13.9-5.7) | -4.4(-14.8-6) | -6.2(-20.7-8.4) |  | .3(.01-.5) | .1(-.1-.3) | .1(.04-.3) |
| Nepal | 29.7(.5-58.9) | 41.6(35.6-47.6) | 6.7(4-9.4) |  | 24.8(4.9-44.6) | 58.4(53.4-63.3) | 20.6(15.3-25.8) |
| Pakistan | 44.2(9.4-79) | 44.4(37.1-51.7) | 17.7(14.8-20.6) |  | 42.3(36.6-47.9) | 35.9(28.1-43.6) | 26.8(20.8-32.8) |
| **East Asia and the Pacific** |  |  |  |  |  |  |  |
| Cambodia | 30.8(-9-70.5) | 11.9(5.5-18.3) | .2(.1-.4) |  | 63.7(42.4-85) | 29.2(24.8-33.6) | 12.4(9.5-15.3) |
| Indonesia | 47(40.9-53) | 42.1(24.8-59.3) | 17.5(7-28) |  | 36.6(24.6-48.6) | 11.3(7.2-15.4) | .9(.4-1.3) |
| Laos | 32.2(-3.2-67.5) | 61.6(55.9-67.2) | 14.3(7.4-21.2) |  | 84.2(28.8-139.6) | 9.6(5-14.3) | .6(.4-.8) |
| Myanmar | 68.3(-17-153.5) | 81.4(38.9-123.9) | 47.1(34.1-60) |  | 82.4(71.2-93.5) | 53.2(38.4-67.9) | 30.9(17.8-44) |
| Papua New Guinea | 48.4(16.3-80.5) | 58.3(49.2-67.4) | 32(27.6-36.4) |  | 73.7(41.7-105.6) | 60.4(52.6-68.2) | 43.7(40.9-46.5) |
| Philippines | 58.5(38.5-78.4) | 37.3(18.7-55.9) | 8.8(2.7-14.8) |  | 44.1(-7.8-95.9) | 68.2(14.2-122.2) | 67.7(34.7-100.7) |
| Timor-Leste | 20.2(.003-40.3) | 61.2(52.8-69.7) | 18.5(12.5-24.5) |  | 56.8(20-93.6) | 7.1(.8-13.4) | 1.1(.2-2) |
| Vietnam | 88.6(16.2-161) | 5(-2.3-12.4) | .5(-.2-1.1) |  | 16.1(-1.1-33.2) | 12.5(-1.2-26.1) | 12.3(-.8-25.4) |
| **Eastern and Southern Africa** |  |  |  |  |  |  |  |
| Angola | 58.1(56.5-59.7) | 61.7(59.5-63.9) | 55.9(50.9-60.9) |  | 76.7(56.6-96.7) | 37.9(24.4-51.5) | 14.3(7.4-21.1) |
| Burundi | 32.2(27-37.4) | 13.2(5.6-20.8) | 1(.3-1.6) |  | 76.4(64-88.8) | 31(21.1-40.9) | 11.7(6.5-16.8) |
| Comoros | 52(50.2-53.7) | 23.3(11.9-34.7) | 11.2(6.1-16.3) |  | 4.7(1.5-7.8) | .6(.04-1.2) | .2(.1-.4) |
| Ethiopia | 15.8(-9.5-41.1) | 73.3(9.8-136.7) | 38.1(30.2-46) |  | 26.9(-11.1-64.9) | 72.7(13.1-132.4) | 61.3(44.3-78.3) |
| Kenya | 37.8(26.2-49.4) | 30(16.6-43.3) | 19.4(9.8-29) |  | 100.25(43-157.5) | 42.3(26.6-57.9) | 13(6-20) |
| Lesotho | 47.4(40.6-54.1) | 22.8(12-33.7) | 8.1(3.7-12.6) |  | 83.6(34.6-132.5) | 75.3(74.4-76.10 | 47.9(34.3-61.5) |
| Madagascar | 38.7(12.4-65) | 46.6(36.2-57 | 27.8(24-31.5) |  | 45.4(29.6-61.2) | 59.3(55.2-63.5) | 44(36-52) |
| Malawi | 50.2(28.8-71.6) | 7.9(6.4-9.5) | 1(.7-1.2) |  | 69.8(55.8-83.8) | 17.4(5.7-29.2) | 5.8(1.4-10.2) |
| Mozambique | 75.6(53.4-97.8) | 47(37.3-56.7) | 25.4(16.7-34) |  | 59.9(39.8-79.9) | 24.1(15.5-32.7) | 5.5(2.9-8.1) |
| Rwanda | 38(32.7-43.3) | 3.5(1.5-5.6) | .1(.04-.3) |  | 61.8(49.6-74) | 18.4(6.8-30) | 4.4(1.3-7.5) |
| Tanzania | 64.3(32.9-95.6) | 47.6(42.4-52.8) | 29.2(25.3-33.1) |  | 50.7(42-59.4) | 35.5(21-50.1) | 13.7(6.9-20.4) |
| Uganda | 33.6(9.8-57.4) | 19.6(12.1-27) | 7.8(5.3-10.2) |  | .8(.2-1.4) | .4(-.1-.8) | .2(.1-.4) |
| Zambia | 61.9(41.9-81.9) | 28(19.4-36.6) | 7.5(4.4-10.6) |  | 48(7.4-88.5) | 66.5(55.5-77.5) | 20.7(9.1-32.3) |
| Zimbabwe | 53.8(31.8-75.7) | 26.7(23-30.3) | 10.1(7.9-12.3) |  | 50.6(34.3-66.9) | 21.9(9.5-34.2) | 7(2.6-11.5) |
| **West and Central Africa** |  |  |  |  |  |  |  |
| Benin | 48(38.6-57.4) | 23.7(13.8-33.6) | 12.9(7-18.7) |  | 59.6(50.4-68.8) | 45.9(35.7-56) | 25.5(17.6-33.5) |
| Burkina Faso | 75.2(27.2-123.1) | 22.7(15.9-29.5) | 5.3(3.3-7.3) |  | 80.9(51-110.8) | 76.3(51.6-101) | 64.5(48.5-80.4) |
| Cameroon | 73(69.2-76.8) | 61.1(45.1-77.1) | 48.5(33.1-63.8) |  | 42.5(23.6-61.4) | 10.6(9.6-11.6) | 2.2(2-2.4) |
| Central African Republic | 63.9(41.7-86) | 58.4(49.1-67.7) | 44.2(37.3-51.1) |  | 6.5(-3.4-16.5) | .1(-.1-.3) | 0 |
| Chad | 28.5(-9.3-66.3) | 66.2(9.1-123.2) | 62.9(34.8-91) |  | 72.1(38.5-105.6) | 53.8(51.3-56.3) | 30.1(25.5-34.8) |
| Congo | 51.4(31-71.7) | 9.1(2.3-15.9) | 2.3(.5-4.2) |  | 67.8(48.7-86.9) | 48.2(45.7-50.8) | 31(26.5-35.4) |
| Cote d'Ivoire | 74.6(47.8-101.4) | 42(27.7-56.3) | 16.4(8.4-24.3) |  | 90.4(30.9-149.9) | 60.9(47-74.7) | 31.5(20.6-42.3) |
| Democratic Republic of the Congo | 57.7(49.3-66.1) | 45(38.3-51.7) | 36.6(30.9-42.3) |  | 38.6(.2-77) | 44.9(41.1-48.7) | 8.3(5.7-10.8) |
| Ghana | 99.1(41.4-156.8) | 42.1(26.1-58) | 13.5(5.9-21.1) |  | 45.1(-1.4-91.5) | 52.8(17.7-87.8) | 39.9(20.5-59.3) |
| Guinea | 55.9(26.1-85.7) | 64.1(60.7-67.5) | 42.1(30.8-53.4) |  | 67.7(29.1-106.3) | 78.2(54.3-102.1) | 75.1(67-83.3) |
| Liberia | 37.1(25.5-48.6) | 35.2(27.8-42.7) | 11.4(7.2-15.5) |  | 52.7(19.1-86.3) | 42.5(38.6-46.4) | 18.5(15.9-21.1) |
| Mali | 72.6(39.3-105.9) | 53.9(48.7-59) | 28.4(20.1-36.7) |  | 59.1(21.5-96.6) | 65.4(58.1-72.7) | 37.4(32.2-42.6) |
| Niger | 35.8(-14.2-85.7) | 60.4(16.5-104.3) | 32.8(19.5-46.2) |  | 95.7(76.3-115.1) | 23(1-45) | 3.6(-.3-7.5) |
| Nigeria | 60.2(23.2-97.2) | 71.6(43.8-99.4) | 71.9(56.5-87.2) |  | 42.8(39.2-46.4) | 3.8(1.8-5.9) | .2(.03-.4) |
| Sao Tome and Principe | 60.2(53.6-66.7) | 11(4.8-17.1) | 4.1(1.9-6.2) |  | 26.4(-.3-53.1) | 21.5(-.8-43.7) | 19.8(-.4-40) |
| Senegal | 91.4(88.1-94.6) | 37.1(11.7-62.4) | 13(2.4-23.5) |  | 73.8(68.9-78.7) | 64.2(58.3-70.2) | 55.4(46.6-64.3) |
| Sierra Leone | 9.5(5.9-13.2) | 17.8(15.7-19.8) | 2.6(1.9-3.3) |  | 41.9(29.1-54.6) | 29.7(28.3-31.1) | 13.1(11-15.1) |
| The Gambia | 59.8(-2.4-122) | 23.2(18.3-28.1) | 2.6(1.7-3.5) |  | 23.2(18.7-27.8) | 11(7.8-14.1) | 5.7(3.8-7.6) |
| Togo | 65.7(43.6-87.7) | 72.5(52.7-92.3) | 73(65.1-81) |  | 63.8(32.9-94.7) | 46(43.1-48.9) | 25.6(22.5-28.7) |
| **Latin America and Caribbean** |  |  |  |  |  |  |  |
| Dominican Republic | 9.1(3.3-14.8) | .5(.1-.9) | .1(.04-.3) |  | 67.8(4-131.5) | 24(19.5-28.5) | 3.1(2.1-4.1) |
| Haiti | 41.5(25.9-57.1) | 57.4(54.2-60.5) | 44.8(37.6-51.9) |  | 31.8(17.4-46.2) | 51.8(41.6-61.9) | 16.2(9.2-23.2) |
| Honduras | 70.6(64.3-76.8) | 13.9(4.6-23.2) | 3.9(1.2-6.5) |  | 94.2(76.2-112.2) | 77.2(64.3-90.1) | 57.3(42.5-72.1) |
| **Central and Eastern Europe** |  |  |  |  |  |  |  |
| Albania | 2.2(.7-3.6) | .6(.04-1.2) | .4(.2-.5) |  | 36.3(17.7-54.9) | 22(17.4-26.6) | 9.5(8.2-10.8) |
| Armenia | .4(-.1-.8) | .1(.04-.3) | .1(-.1-.3) |  | 85.6(12.4-158.9) | 5.6(-2.4-13.7) | .5(-.3-1.4) |
| Kyrgyzstan | 1.9(.5-3.3) | .4(-.1-.8) | .1(.04-.3) |  | 66.9(49.5-84.3) | 35.9(24.5-47.2) | 11.2(6-16.4) |
| Tajikistan | 48.2(21-75.4) | 11.7(9.3-14.2) | 3.2(2.6-3.8) |  | 57.3(36.9-77.6) | 28.9(26-31.7) | 11.5(9.6-13.4) |

INSD, institutional delivery; SBA, skilled birth attendants;

Table 10: Determinants of access to facility delivery and skilled birth attendants at births among women aged 20-35 years, 54 low-and middle-income countries

| Characteristics | Odds ratio (95% Credible Interval) | |
| --- | --- | --- |
|  | INSD | SBA |
| HH head age (years) |  |  |
| <30 | 1.00 | 1.00 |
| 30-45 | 1.06 (1.05-1.07) | 1.07 (1.05-1.09) |
| 46-60 | 1.02 (1.01-1.04) | 1.01 (0.98-1.04) |
| >60 | 1.09 (1.07-1.12) | 1.12 (1.09-1.15) |
| HH head sex |  |  |
| Male | 1.00 | 1.00 |
| Female | 1.13 (1.11-1.15) | 1.13 (1.11-1.15) |
| Mother education |  |  |
| No education | 1.00 | 1.00 |
| Primary | 1.20 (1.19-1.22) | 1.21 (1.19-1.24) |
| Secondary | 1.72 (1.69-1.75) | 1.72 (1.68-1.76) |
| Higher | 2.87 (2.82-2.92) | 3.18 (3.06-3.27) |
| Parity |  |  |
| 1 | 1.00 | 1.00 |
| 2 | 0.63 (0.62-0.65) | 0.67 (0.65-0.68) |
| 3 | 0.46 (0.46-0.47) | 0.49 (0.49-0.50) |
| ANC visits |  |  |
| None | 1.00 | 1.00 |
| 1 | 2.18 (2.14-2.22) | 2.18 (2.14-2.24) |
| 2 | 2.69 (2.64-2.74) | 2.69 (2.65-2.74) |
| 3 | 3.53 (3.47-3.60) | 3.60 (3.51-3.69) |
| >=4 | 5.76 (5.67-5.84) | 5.92 (5.80-6.03) |
| Mass media (watch/listen) |  |  |
| None | 1.00 | 1.00 |
| Less than once a week | 1.11 (1.09-1.13) | 1.13 (1.10-1.16) |
| At least once a week | 1.29 (1.27-1.32) | 1.31 (1.29-1.34) |
| Wealth quintile |  |  |
| Q1 (Poorest) | 1.00 | 1.00 |
| Q2 | 1.25 (1.23-1.27) | 1.26 (1.23-1.29) |
| Q3 | 1.68 (1.64-1.71) | 1.69 (1.65-1.73) |
| Q4 | 2.30 (2.26-2.34) | 2.40 (2.32-2.47) |
| Q5 (Richest) | 3.96 (3.86-4.05) | 4.15 (3.98-4.32) |
| Area of residence |  |  |
| Urban | 1.00 | 1.00 |
| Rural | 0.65 (0.64-0.66) | 0.64 (0.62-0.66) |
| Random intercept var(u_0i_)= σ^2^_u0_ | 1.72 (1.05-2.74) | 2.27 (1.36-3.69) |

HH, household; INSD, institutional delivery; SBA, skilled birth attendants;

Table 11: Determinants of access to facility delivery and skilled birth attendants at births among women aged 36 years or more, 54 low-and middle-income countries

| Characteristics | Odds ratio (95% Credible Interval) | |
| --- | --- | --- |
|  | INSD | SBA |
| HH head age (years) |  |  |
| <30 | 1.00 | 1.00 |
| 30-45 | 1.14 (1.06-1.23) | 1.36 (1.31-1.41) |
| 46-60 | 1.00 (0.95-1.06) | 1.24 (1.19-1.30) |
| >60 | 1.03 (0.97-1.10) | 1.28 (1.23-1.33) |
| HH head sex |  |  |
| Male | 1.00 | 1.00 |
| Female | 1.02 (0.98-1.07) | 1.05 (1.00-1.09) |
| Mother education |  |  |
| No education | 1.00 | 1.00 |
| Primary | 1.32 (1.26-1.37) | 1.37 (1.31-1.44) |
| Secondary | 1.91 (1.83-1.99) | 2.07 (1.95-2.19) |
| Higher | 3.55 (3.28-3.81) | 3.76 (3.54-4.01) |
| Parity |  |  |
| 1 | 1.00 | 1.00 |
| 2 | 0.71 (0.68-0.75) | 0.75 (0.72-0.79) |
| 3 | 0.39 (0.38-0.41) | 0.42 (0.39-0.44) |
| ANC visits |  |  |
| None | 1.00 | 1.00 |
| 1 | 2.99 (2.88-3.10) | 2.66 (2.49-2.85) |
| 2 | 4.05 (3.83-4.27) | 4.03 (3.88-4.20) |
| 3 | 5.20 (4.91-5.53) | 5.08 (4.84-5.33) |
| >=4 | 8.68 (8.27-9.11) | 8.41 (8.03-8.85) |
| Mass media (watch/listen) |  |  |
| None | 1.00 | 1.00 |
| Less than once a week | 1.14 (1.08-1.20) | 1.17 (1.14-1.20) |
| At least once a week | 1.28 (1.23-1.33) | 1.32 (1.27-1.37) |
| Wealth quintile |  |  |
| Q1 (Poorest) | 1.00 | 1.00 |
| Q2 | 1.35 (1.31-1.39) | 1.33 (1.27-1.39) |
| Q3 | 1.69 (1.62-1.75) | 1.74 (1.68-1.80) |
| Q4 | 2.54 (2.40-2.67) | 2.70 (2.57-2.80) |
| Q5 (Richest) | 4.42 (4.22-4.63) | 4.91 (4.59-5.24) |
| Area of residence |  |  |
| Urban | 1.00 | 1.00 |
| Rural | 0.57 (0.54-0.60) | 0.58 (0.56-0.60) |
| Random intercept var(u_0i_)= σ^2^_u0_ | 1.50 (0.89-2.48) | 1.44 (0.83-2.42) |

HH, household; INSD, institutional delivery; SBA, skilled birth attendants;

Table 12: Posterior mean difference by considering with and without country level predictors for INSD

| Country | Posterior mean differences by changing predictors (with and without country level predictors) | | | | | | |
| --- | --- | --- | --- | --- | --- | --- | --- |
|  | 2000 | 2005 | 2010 | 2015 | 2020 | 2025 | 2030 |
| Afghanistan | -0.10 | -0.30 | -0.10 | 0.70 | 1.80 | 2.00 | 1.40 |
| Albania | 3.40 | 0.80 | 0.10 | 0.00 | 0.00 | 0.00 | 0.00 |
| Angola | 0.10 | 0.40 | 0.60 | 0.60 | 0.60 | 0.30 | -0.10 |
| Armenia | 0.00 | 0.00 | 0.00 | 0.00 | 0.00 | 0.00 | 0.00 |
| Bangladesh | -0.20 | -0.10 | 0.20 | 0.90 | 1.80 | 2.50 | 2.50 |
| Benin | 1.10 | 0.50 | 0.00 | -0.20 | -0.40 | -0.60 | -0.80 |
| Burkina Faso | 1.50 | 0.10 | -1.30 | -1.90 | -1.90 | -1.80 | -1.40 |
| Burundi | 0.00 | -0.40 | -0.60 | -0.40 | -0.20 | -0.10 | 0.00 |
| Cambodia | 0.00 | -0.10 | -0.20 | -0.10 | -0.10 | 0.00 | 0.00 |
| Cameroon | 0.60 | 0.10 | -0.40 | -0.60 | -0.80 | -0.80 | -0.50 |
| Central African Republic | 3.40 | 1.20 | -1.20 | -3.40 | -5.30 | -6.50 | -7.10 |
| Chad | -0.30 | -0.10 | 0.20 | 0.80 | 1.60 | 2.80 | 4.00 |
| Comoros | 2.10 | 1.20 | 0.10 | -0.60 | -1.00 | -1.20 | -1.20 |
| Congo | 0.20 | 0.00 | 0.00 | 0.10 | 0.10 | 0.20 | 0.20 |
| Cote d'Ivoire | 0.90 | 0.70 | 0.40 | 0.30 | 0.10 | 0.00 | 0.00 |
| DRC | 0.70 | 0.40 | 0.00 | -0.30 | -0.40 | -0.60 | -0.70 |
| Dominican Republic | 1.30 | 0.30 | 0.00 | -0.20 | -0.20 | -0.20 | -0.10 |
| Ethiopia | -0.10 | -0.20 | 0.10 | 0.90 | 2.30 | 3.80 | 4.20 |
| Ghana | 0.50 | 0.40 | 0.40 | 0.30 | 0.10 | 0.10 | 0.10 |
| Guinea | 0.20 | 0.10 | -0.10 | -0.10 | 0.00 | 0.00 | 0.10 |
| Haiti | 0.90 | 0.50 | 0.00 | -0.40 | -0.40 | 0.20 | 1.30 |
| Honduras | 2.50 | 1.10 | -0.20 | -0.80 | -1.00 | -0.90 | -0.70 |
| India | -0.30 | 0.00 | 0.50 | 0.70 | 0.80 | 0.70 | 0.50 |
| Indonesia | 0.00 | -0.10 | -0.20 | -0.30 | -0.20 | -0.20 | -0.10 |
| Kenya | 0.40 | 0.40 | 0.20 | -0.30 | -0.80 | -1.60 | -2.40 |
| Kyrgyzstan | 0.10 | 0.00 | 0.00 | 0.00 | 0.00 | 0.00 | 0.00 |
| Laos | 0.00 | 0.00 | -0.10 | -0.20 | -0.10 | 0.00 | 0.10 |
| Lesotho | 0.30 | -0.10 | -0.40 | -0.40 | -0.40 | -0.20 | -0.10 |
| Liberia | -0.30 | -0.30 | -0.20 | 0.00 | 0.20 | 0.20 | 0.10 |
| Madagascar | 2.00 | 0.80 | -1.40 | -3.70 | -5.10 | -5.30 | -4.60 |
| Malawi | -0.10 | 0.10 | 0.20 | 0.10 | 0.10 | 0.00 | 0.00 |
| Maldives | -0.60 | -0.40 | -0.20 | 0.00 | 0.00 | 0.00 | 0.00 |
| Mali | 0.90 | 0.70 | 0.50 | 0.30 | 0.30 | 0.40 | 0.40 |
| Mozambique | 0.10 | 0.20 | 0.30 | 0.40 | 0.40 | 0.40 | 0.40 |
| Myanmar | 1.10 | 0.80 | 0.20 | -0.60 | -1.50 | -2.40 | -3.30 |
| Nepal | -0.40 | -0.40 | -0.10 | 0.60 | 1.20 | 1.10 | 0.90 |
| Niger | 0.10 | 0.10 | 0.10 | 0.20 | 0.20 | 0.20 | 0.20 |
| Nigeria | -0.40 | -0.30 | -0.10 | 0.00 | 0.10 | 0.30 | 0.50 |
| Pakistan | 0.80 | 0.50 | 0.20 | -0.20 | -0.50 | -0.70 | -0.70 |
| Papua New Guinea | 4.70 | 4.00 | 2.70 | 0.80 | -1.20 | -2.40 | -3.00 |
| Philippines | 0.50 | 0.60 | 0.40 | 0.20 | -0.10 | -0.20 | -0.20 |
| Rwanda | 1.50 | 0.60 | -0.10 | -0.30 | -0.20 | 0.00 | 0.00 |
| Sao Tome and Principe | 1.60 | 0.80 | 0.20 | 0.10 | 0.00 | 0.00 | 0.00 |
| Senegal | 0.60 | 0.40 | 0.10 | -0.10 | -0.20 | -0.30 | -0.40 |
| Sierra Leone | 0.10 | 0.30 | 0.50 | 0.70 | 0.50 | 0.30 | 0.20 |
| Tajikistan | 0.60 | 0.00 | -0.20 | -0.20 | -0.10 | 0.00 | 0.00 |
| Tanzania | -0.40 | -0.30 | -0.30 | -0.20 | -0.20 | -0.10 | 0.00 |
| The Gambia | 0.30 | 0.20 | 0.10 | 0.10 | 0.10 | 0.20 | 0.20 |
| Timor-Leste | -0.60 | -0.90 | -1.10 | -0.60 | 0.50 | 1.30 | 1.40 |
| Togo | 0.40 | 0.10 | -0.20 | -0.60 | -0.80 | -1.00 | -1.10 |
| Uganda | 0.50 | 0.30 | 0.20 | 0.10 | 0.20 | 0.10 | 0.20 |
| Vietnam | 0.40 | 0.00 | -0.30 | -0.50 | -0.80 | -1.10 | -1.40 |
| Zambia | -0.40 | -0.40 | -0.30 | 0.00 | 0.10 | 0.30 | 0.20 |
| Zimbabwe | 2.00 | 1.10 | 0.30 | -0.30 | -0.60 | -0.60 | -0.50 |
| All countries (median) | 0.35 | 0.10 | 0.00 | -0.05 | -0.05 | 0.00 | 0.00 |
| All countries (mean) | 0.62 | 0.28 | -0.01 | -0.17 | -0.24 | -0.25 | -0.23 |

Table 13: Posterior mean difference by considering with and without country level predictors for SBA

| **Country** | Posterior mean differences by changing predictors A(with and without country level predictors) | | | | | | |
| --- | --- | --- | --- | --- | --- | --- | --- |
|  | **2000** | **2005** | **2010** | **2015** | **2020** | **2025** | **2030** |
| Afghanistan | -0.30 | -0.40 | 0.00 | 1.30 | 2.80 | 3.30 | 2.80 |
| Albania | 0.70 | 0.20 | 0.00 | 0.00 | 0.00 | 0.00 | 0.00 |
| Angola | 3.20 | 3.10 | 3.10 | 3.10 | 3.20 | 2.60 | 1.50 |
| Armenia | 0.00 | 0.00 | 0.00 | 0.00 | 0.00 | 0.00 | 0.00 |
| Bangladesh | -0.20 | -0.10 | 0.50 | 1.70 | 3.00 | 3.70 | 3.50 |
| Benin | 2.20 | 1.10 | 0.20 | -0.30 | -0.30 | 0.00 | 0.50 |
| Burkina Faso | 1.30 | 0.20 | -0.90 | -1.50 | -1.50 | -1.50 | -1.30 |
| Burundi | -1.10 | -1.40 | -0.80 | -0.10 | 0.30 | 0.20 | 0.10 |
| Cambodia | 0.00 | -0.20 | -0.40 | -0.10 | -0.10 | 0.00 | 0.00 |
| Cameroon | 0.70 | 0.30 | 0.10 | 0.20 | 0.50 | 1.10 | 1.60 |
| Central African Republic | 1.30 | 0.00 | -1.20 | -2.20 | -2.90 | -3.10 | -3.00 |
| Chad | -0.10 | 0.10 | 0.30 | 0.70 | 1.30 | 2.10 | 2.90 |
| Comoros | 0.20 | -0.20 | -0.20 | -0.20 | -0.10 | 0.10 | 0.10 |
| Congo | -0.20 | -0.20 | -0.10 | 0.10 | 0.30 | 0.40 | 0.50 |
| Cote d'Ivoire | 0.20 | -0.10 | -0.20 | -0.10 | 0.10 | 0.20 | 0.30 |
| Democratic Republic of the Congo | 1.20 | 0.50 | 0.00 | -0.20 | -0.40 | -0.30 | -0.30 |
| Dominican Republic | 1.00 | 0.30 | 0.00 | -0.10 | -0.20 | -0.10 | -0.10 |
| Ethiopia | -0.40 | -0.30 | 0.10 | 1.20 | 2.80 | 4.70 | 6.00 |
| Ghana | -0.10 | 0.10 | 0.20 | 0.50 | 0.70 | 0.70 | 0.70 |
| Guinea | 0.30 | 0.20 | 0.00 | -0.10 | -0.10 | 0.00 | 0.20 |
| Haiti | 2.70 | 1.60 | 0.50 | -0.80 | -1.70 | -2.00 | -1.60 |
| Honduras | 3.40 | 1.20 | -0.60 | -1.40 | -1.80 | -1.70 | -1.50 |
| India | -0.20 | 0.00 | 0.30 | 0.40 | 0.40 | 0.30 | 0.20 |
| Indonesia | -0.40 | -0.40 | -0.30 | -0.10 | 0.00 | 0.20 | 0.20 |
| Kenya | -0.70 | -0.80 | -0.30 | 0.50 | 1.30 | 1.60 | 1.40 |
| Kyrgyzstan | 0.10 | 0.00 | 0.00 | 0.00 | 0.00 | 0.00 | 0.10 |
| Laos | -0.10 | -0.20 | -0.10 | 0.10 | 0.50 | 0.80 | 1.00 |
| Lesotho | 1.20 | 0.50 | 0.20 | 0.10 | 0.10 | 0.10 | 0.20 |
| Liberia | 5.00 | 2.70 | 0.20 | -2.00 | -3.60 | -4.50 | -4.50 |
| Madagascar | 1.20 | -0.80 | -2.50 | -3.60 | -3.90 | -3.50 | -2.60 |
| Malawi | 0.90 | 0.70 | 0.30 | 0.00 | -0.20 | -0.10 | -0.10 |
| Maldives | -1.80 | -0.60 | -0.10 | 0.00 | 0.00 | 0.00 | 0.00 |
| Mali | 0.30 | 0.10 | 0.00 | 0.10 | 0.50 | 0.80 | 1.30 |
| Mozambique | 0.40 | 0.50 | 0.50 | 0.60 | 0.60 | 0.50 | 0.50 |
| Myanmar | 1.90 | 1.10 | 0.40 | -0.30 | -1.00 | -1.50 | -2.10 |
| Nepal | -0.60 | -0.50 | 0.10 | 1.10 | 1.90 | 2.00 | 1.70 |
| Niger | 0.00 | -0.10 | 0.00 | 0.00 | 0.10 | 0.30 | 0.60 |
| Nigeria | -0.20 | -0.30 | -0.40 | -0.50 | -0.60 | -0.60 | -0.60 |
| Pakistan | 0.50 | 0.20 | -0.20 | -0.60 | -0.80 | -0.80 | -0.80 |
| Papua New Guinea | 6.20 | 5.20 | 3.50 | 1.30 | -0.90 | -2.40 | -3.20 |
| Philippines | 1.70 | 0.90 | 0.00 | -0.70 | -1.00 | -1.00 | -0.90 |
| Rwanda | 1.40 | 0.50 | 0.00 | -0.10 | 0.00 | 0.00 | 0.10 |
| Sao Tome and Principe | -0.30 | -0.40 | -0.20 | -0.10 | 0.10 | 0.10 | 0.20 |
| Senegal | 0.20 | 0.00 | -0.10 | -0.30 | -0.50 | -0.80 | -1.00 |
| Sierra Leone | 0.70 | 0.30 | -0.10 | -0.30 | -0.40 | -0.40 | -0.30 |
| Tajikistan | 0.30 | -0.10 | -0.10 | 0.00 | 0.00 | 0.20 | 0.30 |
| Tanzania | 0.00 | 0.00 | -0.10 | -0.10 | 0.10 | 0.20 | 0.40 |
| The Gambia | -0.30 | -0.40 | -0.30 | 0.10 | 0.40 | 0.70 | 0.70 |
| Timor-Leste | -1.00 | -1.50 | -1.50 | -0.50 | 0.90 | 1.60 | 1.50 |
| Togo | 0.60 | 0.20 | 0.00 | -0.10 | -0.10 | 0.00 | 0.10 |
| Uganda | 0.30 | -0.10 | -0.30 | -0.40 | -0.30 | -0.10 | 0.10 |
| Vietnam | -0.30 | -0.30 | -0.30 | -0.20 | -0.20 | -0.10 | -0.10 |
| Zambia | 0.50 | 0.30 | 0.30 | 0.30 | 0.40 | 0.40 | 0.50 |
| Zimbabwe | 3.60 | 1.50 | -0.10 | -1.30 | -1.80 | -1.80 | -1.60 |
| All countries (median) | 0.30 | 0.00 | 0.00 | -0.10 | 0.00 | 0.00 | 0.10 |
| All countries (mean) | 0.65 | 0.25 | -0.01 | -0.09 | -0.06 | 0.02 | 0.10 |

Table 14: Posterior mean difference by altering prior distribution on hyperparameters for INSD

| Country | Posterior mean differences by changing priors (vague vs weekly) | | | | | | |
| --- | --- | --- | --- | --- | --- | --- | --- |
|  | 2000 | 2005 | 2010 | 2015 | 2020 | 2025 | 2030 |
| Afghanistan | -0.10 | -0.20 | -0.30 | -0.60 | -0.70 | -0.50 | -0.40 |
| Albania | -0.80 | -0.30 | -0.10 | 0.00 | 0.00 | 0.00 | 0.00 |
| Angola | -0.30 | -0.30 | -0.40 | -0.40 | -0.30 | -0.20 | -0.10 |
| Armenia | 0.00 | 0.00 | 0.00 | 0.00 | 0.00 | 0.00 | 0.00 |
| Bangladesh | 0.00 | 0.00 | -0.20 | -0.40 | -0.70 | -0.70 | -0.70 |
| Benin | 0.30 | 0.10 | -0.10 | -0.10 | -0.20 | -0.20 | -0.10 |
| Burkina Faso | -0.40 | -0.10 | 0.20 | 0.50 | 0.50 | 0.40 | 0.30 |
| Burundi | 0.30 | 0.20 | 0.00 | -0.10 | -0.10 | -0.10 | -0.10 |
| Cambodia | -0.20 | -0.40 | -0.60 | -0.40 | -0.20 | -0.10 | -0.10 |
| Cameroon | -0.40 | -0.50 | -0.70 | -0.80 | -1.00 | -1.30 | -1.30 |
| Central African Republic | -0.20 | -0.20 | -0.10 | -0.10 | -0.10 | -0.10 | 0.00 |
| Chad | 0.10 | 0.20 | 0.00 | 0.10 | -0.10 | -0.30 | -0.60 |
| Comoros | 0.10 | -0.10 | -0.20 | -0.10 | -0.20 | -0.30 | -0.30 |
| Congo | -0.40 | -0.30 | -0.20 | -0.10 | -0.20 | -0.20 | -0.20 |
| Cote d'Ivoire | 0.90 | 0.60 | 0.20 | -0.20 | -0.60 | -0.80 | -0.90 |
| Democratic Republic of the Congo | 0.20 | 0.10 | -0.10 | -0.20 | -0.20 | -0.40 | -0.40 |
| Dominican Republic | -0.20 | -0.10 | 0.00 | 0.00 | 0.00 | 0.00 | 0.00 |
| Ethiopia | 0.00 | 0.00 | 0.10 | 0.30 | 0.60 | 0.80 | 0.90 |
| Ghana | 0.40 | 0.30 | 0.10 | 0.00 | -0.20 | -0.30 | -0.20 |
| Guinea | 0.20 | 0.20 | 0.10 | 0.00 | -0.10 | -0.30 | -0.50 |
| Haiti | -0.10 | 0.00 | -0.10 | -0.20 | -0.40 | -0.70 | -1.10 |
| Honduras | -0.20 | 0.00 | 0.10 | 0.20 | 0.30 | 0.30 | 0.30 |
| India | -0.20 | -0.20 | -0.10 | -0.10 | 0.00 | 0.00 | 0.00 |
| Indonesia | 0.20 | 0.30 | 0.30 | 0.40 | 0.40 | 0.30 | 0.20 |
| Kenya | -0.20 | -0.30 | -0.30 | -0.20 | -0.10 | 0.10 | 0.30 |
| Kyrgyzstan | -0.10 | 0.00 | 0.00 | 0.00 | 0.00 | 0.00 | 0.00 |
| Laos | -0.20 | 0.00 | 0.30 | 0.70 | 1.20 | 1.20 | 0.90 |
| Lesotho | -0.50 | -0.30 | -0.10 | 0.00 | 0.00 | 0.20 | 0.20 |
| Liberia | 0.00 | 0.00 | -0.10 | -0.10 | 0.00 | 0.00 | 0.00 |
| Madagascar | 0.10 | 0.20 | -0.10 | -0.40 | -0.60 | -0.60 | -0.60 |
| Malawi | -0.10 | -0.10 | 0.00 | 0.00 | 0.00 | 0.00 | 0.00 |
| Maldives | 1.70 | 0.90 | 0.20 | 0.10 | 0.00 | 0.00 | 0.00 |
| Mali | -0.10 | 0.00 | 0.00 | 0.00 | 0.10 | 0.10 | 0.00 |
| Mozambique | 0.10 | 0.20 | 0.20 | 0.20 | 0.20 | 0.10 | 0.10 |
| Myanmar | -0.50 | -0.30 | 0.00 | 0.20 | 0.80 | 1.20 | 1.60 |
| Nepal | 0.00 | 0.10 | 0.30 | 0.40 | 0.30 | 0.20 | 0.10 |
| Niger | 0.00 | 0.00 | -0.10 | -0.20 | -0.50 | -0.70 | -1.00 |
| Nigeria | 0.10 | 0.10 | 0.20 | 0.20 | 0.10 | 0.10 | 0.10 |
| Pakistan | 0.30 | 0.10 | -0.10 | -0.20 | -0.30 | -0.40 | -0.40 |
| Papua New Guinea | 2.40 | 2.00 | 1.30 | 0.40 | -0.40 | -1.00 | -1.40 |
| Philippines | -0.20 | 0.00 | 0.20 | 0.50 | 0.50 | 0.40 | 0.30 |
| Rwanda | 0.10 | 0.10 | 0.10 | 0.00 | 0.00 | 0.00 | 0.00 |
| Sao Tome and Principe | 1.10 | 0.70 | 0.30 | 0.10 | 0.00 | 0.00 | 0.00 |
| Senegal | 0.00 | -0.10 | -0.30 | -0.40 | -0.50 | -0.40 | -0.50 |
| Sierra Leone | 0.00 | 0.00 | -0.10 | -0.10 | -0.10 | 0.00 | 0.00 |
| Tajikistan | 0.10 | 0.00 | 0.00 | -0.10 | -0.20 | -0.10 | -0.20 |
| Tanzania | -0.80 | -0.50 | -0.30 | 0.00 | 0.10 | 0.20 | 0.20 |
| The Gambia | -0.50 | -0.40 | -0.30 | -0.10 | -0.10 | 0.00 | 0.00 |
| Timor-Leste | 0.00 | -0.10 | -0.10 | 0.00 | 0.00 | 0.00 | 0.20 |
| Togo | -0.20 | -0.20 | -0.20 | -0.30 | -0.20 | -0.10 | 0.00 |
| Uganda | 0.00 | 0.10 | 0.10 | 0.10 | 0.10 | 0.10 | 0.00 |
| Vietnam | -0.20 | -0.20 | -0.10 | 0.00 | 0.00 | 0.10 | 0.10 |
| Zambia | -0.40 | -0.40 | -0.30 | -0.20 | -0.10 | 0.00 | 0.00 |
| Zimbabwe | 0.80 | 0.60 | 0.40 | 0.10 | -0.30 | -0.40 | -0.60 |
| All countries (median) | 0.00 | 0.00 | -0.05 | 0.00 | -0.10 | 0.00 | 0.00 |
| All countries (mean) | 0.03 | 0.02 | -0.02 | -0.02 | -0.05 | -0.07 | -0.09 |

Table 15: Posterior mean difference by altering prior distribution on hyperparameters for SBA

| Country | Posterior mean differences by changing priors (vague vs weekly) | | | | | | |
| --- | --- | --- | --- | --- | --- | --- | --- |
|  | 2000.00 | 2005.00 | 2010.00 | 2015.00 | 2020.00 | 2025.00 | 2030.00 |
| Afghanistan | 0.10 | 0.30 | 0.50 | 0.50 | 0.40 | 0.20 | 0.00 |
| Albania | 0.00 | 0.10 | 0.00 | 0.00 | 0.00 | 0.00 | 0.00 |
| Angola | -0.10 | 0.00 | 0.20 | 0.30 | 0.70 | 1.00 | 1.30 |
| Armenia | 0.00 | 0.00 | 0.00 | 0.00 | 0.00 | 0.00 | 0.00 |
| Bangladesh | 0.20 | 0.20 | 0.30 | 0.40 | 0.40 | 0.40 | 0.40 |
| Benin | 0.80 | 0.60 | 0.40 | 0.20 | 0.10 | 0.00 | 0.10 |
| Burkina Faso | -0.10 | 0.20 | 0.40 | 0.50 | 0.60 | 0.50 | 0.50 |
| Burundi | 0.10 | 0.10 | 0.10 | 0.00 | 0.10 | 0.00 | 0.00 |
| Cambodia | -0.10 | 0.10 | 0.20 | 0.30 | 0.10 | 0.00 | 0.00 |
| Cameroon | -0.10 | 0.00 | 0.10 | 0.20 | 0.30 | 0.40 | 0.50 |
| Central African Republic | -0.20 | 0.00 | 0.10 | 0.40 | 0.50 | 0.70 | 0.80 |
| Chad | 0.10 | 0.10 | 0.00 | -0.10 | -0.20 | -0.30 | -0.50 |
| Comoros | 0.20 | 0.40 | 0.60 | 0.60 | 0.50 | 0.50 | 0.30 |
| Congo | -0.10 | -0.10 | 0.00 | 0.00 | 0.10 | 0.10 | 0.10 |
| Cote d'Ivoire | 0.70 | 0.70 | 0.60 | 0.40 | 0.20 | 0.20 | 0.10 |
| Democratic Republic of the Congo | 0.10 | 0.10 | 0.10 | 0.10 | 0.00 | 0.10 | 0.10 |
| Dominican Republic | 0.00 | 0.10 | 0.00 | 0.10 | 0.00 | 0.00 | -0.10 |
| Ethiopia | 0.00 | -0.10 | -0.20 | -0.40 | -0.80 | -1.10 | -1.30 |
| Ghana | -0.10 | -0.10 | -0.20 | -0.20 | -0.20 | -0.20 | -0.10 |
| Guinea | -0.20 | -0.20 | -0.20 | -0.20 | -0.10 | -0.10 | -0.10 |
| Haiti | 0.50 | 0.40 | 0.30 | 0.00 | -0.40 | -0.80 | -1.30 |
| Honduras | 0.30 | 0.00 | -0.20 | -0.20 | -0.30 | -0.20 | -0.10 |
| India | 0.50 | 0.60 | 0.60 | 0.40 | 0.30 | 0.10 | 0.00 |
| Indonesia | 0.30 | 0.20 | 0.00 | 0.00 | -0.10 | -0.10 | -0.10 |
| Kenya | 0.30 | 0.30 | 0.50 | 0.60 | 0.80 | 0.80 | 0.70 |
| Kyrgyzstan | 0.00 | 0.00 | 0.00 | 0.00 | 0.00 | 0.00 | 0.00 |
| Laos | -0.20 | -0.20 | -0.30 | -0.40 | -0.40 | -0.30 | -0.10 |
| Lesotho | -0.20 | -0.10 | 0.10 | 0.10 | 0.20 | 0.10 | 0.10 |
| Liberia | 0.50 | 0.50 | 0.40 | 0.30 | 0.10 | -0.20 | -0.30 |
| Madagascar | -0.40 | -0.30 | -0.20 | -0.10 | 0.00 | 0.10 | 0.30 |
| Malawi | 0.00 | -0.10 | -0.10 | -0.10 | -0.10 | 0.00 | 0.00 |
| Maldives | -0.10 | -0.10 | 0.00 | 0.00 | 0.00 | 0.00 | 0.00 |
| Mali | 0.10 | 0.30 | 0.40 | 0.40 | 0.50 | 0.40 | 0.40 |
| Mozambique | 0.30 | 0.20 | 0.00 | -0.10 | -0.20 | -0.50 | -0.50 |
| Myanmar | -0.20 | -0.30 | -0.20 | -0.10 | 0.00 | 0.10 | 0.20 |
| Nepal | 0.00 | 0.10 | 0.10 | 0.20 | 0.20 | 0.10 | 0.00 |
| Niger | -0.20 | -0.20 | -0.30 | -0.40 | -0.40 | -0.50 | -0.50 |
| Nigeria | 0.00 | -0.20 | -0.20 | -0.30 | -0.40 | -0.50 | -0.60 |
| Pakistan | -0.10 | -0.20 | -0.30 | -0.50 | -0.50 | -0.50 | -0.70 |
| Papua New Guinea | 2.10 | 1.70 | 1.00 | 0.20 | -0.60 | -1.20 | -1.60 |
| Philippines | -0.40 | -0.30 | -0.30 | -0.20 | -0.10 | 0.10 | 0.10 |
| Rwanda | -0.40 | -0.30 | -0.10 | 0.00 | 0.00 | 0.10 | 0.10 |
| Sao Tome and Principe | -0.10 | 0.00 | 0.00 | -0.10 | -0.10 | -0.10 | 0.00 |
| Senegal | 0.20 | 0.20 | 0.10 | 0.10 | 0.00 | -0.10 | -0.10 |
| Sierra Leone | -0.50 | -0.40 | -0.20 | -0.10 | 0.10 | 0.10 | 0.10 |
| Tajikistan | 0.20 | 0.00 | -0.10 | -0.10 | -0.20 | -0.10 | -0.20 |
| Tanzania | -0.70 | -0.50 | -0.30 | -0.10 | 0.10 | 0.20 | 0.30 |
| The Gambia | 0.10 | 0.00 | -0.20 | -0.30 | -0.40 | -0.30 | -0.30 |
| Timor-Leste | 0.50 | 0.50 | 0.40 | 0.10 | -0.20 | -0.50 | -0.60 |
| Togo | 0.10 | -0.30 | -0.60 | -0.90 | -1.20 | -1.40 | -1.70 |
| Uganda | -0.30 | -0.30 | -0.30 | -0.20 | -0.20 | -0.10 | -0.10 |
| Vietnam | -0.20 | -0.20 | -0.20 | -0.20 | -0.20 | -0.20 | -0.20 |
| Zambia | 0.10 | 0.10 | 0.00 | -0.10 | -0.10 | -0.20 | -0.10 |
| Zimbabwe | 0.40 | 0.40 | 0.30 | 0.10 | 0.00 | -0.10 | -0.20 |
| All countries (median) | 0.00 | 0.00 | 0.00 | 0.00 | 0.00 | 0.00 | 0.00 |
| All countries (mean) | 0.06 | 0.06 | 0.04 | 0.01 | -0.03 | -0.07 | -0.09 |

Table 16: National level estimate of Gelman Rubin Potential scale reduction factors (PSRF) for INSD

| **Country** | Potential scale reduction factors (Point estimate; Upper C.I) | | | | | | |
| --- | --- | --- | --- | --- | --- | --- | --- |
|  | **2000** | **2005** | **2010** | **2015** | **2020** | **2025** | **2030** |
| Afghanistan | (1.001;1.008) | (1.001;1.008) | (1.001;1.008) | (1.001;1.008) | (1.001;1.008) | (1.001;1.008) | (1.001;1.008) |
| Albania | (1.001;1.008) | (1.001;1.008) | (1.001;1.008) | (1.001;1.008) | (1.001;1.008) | (1.001;1.008) | (1.001;1.008) |
| Angola | (1.001;1.008) | (1.001;1.008) | (1.001;1.008) | (1.001;1.008) | (1.001;1.008) | (1.001;1.008) | (1.001;1.008) |
| Armenia | (1.001;1.008) | (1.001;1.008) | (1.001;1.008) | (1.001;1.008) | (1.001;1.008) | (1.001;1.008) | (1.001;1.008) |
| Bangladesh | (1.001;1.008) | (1.001;1.008) | (1.001;1.008) | (1.001;1.008) | (1.001;1.008) | (1.001;1.008) | (1.001;1.008) |
| Benin | (1.001;1.008) | (1.001;1.008) | (1.001;1.008) | (1.001;1.008) | (1.001;1.008) | (1.001;1.008) | (1.001;1.008) |
| Burkina Faso | (1.001;1.008) | (1.001;1.008) | (1.001;1.008) | (1.001;1.008) | (1.001;1.008) | (1.001;1.008) | (1.001;1.008) |
| Burundi | (1.001;1.008) | (1.001;1.008) | (1.001;1.008) | (1.001;1.008) | (1.001;1.008) | (1.001;1.008) | (1.001;1.008) |
| Cambodia | (1.001;1.008) | (1.001;1.008) | (1.001;1.008) | (1.001;1.008) | (1.001;1.008) | (1.001;1.008) | (1.001;1.008) |
| Cameroon | (1.001;1.008) | (1.001;1.008) | (1.001;1.008) | (1.001;1.008) | (1.001;1.008) | (1.001;1.008) | (1.001;1.008) |
| Central African Republic | (1.001;1.008) | (1.001;1.008) | (1.001;1.008) | (1.001;1.008) | (1.001;1.008) | (1.001;1.008) | (1.001;1.008) |
| Chad | (1.001;1.008) | (1.001;1.008) | (1.001;1.008) | (1.001;1.008) | (1.001;1.008) | (1.001;1.008) | (1.001;1.008) |
| Comoros | (1.001;1.008) | (1.001;1.008) | (1.001;1.008) | (1.001;1.008) | (1.001;1.008) | (1.001;1.008) | (1.001;1.008) |
| Congo | (1.001;1.008) | (1.001;1.008) | (1.001;1.008) | (1.001;1.008) | (1.001;1.008) | (1.001;1.008) | (1.001;1.008) |
| Cote d'Ivoire | (1.001;1.008) | (1.001;1.008) | (1.001;1.008) | (1.001;1.008) | (1.001;1.008) | (1.001;1.008) | (1.001;1.008) |
| DRC | (1.001;1.008) | (1.001;1.008) | (1.001;1.008) | (1.001;1.008) | (1.001;1.008) | (1.001;1.008) | (1.001;1.008) |
| Dominican Republic | (1.001;1.008) | (1.001;1.008) | (1.001;1.008) | (1.001;1.008) | (1.001;1.008) | (1.001;1.008) | (1.001;1.008) |
| Ethiopia | (1.001;1.008) | (1.001;1.008) | (1.001;1.008) | (1.001;1.008) | (1.001;1.008) | (1.001;1.008) | (1.001;1.008) |
| Ghana | (1.001;1.008) | (1.001;1.008) | (1.001;1.008) | (1.001;1.008) | (1.001;1.008) | (1.001;1.008) | (1.001;1.008) |
| Guinea | (1.001;1.008) | (1.001;1.008) | (1.001;1.008) | (1.001;1.008) | (1.001;1.008) | (1.001;1.008) | (1.001;1.008) |
| Haiti | (1.001;1.008) | (1.001;1.008) | (1.001;1.008) | (1.001;1.008) | (1.001;1.008) | (1.001;1.008) | (1.001;1.008) |
| Honduras | (1.001;1.008) | (1.001;1.008) | (1.001;1.008) | (1.001;1.008) | (1.001;1.008) | (1.001;1.008) | (1.001;1.008) |
| India | (1.001;1.008) | (1.001;1.008) | (1.001;1.008) | (1.001;1.008) | (1.001;1.008) | (1.001;1.008) | (1.001;1.008) |
| Indonesia | (1.001;1.008) | (1.001;1.008) | (1.001;1.008) | (1.001;1.008) | (1.001;1.008) | (1.001;1.008) | (1.001;1.008) |
| Kenya | (1.001;1.008) | (1.001;1.008) | (1.001;1.008) | (1.001;1.008) | (1.001;1.008) | (1.001;1.008) | (1.001;1.008) |
| Kyrgyzstan | (1.001;1.008) | (1.001;1.008) | (1.001;1.008) | (1.001;1.008) | (1.001;1.008) | (1.001;1.008) | (1.001;1.008) |
| Laos | (1.001;1.008) | (1.001;1.008) | (1.001;1.008) | (1.001;1.008) | (1.001;1.008) | (1.001;1.008) | (1.001;1.008) |
| Lesotho | (1.001;1.008) | (1.001;1.008) | (1.001;1.008) | (1.001;1.008) | (1.001;1.008) | (1.001;1.008) | (1.001;1.008) |
| Liberia | (1.001;1.008) | (1.001;1.008) | (1.001;1.008) | (1.001;1.008) | (1.001;1.008) | (1.001;1.008) | (1.001;1.008) |
| Madagascar | (1.001;1.008) | (1.001;1.008) | (1.001;1.008) | (1.001;1.008) | (1.001;1.008) | (1.001;1.008) | (1.001;1.008) |
| Malawi | (1.001;1.008) | (1.001;1.008) | (1.001;1.008) | (1.001;1.008) | (1.001;1.008) | (1.001;1.008) | (1.001;1.008) |
| Maldives | (1.001;1.008) | (1.001;1.008) | (1.001;1.008) | (1.001;1.008) | (1.001;1.008) | (1.001;1.008) | (1.001;1.008) |
| Mali | (1.001;1.008) | (1.001;1.008) | (1.001;1.008) | (1.001;1.008) | (1.001;1.008) | (1.001;1.008) | (1.001;1.008) |
| Mozambique | (1.001;1.008) | (1.001;1.008) | (1.001;1.008) | (1.001;1.008) | (1.001;1.008) | (1.001;1.008) | (1.001;1.008) |
| Myanmar | (1.001;1.008) | (1.001;1.008) | (1.001;1.008) | (1.001;1.008) | (1.001;1.008) | (1.001;1.008) | (1.001;1.008) |
| Nepal | (1.001;1.008) | (1.001;1.008) | (1.001;1.008) | (1.001;1.008) | (1.001;1.008) | (1.001;1.008) | (1.001;1.008) |
| Niger | (1.001;1.008) | (1.001;1.008) | (1.001;1.008) | (1.001;1.008) | (1.001;1.008) | (1.001;1.008) | (1.001;1.008) |
| Nigeria | (1.001;1.008) | (1.001;1.008) | (1.001;1.008) | (1.001;1.008) | (1.001;1.008) | (1.001;1.008) | (1.001;1.008) |
| Pakistan | (1.001;1.008) | (1.001;1.008) | (1.001;1.008) | (1.001;1.008) | (1.001;1.008) | (1.001;1.008) | (1.001;1.008) |
| Papua New Guinea | (1.001;1.008) | (1.001;1.008) | (1.001;1.008) | (1.001;1.008) | (1.001;1.008) | (1.001;1.008) | (1.001;1.008) |
| Philippines | (1.001;1.008) | (1.001;1.008) | (1.001;1.008) | (1.001;1.008) | (1.001;1.008) | (1.001;1.008) | (1.001;1.008) |
| Rwanda | (1.001;1.008) | (1.001;1.008) | (1.001;1.008) | (1.001;1.008) | (1.001;1.008) | (1.001;1.008) | (1.001;1.008) |
| Sao Tome and Principe | (1.001;1.008) | (1.001;1.008) | (1.001;1.008) | (1.001;1.008) | (1.001;1.008) | (1.001;1.008) | (1.001;1.008) |
| Senegal | (1.001;1.008) | (1.001;1.008) | (1.001;1.008) | (1.001;1.008) | (1.001;1.008) | (1.001;1.008) | (1.001;1.008) |
| Sierra Leone | (1.001;1.008) | (1.001;1.008) | (1.001;1.008) | (1.001;1.008) | (1.001;1.008) | (1.001;1.008) | (1.001;1.008) |
| Tajikistan | (1.001;1.008) | (1.001;1.008) | (1.001;1.008) | (1.001;1.008) | (1.001;1.008) | (1.001;1.008) | (1.001;1.008) |
| Tanzania | (1.001;1.008) | (1.001;1.008) | (1.001;1.008) | (1.001;1.008) | (1.001;1.008) | (1.001;1.008) | (1.001;1.008) |
| The Gambia | (1.001;1.008) | (1.001;1.008) | (1.001;1.008) | (1.001;1.008) | (1.001;1.008) | (1.001;1.008) | (1.001;1.008) |
| Timor-Leste | (1.001;1.008) | (1.001;1.008) | (1.001;1.008) | (1.001;1.008) | (1.001;1.008) | (1.001;1.008) | (1.001;1.008) |
| Togo | (1.001;1.008) | (1.001;1.008) | (1.001;1.008) | (1.001;1.008) | (1.001;1.008) | (1.001;1.008) | (1.001;1.008) |
| Uganda | (1.001;1.008) | (1.001;1.008) | (1.001;1.008) | (1.001;1.008) | (1.001;1.008) | (1.001;1.008) | (1.001;1.008) |
| Vietnam | (1.001;1.008) | (1.001;1.008) | (1.001;1.008) | (1.001;1.008) | (1.001;1.008) | (1.001;1.008) | (1.001;1.008) |
| Zambia | (1.001;1.008) | (1.001;1.008) | (1.001;1.008) | (1.001;1.008) | (1.001;1.008) | (1.001;1.008) | (1.001;1.008) |
| Zimbabwe | (1.001;1.008) | (1.001;1.008) | (1.001;1.008) | (1.001;1.008) | (1.001;1.008) | (1.001;1.008) | (1.001;1.008) |

CI, confidence interval

Table 17: National level estimate of Gelman Rubin Potential scale reduction factors (PSRF) for SBA

| **Country** | Potential scale reduction factors (Point estimate; Upper C.I) | | | | | | |
| --- | --- | --- | --- | --- | --- | --- | --- |
|  | **2000** | **2005** | **2010** | **2015** | **2020** | **2025** | **2030** |
| Afghanistan | (1;1.01) | (1;1.01) | (1;1.01) | (1;1.01) | (1;1.01) | (1;1.01) | (1;1.01) |
| Albania | (1;1.01) | (1;1.01) | (1;1.01) | (1;1.01) | (1;1.01) | (1;1.01) | (1;1.01) |
| Angola | (1;1.01) | (1;1.01) | (1;1.01) | (1;1.01) | (1;1.01) | (1;1.01) | (1;1.01) |
| Armenia | (1;1.01) | (1;1.01) | (1;1.01) | (1;1.01) | (1;1.01) | (1;1.01) | (1;1.01) |
| Bangladesh | (1;1.01) | (1;1.01) | (1;1.01) | (1;1.01) | (1;1.01) | (1;1.01) | (1;1.01) |
| Benin | (1;1.01) | (1;1.01) | (1;1.01) | (1;1.01) | (1;1.01) | (1;1.01) | (1;1.01) |
| Burkina Faso | (1;1.01) | (1;1.01) | (1;1.01) | (1;1.01) | (1;1.01) | (1;1.01) | (1;1.01) |
| Burundi | (1;1.01) | (1;1.01) | (1;1.01) | (1;1.01) | (1;1.01) | (1;1.01) | (1;1.01) |
| Cambodia | (1;1.01) | (1;1.01) | (1;1.01) | (1;1.01) | (1;1.01) | (1;1.01) | (1;1.01) |
| Cameroon | (1;1.01) | (1;1.01) | (1;1.01) | (1;1.01) | (1;1.01) | (1;1.01) | (1;1.01) |
| Central African Republic | (1;1.01) | (1;1.01) | (1;1.01) | (1;1.01) | (1;1.01) | (1;1.01) | (1;1.01) |
| Chad | (1;1.01) | (1;1.01) | (1;1.01) | (1;1.01) | (1;1.01) | (1;1.01) | (1;1.01) |
| Comoros | (1;1.01) | (1;1.01) | (1;1.01) | (1;1.01) | (1;1.01) | (1;1.01) | (1;1.01) |
| Congo | (1;1.01) | (1;1.01) | (1;1.01) | (1;1.01) | (1;1.01) | (1;1.01) | (1;1.01) |
| Cote d'Ivoire | (1;1.01) | (1;1.01) | (1;1.01) | (1;1.01) | (1;1.01) | (1;1.01) | (1;1.01) |
| DRC | (1;1.01) | (1;1.01) | (1;1.01) | (1;1.01) | (1;1.01) | (1;1.01) | (1;1.01) |
| Dominican Republic | (1;1.01) | (1;1.01) | (1;1.01) | (1;1.01) | (1;1.01) | (1;1.01) | (1;1.01) |
| Ethiopia | (1;1.01) | (1;1.01) | (1;1.01) | (1;1.01) | (1;1.01) | (1;1.01) | (1;1.01) |
| Ghana | (1;1.01) | (1;1.01) | (1;1.01) | (1;1.01) | (1;1.01) | (1;1.01) | (1;1.01) |
| Guinea | (1;1.01) | (1;1.01) | (1;1.01) | (1;1.01) | (1;1.01) | (1;1.01) | (1;1.01) |
| Haiti | (1;1.01) | (1;1.01) | (1;1.01) | (1;1.01) | (1;1.01) | (1;1.01) | (1;1.01) |
| Honduras | (1;1.01) | (1;1.01) | (1;1.01) | (1;1.01) | (1;1.01) | (1;1.01) | (1;1.01) |
| India | (1;1.01) | (1;1.01) | (1;1.01) | (1;1.01) | (1;1.01) | (1;1.01) | (1;1.01) |
| Indonesia | (1;1.01) | (1;1.01) | (1;1.01) | (1;1.01) | (1;1.01) | (1;1.01) | (1;1.01) |
| Kenya | (1;1.01) | (1;1.01) | (1;1.01) | (1;1.01) | (1;1.01) | (1;1.01) | (1;1.01) |
| Kyrgyzstan | (1;1.01) | (1;1.01) | (1;1.01) | (1;1.01) | (1;1.01) | (1;1.01) | (1;1.01) |
| Laos | (1;1.01) | (1;1.01) | (1;1.01) | (1;1.01) | (1;1.01) | (1;1.01) | (1;1.01) |
| Lesotho | (1;1.01) | (1;1.01) | (1;1.01) | (1;1.01) | (1;1.01) | (1;1.01) | (1;1.01) |
| Liberia | (1;1.01) | (1;1.01) | (1;1.01) | (1;1.01) | (1;1.01) | (1;1.01) | (1;1.01) |
| Madagascar | (1;1.01) | (1;1.01) | (1;1.01) | (1;1.01) | (1;1.01) | (1;1.01) | (1;1.01) |
| Malawi | (1;1.01) | (1;1.01) | (1;1.01) | (1;1.01) | (1;1.01) | (1;1.01) | (1;1.01) |
| Maldives | (1;1.01) | (1;1.01) | (1;1.01) | (1;1.01) | (1;1.01) | (1;1.01) | (1;1.01) |
| Mali | (1;1.01) | (1;1.01) | (1;1.01) | (1;1.01) | (1;1.01) | (1;1.01) | (1;1.01) |
| Mozambique | (1;1.01) | (1;1.01) | (1;1.01) | (1;1.01) | (1;1.01) | (1;1.01) | (1;1.01) |
| Myanmar | (1;1.01) | (1;1.01) | (1;1.01) | (1;1.01) | (1;1.01) | (1;1.01) | (1;1.01) |
| Nepal | (1;1.01) | (1;1.01) | (1;1.01) | (1;1.01) | (1;1.01) | (1;1.01) | (1;1.01) |
| Niger | (1;1.01) | (1;1.01) | (1;1.01) | (1;1.01) | (1;1.01) | (1;1.01) | (1;1.01) |
| Nigeria | (1;1.01) | (1;1.01) | (1;1.01) | (1;1.01) | (1;1.01) | (1;1.01) | (1;1.01) |
| Pakistan | (1;1.01) | (1;1.01) | (1;1.01) | (1;1.01) | (1;1.01) | (1;1.01) | (1;1.01) |
| Papua New Guinea | (1;1.01) | (1;1.01) | (1;1.01) | (1;1.01) | (1;1.01) | (1;1.01) | (1;1.01) |
| Philippines | (1;1.01) | (1;1.01) | (1;1.01) | (1;1.01) | (1;1.01) | (1;1.01) | (1;1.01) |
| Rwanda | (1;1.01) | (1;1.01) | (1;1.01) | (1;1.01) | (1;1.01) | (1;1.01) | (1;1.01) |
| Sao Tome and Principe | (1;1.01) | (1;1.01) | (1;1.01) | (1;1.01) | (1;1.01) | (1;1.01) | (1;1.01) |
| Senegal | (1;1.01) | (1;1.01) | (1;1.01) | (1;1.01) | (1;1.01) | (1;1.01) | (1;1.01) |
| Sierra Leone | (1;1.01) | (1;1.01) | (1;1.01) | (1;1.01) | (1;1.01) | (1;1.01) | (1;1.01) |
| Tajikistan | (1;1.01) | (1;1.01) | (1;1.01) | (1;1.01) | (1;1.01) | (1;1.01) | (1;1.01) |
| Tanzania | (1;1.01) | (1;1.01) | (1;1.01) | (1;1.01) | (1;1.01) | (1;1.01) | (1;1.01) |
| The Gambia | (1;1.01) | (1;1.01) | (1;1.01) | (1;1.01) | (1;1.01) | (1;1.01) | (1;1.01) |
| Timor-Leste | (1;1.01) | (1;1.01) | (1;1.01) | (1;1.01) | (1;1.01) | (1;1.01) | (1;1.01) |
| Togo | (1;1.01) | (1;1.01) | (1;1.01) | (1;1.01) | (1;1.01) | (1;1.01) | (1;1.01) |
| Uganda | (1;1.01) | (1;1.01) | (1;1.01) | (1;1.01) | (1;1.01) | (1;1.01) | (1;1.01) |
| Vietnam | (1;1.01) | (1;1.01) | (1;1.01) | (1;1.01) | (1;1.01) | (1;1.01) | (1;1.01) |
| Zambia | (1;1.01) | (1;1.01) | (1;1.01) | (1;1.01) | (1;1.01) | (1;1.01) | (1;1.01) |
| Zimbabwe | (1;1.01) | (1;1.01) | (1;1.01) | (1;1.01) | (1;1.01) | (1;1.01) | (1;1.01) |

CI, confidence interval
